# Preliminary checklist of spiders (Araneae) from Coiba National Park, Panama

**DOI:** 10.3897/BDJ.12.e117642

**Published:** 2024-07-31

**Authors:** Daniel Murcia-Moreno, Dumas Gálvez

**Affiliations:** 1 Coiba Scientific Station, Panama, Panama Coiba Scientific Station Panama Panama; 2 Smithsonian Tropical Research Institute, Panama, Panama Smithsonian Tropical Research Institute Panama Panama; 3 Programa Centroamericano de Maestría en Entomología, Universidad de Panamá, Ciudad de Panamá, Panama, Panama Programa Centroamericano de Maestría en Entomología, Universidad de Panamá, Ciudad de Panamá Panama Panama

**Keywords:** Araneae, checklist, Coiba, inventory, island

## Abstract

**Background:**

Coiba National Park is an offshore region on the Pacific side of Panama, which hosts several endemic species of animals and plants. It was declared a UNESCO World Heritage Site in 2005. Despite the title awarded to the Park, knowledge about basic elements of its biodiversity are still lacking, which are of vital relevance for management and conservation policies. For instance, until now, no study had ever monitored the araneofauna diversity of the Park.

**New information:**

Here, we provide the first checklist of spider species in Coiba National Park, including the main island and several surrounding islands. We sampled during several field trips carried out from August 2021 to August 2023. We identified at least 152 species (98 genera and 30 families) and we report three new spiders species for Panama, namely *Ctenusnigrolineatus* Berland (1913), *Chapodagitae* Zhang & Maddison (2012) and *Sarindanigra* Peckham & Peckham (1892). We discuss the implications of our results and recommend future lines of work that include DNA barcoding, monitoring of population and community dynamics, plus linkage of climatic data from the newly-installed meteorological station on the Island.

## Introduction

Species diversity, abundance and distribution of biota are universal components of national biodiversity inventories. In conjunction with identifying species, there is also a need to monitor them temporally and spatially. The purpose of monitoring focuses on measuring the condition, status and change of biological diversity to provide information for management actions and achieve conservation outcomes, while improving our fundamental understanding of ecosystems (Lee et al. 2005). Primary data such as faunal inventories provide critical steps in the decision-making process for natural resource management agencies (Silveira et al. 2010). Failure to collect this type of information can lead to disastrous consequences for the protection of species and the environment. Currently, a combination of rapid inventories and long-term ecological research is considered an appropriate method for monitoring studies (Magnusson 2005).

One of the useful groups for such inventory studies are the arachnids, where we refer to their total diversity as the arachnofauna. Within these, the spiders (i.e. the araneofauna) are a particularly important subgroup due to their high taxonomic diversity, abundance and their predatory role within terrestrial ecosystems. Spiders often play an important function in both natural and human modified ecosystems, such as controlling pest populations in agriculture or vectors of concern for public health (Nyffeler and Sunderland 2003). Besides, some spiders are considered pioneer organisms in the colonisation of newly-formed habitats, such as islands. Therefore, they stand as suitable model organisms for the study of processes related to colonisation, establishment and dispersal (Baert 2013, Buchholz et al. 2020). A geographic region with a great potential to study spiders is Coiba, the largest island in Central America, which is part of the Coiba National Park found in the Pacific of Panama and which has had relatively little human impact for centuries. An inventory of the Park could lead to the discovery of new species and eventually allow the study of ecological interactions, biogeographical aspects and tests of evolutionary hypotheses. It is also important to simply know what exists within the Park's territory, allowing for the establishment of adequate management measures. In general, very few studies have conducted inventories of arthropod groups in the Park (Subías et al. 2004, Nieves-Aldrey et al. 2008, Corro-Chang et al. 2017) or described new arthropod species (Miranda et al. 2013, Bolzern 2014). Considering the endemism that has been reported for different taxa in Coiba National Park, including plants, invertebrates and vertebrates (Ridgely and Gwyne 1992,Olson 2007, Ridgely and Gwyne 1992, González et al. 2010, Ibañez 2011, Guzman and Breedy 2012, Mendez-Carvajal 2012, Miranda et al. 2013, Bolzern 2014, Daniel and Carrión 2015, Costas et al. 2015, Flores et al. 2016, Roubik and De Camargo 2012, Valdecasas 2021), the possibility that new and possibly endemic arachnid species exist in the Park is reasonable. The discovery of new species within the Park may promote the strengthening of the protection measures currently in place. For this reason, we performed the first inventory of the araneofauna from Coiba National Park between August 2021 and August 2023. As a World Heritage Site (UNESCO 2005), it might seem that a listed description of all the organisms found in the Park is a necessity. A detailed knowledge of the Park's araneofauna is essential for the application of methodological tools that can help protect its biodiversity.

## Materials and methods

**Figure 1.**
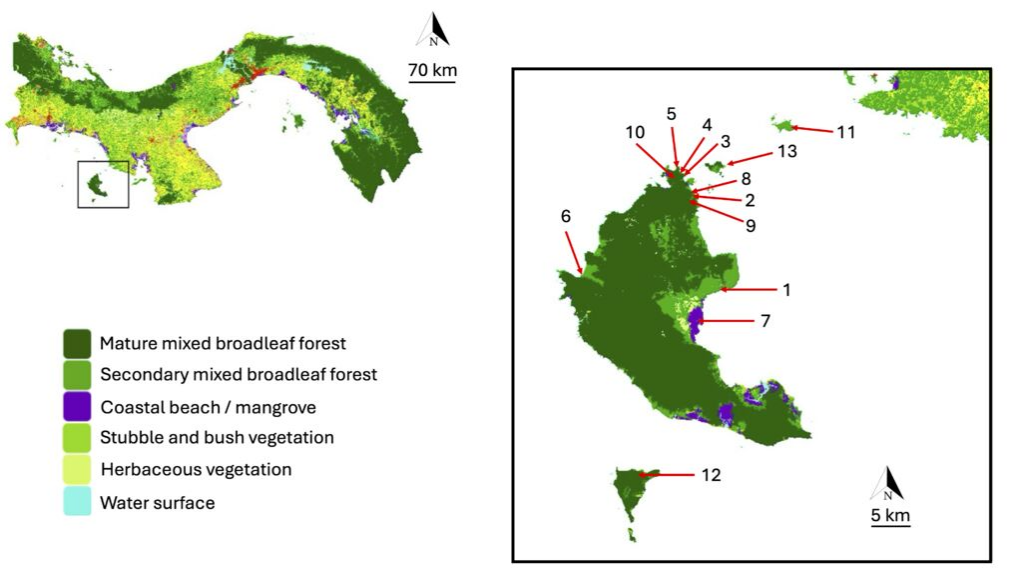
Sampling sites in Coiba National Park depicting the different vegetation types. The square shows the location of Coiba National Park in Panama (left) and a close-up view (right).

Coiba National Park is in the Gulf of Chiriquí (7°29'0.3372"N, 81°47'45.2976"W). The Park contains an area of 2701.25 km^2^ (Fig. 1) and the main island is the largest island in the Central American Pacific, with an area of 503.14 km^2^. In addition, the Park contains 38 island territories, such as Jicarón (20.02 km^2^), Jicarita (1.25 km^2^), Canales de Afuera (2.40 km^2^), Afuerita (0.27 km^2^), Pájaros (0.45 km^2^), Uva (2.57 km^2^), Brincanco (3.30 km^2^), Coibita (2.42 km^2^), along with several smaller islands, islets and rocky promontories (Autoridad Nacional del Ambiente et al. 2009). It is characterised by moderately high temperatures, averaging 25.9ºC. Its climate is humid tropical with precipitation averaging around 3334 mm^3^ and an average relative humidity of over 80% (Poorter 1993, Autoridad Nacional del Ambiente et al. 2009).

This study was carried out between August 2021 and August 2023, when a total of 15 field expeditions were conducted, visiting 13 locations in Coiba and nearby islands (Fig. 1, Table 1). We did not make distinction between 'mature mixed broadleaf forest' and the 'secondary mixed broadleaf forest' (Table 1). The logistical difficulties to travel and stay in the different islands made it difficult to develop a more systematic protocol to monitor elements of population or community dynamics; therefore, our effort focused on creating a basic checklist of species. For the collection of spiders, *ad hoc* active searching was employed involving day (~ 7:00 - 11:00 AM) and night (~ 7:00 PM - 11:00 PM) walks along pre-existing trails and roads. The most visited sites were the locations 2, 3, 4, 5, 8, 9 and 10 where we carried out five visits at each. We performed three visits to sites 11 and 13, two visits to site 6 and 12 and one visit to sites 1 and 7. We inspected trees, cavities, rocks, human-built structures and other elements to maximise collection opportunities. We used entomological beating trays, sweep nets, plus direct tube collection or aspirators to sample spiders at various heights. In addition, we performed a few canopy searches by using equipment for arborism to climb the trees (~ 15 m). We also used pitfall traps to capture wandering and understorey spiders. Ten traps were placed only in three trails in Coiba (sites 8, 9 and 10), approximately 10 m apart. The traps consisted of transparent plastic containers (diameter: 11 cm, height: 8 cm), containing a 400 ml solution of 70% ethanol and soapy water in a 2:1 ratio. We recovered the traps after 5 days per site visited and samples were transfered to 70% ethanol. Overall, collections from all methods were merged as combined records.

The samples were preserved in 70% ethanol and are stored in the laboratory of Dr Dumas Gálvez at the Central American Master Program in Entomology at the University of Panama. All samples are available for future study on request. We carried out identifications by means of published guides in scientific articles, books, websites and, in a few instances, by direct communication with specialists of different groups (Suppl. material 1). Spider families were classified according to foraging patterns in their natural habitat (Cardoso et al. 2011, Rose 2022). The definition of a guild is based on the ecological characteristics of the species (or higher taxa) that determine the exchange of resources. As largely generalist predators of arthropods, the most important resource for spiders is their arthropod prey and their most important ecological characteristics are probably their feeding method, the variety of prey they hunt, vertical stratification, circadian activity, size body and phenology (Uetz et al. 1999, Cardoso et al. 2011). Finally, we built the rarefaction curve in R (R Core Team 2024) with the function iNEXT and ggiNEX in the package iNEXT (Hsieh et al. 2016).

## Checklists

### Preliminary Checklist of Spiders from Coiba National Park

#### Macrophyes elongata

Chickering, 1937

1DAC95B7-5F80-57E6-9139-B1AABCC54536

##### Materials

- **Type status:** Other material. **Occurrence:** recordedBy: Daniel Murcia & Dumas Galvez; sex: 1 male; occurrenceID: 03F01326-7073-52A3-AF2E-7D164175FBDE; **Location:** country: Panama; locality: Coiba; verbatimLocality: Playa Hermosa; verbatimCoordinates: 7° 30' 53.708''N 81° 52' 0.411''O; **Event:** eventDate: 25-03-22

##### Distribution

Costa Rica, Panama

##### Notes

CR-PA

#### Wulfila modestus

Chickering, 1937

04667064-8492-5AFD-8CD1-9ED8917E6332

##### Materials

- **Type status:** Other material. **Occurrence:** recordedBy: Daniel Murcia & Dumas Galvez; sex: 1 male; occurrenceID: 40F8F747-31CE-5CDC-8604-D1736B7A3672; **Location:** country: Panama; locality: Coiba; verbatimLocality: Sendero de Coiba AIP; verbatimCoordinates: 7° 36' 4.903''N 81° 43' 29.06''O; **Event:** eventDate: 24-03-22

##### Distribution

Panama (endemic)

##### Notes

PA

#### Wulfila sp. 1

D22256D7-026C-538F-876A-57963A91DA30

##### Materials

- **Type status:** Other material. **Occurrence:** recordedBy: Daniel Murcia & Dumas Galvez; sex: 1 female; occurrenceID: 170DB6A6-831D-5A72-B028-372ECD5AE121; **Location:** country: Panama; locality: Coiba; verbatimLocality: Sendero Los Monos; verbatimCoordinates: 7° 36' 2.891''N 81° 43' 35.187''O; **Event:** eventDate: 8-02-23

#### Acacesia tenella

(L. Koch, 1871)

1BAC7719-630D-58AD-B34D-D5FD3ADD96EF

##### Materials

- **Type status:** Other material. **Occurrence:** recordedBy: Daniel Murcia & Dumas Galvez; sex: 2 immature; occurrenceID: FEAD34F4-0042-513F-B1FC-89E4E4289611; **Location:** country: Panama; locality: Coiba; verbatimLocality: Playa Hermosa; verbatimCoordinates: 7° 30' 53.708''N 81° 52' 0.411''O; **Event:** eventDate: 26-03-22
- **Type status:** Other material. **Occurrence:** recordedBy: Daniel Murcia & Dumas Galvez; sex: 2 immature; occurrenceID: 6C100287-1414-5656-AA0B-F8350200DA4D; **Location:** country: Panama; locality: Coiba; verbatimLocality: Sendero Santa Cruz; verbatimCoordinates: 7° 37' 32.47''N 81° 43' 51.632''O; **Event:** eventDate: 28-08-21

##### Distribution

Mexico to Brazil, French Guiana, Guyana

##### Notes

MX-SA

#### Allocyclosa bifurca

(McCook, 1887)

F287AA47-94FD-59D1-B3EC-2FBC1581D04A

##### Materials

- **Type status:** Other material. **Occurrence:** recordedBy: Daniel Murcia & Dumas Galvez; sex: 1 female; occurrenceID: 997DE7C2-FCC6-5E9A-9914-332D7BFC9969; **Location:** country: Panama; locality: Coiba; verbatimLocality: Isla Canales Afuera; verbatimCoordinates: 7° 41' 15.77''N 81° 37' 47.539''O; **Event:** eventDate: 27-08-21

##### Distribution

USA to Panama, Turks & Caicos, Cuba, Hispaniola

##### Notes

NA-CA-C

#### Cyclosa caroli

(Hentz, 1850)

3B8CF9D3-191F-5950-9A0B-4149E4017D91

##### Materials

- **Type status:** Other material. **Occurrence:** recordedBy: Daniel Murcia & Dumas Galvez; sex: 1 immature; occurrenceID: E1F142F1-97E3-5539-81CD-04FABF8CD4E5; **Location:** country: Panama; locality: Coiba; verbatimLocality: Est. Coiba AIP Principal; verbatimCoordinates: 7° 36' 0.461''N 81° 43' 27.094''O; **Event:** eventDate: 25-08-21
- **Type status:** Other material. **Occurrence:** recordedBy: Daniel Murcia & Dumas Galvez; sex: 1 female; occurrenceID: 826F6CFA-8CF4-5A9B-8F9F-6340FA0DCA7A; **Location:** country: Panama; locality: Coiba; verbatimLocality: Playa Hermosa; verbatimCoordinates: 7° 30' 53.708''N 81° 52' 0.411''O; **Event:** eventDate: 26-03-22
- **Type status:** Other material. **Occurrence:** recordedBy: Daniel Murcia & Dumas Galvez; sex: 1 female; occurrenceID: 5E1FAD55-6394-5DF1-A5ED-B2C5ED98236F; **Location:** country: Panama; locality: Coiba; verbatimLocality: Sendero de Coiba AIP; verbatimCoordinates: 7° 36' 4.903''N 81° 43' 29.06''O; **Event:** eventDate: 24-03-22
- **Type status:** Other material. **Occurrence:** recordedBy: Daniel Murcia & Dumas Galvez; sex: 1 female; occurrenceID: B219DAFF-4BE0-5DB5-AE5B-FE94F40C98C9; **Location:** country: Panama; locality: Coiba; verbatimLocality: Sendero Los Monos; verbatimCoordinates: 7° 36' 2.891''N 81° 43' 35.187''O; **Event:** eventDate: 18-10-22
- **Type status:** Other material. **Occurrence:** recordedBy: Daniel Murcia & Dumas Galvez; sex: 1 female; occurrenceID: 67DA7CA9-1BD6-5CA8-98A6-3E3BAD183176; **Location:** country: Panama; locality: Coiba; verbatimLocality: Sendero Los Monos; verbatimCoordinates: 7° 36' 2.891''N 81° 43' 35.187''O; **Event:** eventDate: 19-10-22
- **Type status:** Other material. **Occurrence:** recordedBy: Daniel Murcia & Dumas Galvez; sex: 3 female; occurrenceID: 869A8642-0347-5710-BC09-CBAC31BC6F21; **Location:** country: Panama; locality: Coiba; verbatimLocality: Sendero de Coiba AIP; verbatimCoordinates: 7° 36' 4.903''N 81° 43' 29.06''O; **Event:** eventDate: 6-12-22
- **Type status:** Other material. **Occurrence:** recordedBy: Daniel Murcia & Dumas Galvez; sex: 1 female; occurrenceID: CC8A2839-DA79-5FD6-AE54-2667A5574BCE; **Location:** country: Panama; locality: Coiba; verbatimLocality: Sendero de Coiba AIP; verbatimCoordinates: 7° 36' 4.903''N 81° 43' 29.06''O; **Event:** eventDate: 7-12-22
- **Type status:** Other material. **Occurrence:** recordedBy: Daniel Murcia & Dumas Galvez; sex: 1 female; occurrenceID: 9B58D913-A9B3-5CB6-BFA0-188C2E42B778; **Location:** country: Panama; locality: Coiba; verbatimLocality: Isla Jicaron; verbatimCoordinates: 7° 17' 16.022''N 81° 46' 25.359''O; **Event:** eventDate: 8-12-23

##### Distribution

USA, Caribbean to Bolivia

##### Notes

NA-C-SA

#### Eriophora ravilla

(C. L. Koch, 1844)

EA892DAF-2216-580C-B9F3-8F632CCF235F

##### Materials

- **Type status:** Other material. **Occurrence:** recordedBy: Daniel Murcia & Dumas Galvez; sex: 1 immature; occurrenceID: F7780952-23EA-5C73-8F62-02CF6AB9F41D; **Location:** country: Panama; locality: Coiba; verbatimLocality: Playa Hermosa; verbatimCoordinates: 7° 30' 53.708''N 81° 52' 0.411''O; **Event:** eventDate: 1-07-22
- **Type status:** Other material. **Occurrence:** recordedBy: Daniel Murcia & Dumas Galvez; sex: 1 female; occurrenceID: 6054E5B7-2F11-5D0F-9A27-F3EB958FF7A9; **Location:** country: Panama; locality: Coiba; verbatimLocality: Mirador Alto; verbatimCoordinates: 7° 37' 33.488''N 81° 43' 41.199''O; **Event:** eventDate: 26-08-21

##### Distribution

USA to Brazil

##### Notes

NA-SA

#### Eustala bifida

F. O. Pickard-Cambridge, 1904

8EFCF0A9-544D-5FA3-9A46-EAD441D09FD8

##### Materials

- **Type status:** Other material. **Occurrence:** recordedBy: Daniel Murcia & Dumas Galvez; sex: 1 female, 1 male; occurrenceID: A5FD6965-5140-592F-A1CB-B2AD569E64FC; **Location:** country: Panama; locality: Coiba; verbatimLocality: Sendero de Coiba AIP; verbatimCoordinates: 7° 36' 4.903''N 81° 43' 29.06''O; **Event:** eventDate: 6-12-22
- **Type status:** Other material. **Occurrence:** recordedBy: Daniel Murcia & Dumas Galvez; sex: 1 male; occurrenceID: E9DB39CD-F5C8-5793-AD69-F670261787AE; **Location:** country: Panama; locality: Coiba; verbatimLocality: Isla Jicaron; verbatimCoordinates: 7° 17' 16.022''N 81° 46' 25.359''O; **Event:** eventDate: 8-12-23

##### Distribution

USA to Panama

##### Notes

NA-CA

#### Eustala aff. devia

C13FC2F1-70C4-5A2D-BBD9-0FF75B968192

##### Materials

- **Type status:** Other material. **Occurrence:** recordedBy: Daniel Murcia & Dumas Galvez; sex: 1 female; occurrenceID: 09D0E68D-F592-58D7-99F7-FE0A79E17AA8; **Location:** country: Panama; locality: Coiba; verbatimLocality: Playa Hermosa; verbatimCoordinates: 7° 30' 53.708''N 81° 52' 0.411''O; **Event:** eventDate: 1-07-22
- **Type status:** Other material. **Occurrence:** recordedBy: Daniel Murcia & Dumas Galvez; sex: 1 female; occurrenceID: A4EFCD03-72D3-5E39-BD4A-F1EC79D31C88; **Location:** country: Panama; locality: Coiba; verbatimLocality: Playa Hermosa; verbatimCoordinates: 7° 30' 53.708''N 81° 52' 0.411''O; **Event:** eventDate: 25-03-22
- **Type status:** Other material. **Occurrence:** recordedBy: Daniel Murcia & Dumas Galvez; sex: 2 female; occurrenceID: CB83AD3E-30C9-5A7C-8A77-DB9F0A5CC2FC; **Location:** country: Panama; locality: Coiba; verbatimLocality: Playa Hermosa; verbatimCoordinates: 7° 30' 53.708''N 81° 52' 0.411''O; **Event:** eventDate: 26-03-22
- **Type status:** Other material. **Occurrence:** recordedBy: Daniel Murcia & Dumas Galvez; sex: 1 female; occurrenceID: 7B5281E7-6E1E-5297-86B3-73659EF3D082; **Location:** country: Panama; locality: Coiba; verbatimLocality: Isla Jicaron; verbatimCoordinates: 7° 17' 16.022''N 81° 46' 25.359''O; **Event:** eventDate: 8-12-23

#### Eustala exigua

Chickering, 1955

985AF4FD-EA2A-5544-92F6-3EE0EC130B60

##### Materials

- **Type status:** Other material. **Occurrence:** recordedBy: Daniel Murcia & Dumas Galvez; sex: 1 male; occurrenceID: 66AD0A1F-5AB0-56F2-AFD9-B1946F6E7517; **Location:** country: Panama; locality: Coiba; verbatimLocality: Playa Hermosa; verbatimCoordinates: 7° 30' 53.708''N 81° 52' 0.411''O; **Event:** eventDate: 25-03-22

##### Distribution

Panama (endemic)

##### Notes

PA

#### Eustala fuscovittata

(Keyserling, 1864)

41553AF3-AD52-58ED-8BEB-09EABC3337C0

##### Materials

- **Type status:** Other material. **Occurrence:** recordedBy: Daniel Murcia & Dumas Galvez; sex: 1 female, 1 male; occurrenceID: 3F807425-FF9D-5642-9F62-2120B6B6524D; **Location:** country: Panama; locality: Coiba; verbatimLocality: Sendero Los Monos; verbatimCoordinates: 7° 36' 2.891''N 81° 43' 35.187''O; **Event:** eventDate: 8-12-22

##### Distribution

Mexico, Cuba to South America

##### Notes

MX-C-SA

#### Eustala guttata

F. O. Pickard-Cambridge, 1904

9C9CB8A5-5FFA-53E5-A180-6960B25E78E2

##### Materials

- **Type status:** Other material. **Occurrence:** recordedBy: Daniel Murcia & Dumas Galvez; sex: 1 male; occurrenceID: D367EFD4-6764-5A7C-855B-89E5B5C6C78C; **Location:** country: Panama; locality: Coiba; verbatimLocality: Antigua Carcel Principal; verbatimCoordinates: 7° 30' 25.049''N 81° 42' 5.065''O; **Event:** eventDate: 25-08-21
- **Type status:** Other material. **Occurrence:** recordedBy: Daniel Murcia & Dumas Galvez; sex: 1 male; occurrenceID: C97B22DA-6949-52A7-BF5C-8DCE2843C126; **Location:** country: Panama; locality: Coiba; verbatimLocality: Est. MiAmbiente Principal; verbatimCoordinates: 7° 37' 37.024''N 81° 43' 46.56''O; **Event:** eventDate: 25-08-21
- **Type status:** Other material. **Occurrence:** recordedBy: Daniel Murcia & Dumas Galvez; sex: 1 female; occurrenceID: 3F70BC4A-2550-520E-906B-B3D5BF6283A7; **Location:** country: Panama; locality: Coiba; verbatimLocality: Est. MiAmbiente Principal; verbatimCoordinates: 7° 37' 37.024''N 81° 43' 46.56''O; **Event:** eventDate: 29-01-22
- **Type status:** Other material. **Occurrence:** recordedBy: Daniel Murcia & Dumas Galvez; sex: 1 female; occurrenceID: 742165A1-90B2-5079-8475-F68A4914E660; **Location:** country: Panama; locality: Coiba; verbatimLocality: Isla Rancheria; verbatimCoordinates: 7° 38' 14.867''N 81° 42' 10.497''O; **Event:** eventDate: 24-03-22
- **Type status:** Other material. **Occurrence:** recordedBy: Daniel Murcia & Dumas Galvez; sex: 1 female; occurrenceID: FF2E86E4-BFF2-5A47-9CD0-04047C5615B8; **Location:** country: Panama; locality: Coiba; verbatimLocality: Mirador Gambute; verbatimCoordinates: 7° 37' 41.657''N 81° 43' 59.174''O; **Event:** eventDate: 27-08-21
- **Type status:** Other material. **Occurrence:** recordedBy: Daniel Murcia & Dumas Galvez; sex: 1 female; occurrenceID: 0E92BD83-8810-57EC-BB15-4BFC2BBF54F1; **Location:** country: Panama; locality: Coiba; verbatimLocality: Playa Hermosa; verbatimCoordinates: 7° 30' 53.708''N 81° 52' 0.411''O; **Event:** eventDate: 26-03-22
- **Type status:** Other material. **Occurrence:** recordedBy: Daniel Murcia & Dumas Galvez; sex: 1 male; occurrenceID: 4657EF7A-6DD1-5FC8-A1D9-B7204B799990; **Location:** country: Panama; locality: Coiba; verbatimLocality: Sendero de Coiba AIP; verbatimCoordinates: 7° 36' 4.903''N 81° 43' 29.06''O; **Event:** eventDate: 24-03-22
- **Type status:** Other material. **Occurrence:** recordedBy: Daniel Murcia & Dumas Galvez; sex: 1 female, 1 male; occurrenceID: FB1869D1-83ED-5775-8DFB-443931099A19; **Location:** country: Panama; locality: Coiba; verbatimLocality: Sendero Los Monos; verbatimCoordinates: 7° 36' 2.891''N 81° 43' 35.187''O; **Event:** eventDate: 8-12-22

##### Distribution

Mexico to Brazil

##### Notes

MX-SA

#### Eustala scutigera

(O. Pickard-Cambridge, 1898)

B723AB41-0B39-5081-9A53-4AA7CADA1957

##### Materials

- **Type status:** Other material. **Occurrence:** recordedBy: Daniel Murcia & Dumas Galvez; sex: 1 female; occurrenceID: 8AB8AF76-2813-5330-9C01-D16487D14E24; **Location:** country: Panama; locality: Coiba; verbatimLocality: Isla Rancheria; verbatimCoordinates: 7° 38' 14.867''N 81° 42' 10.497''O; **Event:** eventDate: 24-03-22
- **Type status:** Other material. **Occurrence:** recordedBy: Daniel Murcia & Dumas Galvez; sex: 1 female; occurrenceID: 30413064-0B62-508F-BB0E-F2A732F25F03; **Location:** country: Panama; locality: Coiba; verbatimLocality: Sendero de Coiba AIP; verbatimCoordinates: 7° 36' 4.903''N 81° 43' 29.06''O; **Event:** eventDate: 24-03-22
- **Type status:** Other material. **Occurrence:** recordedBy: Daniel Murcia & Dumas Galvez; sex: 1 male; occurrenceID: 5D476A87-7F0D-502B-B51C-D58D517583FF; **Location:** country: Panama; locality: Coiba; verbatimLocality: Sendero Los Monos; verbatimCoordinates: 7° 36' 2.891''N 81° 43' 35.187''O; **Event:** eventDate: 18-10-22
- **Type status:** Other material. **Occurrence:** recordedBy: Daniel Murcia & Dumas Galvez; sex: 2 female; occurrenceID: C00789D3-2E5B-590D-963E-19D4097D5B9B; **Location:** country: Panama; locality: Coiba; verbatimLocality: Sendero Los Monos; verbatimCoordinates: 7° 36' 2.891''N 81° 43' 35.187''O; **Event:** eventDate: 8-12-22
- **Type status:** Other material. **Occurrence:** recordedBy: Daniel Murcia & Dumas Galvez; sex: 2 female, 2 male; occurrenceID: 773B8662-919C-5BF2-B84B-AC5895102749; **Location:** country: Panama; locality: Coiba; verbatimLocality: Sendero Los Monos; verbatimCoordinates: 7° 36' 2.891''N 81° 43' 35.187''O; **Event:** eventDate: 7-02-23
- **Type status:** Other material. **Occurrence:** recordedBy: Daniel Murcia & Dumas Galvez; sex: 1 female, 1 male; occurrenceID: B61C25EB-AFFF-5728-8E99-9CE7ACF6EE93; **Location:** country: Panama; locality: Coiba; verbatimLocality: Isla Jicaron; verbatimCoordinates: 7° 17' 16.022''N 81° 46' 25.359''O; **Event:** eventDate: 8-12-23

##### Distribution

Mexico to Panama

##### Notes

MX-CA

#### Eustala semifoliata

(O. Pickard-Cambridge, 1899)

032D17BD-77C8-500F-95E6-ED33B5D0846B

##### Materials

- **Type status:** Other material. **Occurrence:** recordedBy: Daniel Murcia & Dumas Galvez; sex: 1 female; occurrenceID: E9344976-25A8-5BEB-A713-DEFA8290F296; **Location:** country: Panama; locality: Coiba; verbatimLocality: Isla Canales Afuera; verbatimCoordinates: 7° 41' 15.77''N 81° 37' 47.539''O; **Event:** eventDate: 30-01-22
- **Type status:** Other material. **Occurrence:** recordedBy: Daniel Murcia & Dumas Galvez; sex: 1 female; occurrenceID: B5C1543C-59AE-5A36-AA5B-B2532EDD39FF; **Location:** country: Panama; locality: Coiba; verbatimLocality: Mirador Alto; verbatimCoordinates: 7° 37' 33.488''N 81° 43' 41.199''O; **Event:** eventDate: 26-08-21
- **Type status:** Other material. **Occurrence:** recordedBy: Daniel Murcia & Dumas Galvez; sex: 1 immature; occurrenceID: 8D32995F-7D47-5751-BD29-3314A6A36B18; **Location:** country: Panama; locality: Coiba; verbatimLocality: Sendero Los Monos; verbatimCoordinates: 7° 36' 2.891''N 81° 43' 35.187''O; **Event:** eventDate: 29-01-22

##### Distribution

Central America

##### Notes

CA

#### Eustala sp. 1

6C64D789-8ADA-59EA-A875-88B2CA8FE854

##### Materials

- **Type status:** Other material. **Occurrence:** recordedBy: Daniel Murcia & Dumas Galvez; sex: 1 male; occurrenceID: 95772F64-C296-52EE-93E1-8B0520FDE2E3; **Location:** country: Panama; locality: Coiba; verbatimLocality: Sendero de Coiba AIP; verbatimCoordinates: 7° 36' 4.903''N 81° 43' 29.06''O; **Event:** eventDate: 6-12-22

#### Eustala sp. 2

A3B18E6E-8129-5CB4-A905-13EC9CC10A8A

##### Materials

- **Type status:** Other material. **Occurrence:** recordedBy: Daniel Murcia & Dumas Galvez; sex: 1 female; occurrenceID: A9FE1CCB-EB16-5816-A1E9-E3C606D472DE; **Location:** country: Panama; locality: Coiba; verbatimLocality: San Juan; verbatimCoordinates: 7° 27' 34.902''N 81° 43' 18.613''O; **Event:** eventDate: 26-08-21

#### Eustala sp. 3

32369A8B-5F18-5258-86C4-96AAD5B74AF6

##### Materials

- **Type status:** Other material. **Occurrence:** recordedBy: Daniel Murcia & Dumas Galvez; sex: 1 immature; occurrenceID: 8C19DF9D-3579-50DE-9294-7A49F149D5BB; **Location:** country: Panama; locality: Coiba; verbatimLocality: Est. MiAmbiente Principal; verbatimCoordinates: 7° 37' 37.024''N 81° 43' 46.56''O; **Event:** eventDate: 16-09-21
- **Type status:** Other material. **Occurrence:** recordedBy: Daniel Murcia & Dumas Galvez; sex: 1 immature; occurrenceID: 25B85CF1-7A19-5093-982A-EBD98DE674BD; **Location:** country: Panama; locality: Coiba; verbatimLocality: Est. MiAmbiente Principal; verbatimCoordinates: 7° 37' 37.024''N 81° 43' 46.56''O; **Event:** eventDate: 25-08-21
- **Type status:** Other material. **Occurrence:** recordedBy: Daniel Murcia & Dumas Galvez; sex: 2 immature; occurrenceID: 02D2DDC7-EAC1-5D5F-AA8A-71B2C10ADADC; **Location:** country: Panama; locality: Coiba; verbatimLocality: Est. MiAmbiente Principal; verbatimCoordinates: 7° 37' 37.024''N 81° 43' 46.56''O; **Event:** eventDate: 29-01-22
- **Type status:** Other material. **Occurrence:** recordedBy: Daniel Murcia & Dumas Galvez; sex: 1 immature; occurrenceID: 0BF4EEE4-480D-5260-B197-B621DB030787; **Location:** country: Panama; locality: Coiba; verbatimLocality: Mirador Alto; verbatimCoordinates: 7° 37' 33.488''N 81° 43' 41.199''O; **Event:** eventDate: 26-08-21
- **Type status:** Other material. **Occurrence:** recordedBy: Daniel Murcia & Dumas Galvez; sex: 3 immature; occurrenceID: 07E94C98-7147-54DE-BD5D-1B6A09ABCC34; **Location:** country: Panama; locality: Coiba; verbatimLocality: Playa Hermosa; verbatimCoordinates: 7° 30' 53.708''N 81° 52' 0.411''O; **Event:** eventDate: 25-03-22
- **Type status:** Other material. **Occurrence:** recordedBy: Daniel Murcia & Dumas Galvez; sex: 4 immature; occurrenceID: 59BD1546-B792-57F4-B30F-D5BACFC547FA; **Location:** country: Panama; locality: Coiba; verbatimLocality: Playa Hermosa; verbatimCoordinates: 7° 30' 53.708''N 81° 52' 0.411''O; **Event:** eventDate: 26-03-22
- **Type status:** Other material. **Occurrence:** recordedBy: Daniel Murcia & Dumas Galvez; sex: 1 immature; occurrenceID: 3EF9CCF9-EA47-5094-847C-BE5DF676870C; **Location:** country: Panama; locality: Coiba; verbatimLocality: San Juan; verbatimCoordinates: 7° 27' 34.902''N 81° 43' 18.613''O; **Event:** eventDate: 26-08-21
- **Type status:** Other material. **Occurrence:** recordedBy: Daniel Murcia & Dumas Galvez; sex: 2 immature; occurrenceID: 3810C174-0972-53ED-8ADF-4DF64721DE51; **Location:** country: Panama; locality: Coiba; verbatimLocality: Sendero de Coiba AIP; verbatimCoordinates: 7° 36' 4.903''N 81° 43' 29.06''O; **Event:** eventDate: 24-03-22
- **Type status:** Other material. **Occurrence:** recordedBy: Daniel Murcia & Dumas Galvez; sex: 2 immature; occurrenceID: 3D355049-0B90-5692-B259-DBBFCDDC51DF; **Location:** country: Panama; locality: Coiba; verbatimLocality: Sendero Los Monos; verbatimCoordinates: 7° 36' 2.891''N 81° 43' 35.187''O; **Event:** eventDate: 18-10-22
- **Type status:** Other material. **Occurrence:** recordedBy: Daniel Murcia & Dumas Galvez; sex: 5 immature; occurrenceID: A3316CEF-B2AF-5192-8647-0BA8ECC5C4F2; **Location:** country: Panama; locality: Coiba; verbatimLocality: Sendero Los Monos; verbatimCoordinates: 7° 36' 2.891''N 81° 43' 35.187''O; **Event:** eventDate: 29-01-22
- **Type status:** Other material. **Occurrence:** recordedBy: Daniel Murcia & Dumas Galvez; sex: 1 immature; occurrenceID: 871D0850-4DEF-5387-BB48-D2D0C5A87448; **Location:** country: Panama; locality: Coiba; verbatimLocality: Sendero Santa Cruz; verbatimCoordinates: 7° 37' 32.47''N 81° 43' 51.632''O; **Event:** eventDate: 28-08-21
- **Type status:** Other material. **Occurrence:** recordedBy: Daniel Murcia & Dumas Galvez; sex: 5 immature; occurrenceID: 02F53810-24D7-5407-9672-703A337F3C70; **Location:** country: Panama; locality: Coiba; verbatimLocality: Sendero de Coiba AIP; verbatimCoordinates: 7° 36' 4.903''N 81° 43' 29.06''O; **Event:** eventDate: 7-12-22
- **Type status:** Other material. **Occurrence:** recordedBy: Daniel Murcia & Dumas Galvez; sex: 2 immature; occurrenceID: 4F31E447-04B2-5D88-9930-0EEFC348B821; **Location:** country: Panama; locality: Coiba; verbatimLocality: Sendero Los Monos; verbatimCoordinates: 7° 36' 2.891''N 81° 43' 35.187''O; **Event:** eventDate: 8-12-22
- **Type status:** Other material. **Occurrence:** recordedBy: Daniel Murcia & Dumas Galvez; sex: 5 immature; occurrenceID: 51F2E028-437A-5724-9F0E-C11774A0616C; **Location:** country: Panama; locality: Coiba; verbatimLocality: Sendero Los Monos; verbatimCoordinates: 7° 36' 2.891''N 81° 43' 35.187''O; **Event:** eventDate: 7-02-23
- **Type status:** Other material. **Occurrence:** recordedBy: Daniel Murcia & Dumas Galvez; sex: 24 immature; occurrenceID: 3213BB44-BD03-5483-8A46-A4EA63BF3C90; **Location:** country: Panama; locality: Coiba; verbatimLocality: Isla Jicaron; verbatimCoordinates: 7° 17' 16.022''N 81° 46' 25.359''O; **Event:** eventDate: 8-12-23

#### Eustala tantula

Chickering, 1955

B55E4FBA-D6B6-563A-9A20-879622CD7751

##### Materials

- **Type status:** Other material. **Occurrence:** recordedBy: Daniel Murcia & Dumas Galvez; sex: 1 male; occurrenceID: 8FBB6CA9-746C-5037-AB0F-45C6FF291B40; **Location:** country: Panama; locality: Coiba; verbatimLocality: Sendero Santa Cruz; verbatimCoordinates: 7° 37' 32.47''N 81° 43' 51.632''O; **Event:** eventDate: 28-08-21

##### Distribution

Panama (endemic)

##### Notes

PA

#### Larinia directa

(Hentz, 1847)

00F02AD0-C910-5F6F-8B75-046859DE8685

##### Materials

- **Type status:** Other material. **Occurrence:** recordedBy: Daniel Murcia & Dumas Galvez; sex: 1 female; occurrenceID: B4E687B8-1F8D-503B-AD8B-1C717D485653; **Location:** country: Panama; locality: Coiba; verbatimLocality: San Juan; verbatimCoordinates: 7° 27' 34.902''N 81° 43' 18.613''O; **Event:** eventDate: 26-08-21
- **Type status:** Other material. **Occurrence:** recordedBy: Daniel Murcia & Dumas Galvez; sex: 2 immature; occurrenceID: 89541C46-45E7-5235-86E1-B84E108DFB52; **Location:** country: Panama; locality: Coiba; verbatimLocality: Sendero Los Monos; verbatimCoordinates: 7° 36' 2.891''N 81° 43' 35.187''O; **Event:** eventDate: 18-10-22

##### Distribution

USA to Brazil

##### Notes

NA-SA

#### Metazygia keyserlingi

Banks, 1929

F4DB1CB1-F523-5F39-A93B-F3DF29C9DD51

##### Materials

- **Type status:** Other material. **Occurrence:** recordedBy: Daniel Murcia & Dumas Galvez; sex: 2 female; occurrenceID: 413F6265-4E5A-5BFC-A5C6-529A66A137B9; **Location:** country: Panama; locality: Coiba; verbatimLocality: Sendero Los Monos; verbatimCoordinates: 7° 36' 2.891''N 81° 43' 35.187''O; **Event:** eventDate: 7-02-23
- **Type status:** Other material. **Occurrence:** recordedBy: Daniel Murcia & Dumas Galvez; sex: 2 female, 1 male; occurrenceID: 42B4F253-9EDB-57B2-A6EA-4F7D997F19C8; **Location:** country: Panama; locality: Coiba; verbatimLocality: Isla Jicaron; verbatimCoordinates: 7° 17' 16.022''N 81° 46' 25.359''O; **Event:** eventDate: 8-12-23

##### Distribution

Costa Rica, Panama, Colombia, Trinidad

##### Notes

CA-SA

#### Micrathena horrida

(Taczanowski, 1873)

61BB9801-27DA-5EBB-855E-C46FEBF380B7

##### Materials

- **Type status:** Other material. **Occurrence:** recordedBy: Daniel Murcia & Dumas Galvez; sex: 1 female; occurrenceID: 98A86C07-C748-5212-A4EE-8DE5B2E1B672; **Location:** country: Panama; locality: Coiba; verbatimLocality: Isla Jicaron; verbatimCoordinates: 7° 17' 16.022''N 81° 46' 25.359''O; **Event:** eventDate: 8-12-23

##### Distribution

Greater Antilles, Mexico to Argentina

##### Notes

MX-C-SA

#### Parawixia hypocrita

(O. Pickard-Cambridge, 1889)

BB17AEBF-2EE3-5F0B-8480-D79F173689E6

##### Materials

- **Type status:** Other material. **Occurrence:** recordedBy: Daniel Murcia & Dumas Galvez; sex: 1 immature; occurrenceID: 7ADAE52D-007E-504C-B271-8ED7E97824E2; **Location:** country: Panama; locality: Coiba; verbatimLocality: Playa Hermosa; verbatimCoordinates: 7° 30' 53.708''N 81° 52' 0.411''O; **Event:** eventDate: 1-07-22
- **Type status:** Other material. **Occurrence:** recordedBy: Daniel Murcia & Dumas Galvez; sex: 1 immature; occurrenceID: 9DFF06D6-F717-5BAA-8C60-2F9B7C7FE6BF; **Location:** country: Panama; locality: Coiba; verbatimLocality: Sendero de Coiba AIP; verbatimCoordinates: 7° 36' 4.903''N 81° 43' 29.06''O; **Event:** eventDate: 6-12-22
- **Type status:** Other material. **Occurrence:** recordedBy: Daniel Murcia & Dumas Galvez; sex: 1 immature; occurrenceID: 2773A0E7-3174-5BD6-AE8C-8779F339B02F; **Location:** country: Panama; locality: Coiba; verbatimLocality: Sendero Los Monos; verbatimCoordinates: 7° 36' 2.891''N 81° 43' 35.187''O; **Event:** eventDate: 18-10-22
- **Type status:** Other material. **Occurrence:** recordedBy: Daniel Murcia & Dumas Galvez; sex: 3 immature; occurrenceID: 184E5CD0-0C4D-559A-A5E1-41FA5734E6F5; **Location:** country: Panama; locality: Coiba; verbatimLocality: Sendero de Coiba AIP; verbatimCoordinates: 7° 36' 4.903''N 81° 43' 29.06''O; **Event:** eventDate: 7-12-22
- **Type status:** Other material. **Occurrence:** recordedBy: Daniel Murcia & Dumas Galvez; sex: 1 immature; occurrenceID: 52D324C7-5FC9-57CC-9F0B-C3E2B96D4E11; **Location:** country: Panama; locality: Coiba; verbatimLocality: Sendero Los Monos; verbatimCoordinates: 7° 36' 2.891''N 81° 43' 35.187''O; **Event:** eventDate: 8-12-22
- **Type status:** Other material. **Occurrence:** recordedBy: Daniel Murcia & Dumas Galvez; sex: 1 female, 2 immature; occurrenceID: 44EF82AC-F926-5A75-980E-A0F8C3C011B0; **Location:** country: Panama; locality: Coiba; verbatimLocality: Isla Jicaron; verbatimCoordinates: 7° 17' 16.022''N 81° 46' 25.359''O; **Event:** eventDate: 8-12-23

##### Distribution

Guatemala to Brazil

##### Notes

CA-SA

#### Parawixia sp. 1

14C080EB-26BE-5104-B39B-B75337042E65

##### Materials

- **Type status:** Other material. **Occurrence:** recordedBy: Daniel Murcia & Dumas Galvez; sex: 1 male; occurrenceID: 8FF5CACA-7681-5C12-8802-59249A6CBBE3; **Location:** country: Panama; locality: Coiba; verbatimLocality: Sendero Los Monos; verbatimCoordinates: 7° 36' 2.891''N 81° 43' 35.187''O; **Event:** eventDate: 7-02-23

#### Pronous intus

Levi, 1995

795C79BA-7C67-5574-8416-7A2A98D04822

##### Materials

- **Type status:** Other material. **Occurrence:** recordedBy: Daniel Murcia & Dumas Galvez; sex: 1 immature; occurrenceID: EF2FF8A0-DE0D-5359-9A04-13625B4C1417; **Location:** country: Panama; locality: Coiba; verbatimLocality: Playa Hermosa; verbatimCoordinates: 7° 30' 53.708''N 81° 52' 0.411''O; **Event:** eventDate: 25-03-22
- **Type status:** Other material. **Occurrence:** recordedBy: Daniel Murcia & Dumas Galvez; sex: 1 female; occurrenceID: CC3442DF-D734-53AB-8127-87D97EBAA2BC; **Location:** country: Panama; locality: Coiba; verbatimLocality: Sendero Santa Cruz; verbatimCoordinates: 7° 37' 32.47''N 81° 43' 51.632''O; **Event:** eventDate: 28-08-21

##### Distribution

Costa Rica to Brazil

##### Notes

CA-SA

#### Wagneriana tauricornis

(O. Pickard-Cambridge, 1889)

8C0DC251-664C-5B5B-B0E6-F5D566528652

##### Materials

- **Type status:** Other material. **Occurrence:** recordedBy: Daniel Murcia & Dumas Galvez; sex: 3 female, 1 male; occurrenceID: F958EBA5-D0A6-5F95-AAFB-483DA4B617EB; **Location:** country: Panama; locality: Coiba; verbatimLocality: Playa Hermosa; verbatimCoordinates: 7° 30' 53.708''N 81° 52' 0.411''O; **Event:** eventDate: 1-07-22
- **Type status:** Other material. **Occurrence:** recordedBy: Daniel Murcia & Dumas Galvez; sex: 5 female; occurrenceID: E856E17D-8A19-5512-B8C3-CE1031B7F7B6; **Location:** country: Panama; locality: Coiba; verbatimLocality: Sendero de Coiba AIP; verbatimCoordinates: 7° 36' 4.903''N 81° 43' 29.06''O; **Event:** eventDate: 6-12-22
- **Type status:** Other material. **Occurrence:** recordedBy: Daniel Murcia & Dumas Galvez; sex: 1 female, male; occurrenceID: 26A0095E-7A68-5106-8B51-6913372FCD28; **Location:** country: Panama; locality: Coiba; verbatimLocality: Isla Canales Afuera; verbatimCoordinates: 7° 41' 15.77''N 81° 37' 47.539''O; **Event:** eventDate: 27-08-21
- **Type status:** Other material. **Occurrence:** recordedBy: Daniel Murcia & Dumas Galvez; sex: 1 male, 1 immature; occurrenceID: C59FDC27-3074-5674-B97E-29F86D18CFE8; **Location:** country: Panama; locality: Coiba; verbatimLocality: Isla Canales Afuera; verbatimCoordinates: 7° 41' 15.77''N 81° 37' 47.539''O; **Event:** eventDate: 30-01-22
- **Type status:** Other material. **Occurrence:** recordedBy: Daniel Murcia & Dumas Galvez; sex: 2 immature; occurrenceID: BA067B4C-B292-5325-9938-DBD4A6B5EF28; **Location:** country: Panama; locality: Coiba; verbatimLocality: Isla Rancheria; verbatimCoordinates: 7° 38' 14.867''N 81° 42' 10.497''O; **Event:** eventDate: 24-03-22
- **Type status:** Other material. **Occurrence:** recordedBy: Daniel Murcia & Dumas Galvez; sex: 1 immature; occurrenceID: F684016D-0907-5816-A6F2-A4372CEA5B70; **Location:** country: Panama; locality: Coiba; verbatimLocality: Playa Hermosa; verbatimCoordinates: 7° 30' 53.708''N 81° 52' 0.411''O; **Event:** eventDate: 25-03-22
- **Type status:** Other material. **Occurrence:** recordedBy: Daniel Murcia & Dumas Galvez; sex: 1 female, 1 male, 1 immature; occurrenceID: FA29352F-ABF4-58E1-A2E0-BC073CDD9698; **Location:** country: Panama; locality: Coiba; verbatimLocality: Sendero Los Monos; verbatimCoordinates: 7° 36' 2.891''N 81° 43' 35.187''O; **Event:** eventDate: 19-10-22
- **Type status:** Other material. **Occurrence:** recordedBy: Daniel Murcia & Dumas Galvez; sex: 1 immature; occurrenceID: A2079E5B-1D09-5DC9-BE56-832BCD39FAB4; **Location:** country: Panama; locality: Coiba; verbatimLocality: Sendero Los Monos; verbatimCoordinates: 7° 36' 2.891''N 81° 43' 35.187''O; **Event:** eventDate: 26-08-21
- **Type status:** Other material. **Occurrence:** recordedBy: Daniel Murcia & Dumas Galvez; sex: 1 female; occurrenceID: E743B07E-3A2C-5DB6-A3BE-3D217D66033C; **Location:** country: Panama; locality: Coiba; verbatimLocality: Sendero Los Monos; verbatimCoordinates: 7° 36' 2.891''N 81° 43' 35.187''O; **Event:** eventDate: 29-01-22
- **Type status:** Other material. **Occurrence:** recordedBy: Daniel Murcia & Dumas Galvez; sex: 1 immature; occurrenceID: 7B2FA28F-52CF-54FA-989A-5D68D87FFEB8; **Location:** country: Panama; locality: Coiba; verbatimLocality: Sendero Santa Cruz; verbatimCoordinates: 7° 37' 32.47''N 81° 43' 51.632''O; **Event:** eventDate: 28-08-21
- **Type status:** Other material. **Occurrence:** recordedBy: Daniel Murcia & Dumas Galvez; sex: 3 female, 1 male; occurrenceID: E4101BF7-F7C8-5BC9-8F4C-35D6BF4497F2; **Location:** country: Panama; locality: Coiba; verbatimLocality: Sendero Los Monos; verbatimCoordinates: 7° 36' 2.891''N 81° 43' 35.187''O; **Event:** eventDate: 7-02-23
- **Type status:** Other material. **Occurrence:** recordedBy: Daniel Murcia & Dumas Galvez; sex: 6 female; occurrenceID: D2656081-FDD6-5600-9C6F-608BBB335E65; **Location:** country: Panama; locality: Coiba; verbatimLocality: Isla Jicaron; verbatimCoordinates: 7° 17' 16.022''N 81° 46' 25.359''O; **Event:** eventDate: 8-12-23

##### Distribution

USA to Peru

##### Notes

NA-SA

#### Witica crassicaudus

(Keyserling, 1865)

C47B7EB4-6C89-57BA-8EA2-560D36CA6D9E

##### Materials

- **Type status:** Other material. **Occurrence:** recordedBy: Daniel Murcia & Dumas Galvez; sex: 1 female; occurrenceID: 03E47AE0-A1E0-52F5-B77A-E8CF140D79B9; **Location:** country: Panama; locality: Coiba; verbatimLocality: Isla Canales Afuera; verbatimCoordinates: 7° 41' 15.77''N 81° 37' 47.539''O; **Event:** eventDate: 30-01-22
- **Type status:** Other material. **Occurrence:** recordedBy: Daniel Murcia & Dumas Galvez; sex: 1 female; occurrenceID: 7CEC6505-C8FE-5D82-BD7E-5E289C4136B4; **Location:** country: Panama; locality: Coiba; verbatimLocality: Sendero Los Monos; verbatimCoordinates: 7° 36' 2.891''N 81° 43' 35.187''O; **Event:** eventDate: 29-01-22
- **Type status:** Other material. **Occurrence:** recordedBy: Daniel Murcia & Dumas Galvez; sex: 1 immature; occurrenceID: 3DF112EB-642C-55A9-B4AD-EEBA3A7C0216; **Location:** country: Panama; locality: Coiba; verbatimLocality: Sendero Los Monos; verbatimCoordinates: 7° 36' 2.891''N 81° 43' 35.187''O; **Event:** eventDate: 8-12-22
- **Type status:** Other material. **Occurrence:** recordedBy: Daniel Murcia & Dumas Galvez; sex: 1 immature; occurrenceID: B36B6D51-67AD-59B7-B355-AE428D2A209D; **Location:** country: Panama; locality: Coiba; verbatimLocality: Isla Jicaron; verbatimCoordinates: 7° 17' 16.022''N 81° 46' 25.359''O; **Event:** eventDate: 8-12-23

##### Distribution

Mexico to Peru

##### Notes

MX-SA

#### Nops largus

Chickering, 1967

47C9D752-455D-5CCC-B456-2E2C5994828C

##### Materials

- **Type status:** Other material. **Occurrence:** recordedBy: Daniel Murcia & Dumas Galvez; sex: 1 male; occurrenceID: C9ECB771-0843-5AD3-AD74-4220C0AEF0FF; **Location:** country: Panama; locality: Coiba; verbatimLocality: Sendero de Coiba AIP; verbatimCoordinates: 7° 36' 4.903''N 81° 43' 29.06''O; **Event:** eventDate: 24-03-22

##### Distribution

Panama (endemic)

##### Notes

PA

#### Eutichurus putus

O. Pickard-Cambridge, 1898

55442A3C-81D6-5E4B-9B67-AE4065A35368

##### Materials

- **Type status:** Other material. **Occurrence:** recordedBy: Daniel Murcia & Dumas Galvez; sex: 1 female; occurrenceID: CC94598B-2E85-5AB7-95D1-41B10B8223A6; **Location:** country: Panama; locality: Coiba; verbatimLocality: Mirador Alto; verbatimCoordinates: 7° 37' 33.488''N 81° 43' 41.199''O; **Event:** eventDate: 26-08-21

##### Distribution

Panama, Colombia, Ecuador, Peru, Brazil

##### Notes

PA-SA

#### Elaver cf. tigrina

208A2491-7E40-51B1-BDCF-AB2E5595B4A5

##### Materials

- **Type status:** Other material. **Occurrence:** recordedBy: Daniel Murcia & Dumas Galvez; sex: 1 female; occurrenceID: D9A81B02-7EE3-5972-8948-23523B0E249F; **Location:** country: Panama; locality: Coiba; verbatimLocality: Playa Hermosa; verbatimCoordinates: 7° 30' 53.708''N 81° 52' 0.411''O; **Event:** eventDate: 26-03-22
- **Type status:** Other material. **Occurrence:** recordedBy: Daniel Murcia & Dumas Galvez; sex: 1 female; occurrenceID: 34749A51-0661-5FA8-B145-4FFCE501277E; **Location:** country: Panama; locality: Coiba; verbatimLocality: Sendero Los Monos; verbatimCoordinates: 7° 36' 2.891''N 81° 43' 35.187''O; **Event:** eventDate: 8-12-22
- **Type status:** Other material. **Occurrence:** recordedBy: Daniel Murcia & Dumas Galvez; sex: 1 female; occurrenceID: 83882F5D-59D0-57F3-B5CD-F8FF1F2736BB; **Location:** country: Panama; locality: Coiba; verbatimLocality: Sendero Los Monos; verbatimCoordinates: 7° 36' 2.891''N 81° 43' 35.187''O; **Event:** eventDate: 7-02-23
- **Type status:** Other material. **Occurrence:** recordedBy: Daniel Murcia & Dumas Galvez; sex: 1 male; occurrenceID: 91CF84BF-2F62-589E-9E5C-1FDFC0B85CD9; **Location:** country: Panama; locality: Coiba; verbatimLocality: Sendero Los Monos; verbatimCoordinates: 7° 36' 2.891''N 81° 43' 35.187''O; **Event:** eventDate: 8-02-23

#### Elaver lutescens

(Schmidt, 1971)

BA64A406-3EAB-5AED-B822-BAC94FD6A50E

##### Materials

- **Type status:** Other material. **Occurrence:** recordedBy: Daniel Murcia & Dumas Galvez; sex: 1 female; occurrenceID: 784606C4-B558-5A9F-B83C-2336955E5ABE; **Location:** country: Panama; locality: Coiba; verbatimLocality: Sendero de Coiba AIP; verbatimCoordinates: 7° 36' 4.903''N 81° 43' 29.06''O; **Event:** eventDate: 7-12-22

##### Distribution

Panama to Brazil

##### Notes

PA-SA

#### Elaver sp. 1

AE69E2F5-927A-5C8D-9BC5-FF87A8F3F3AE

##### Materials

- **Type status:** Other material. **Occurrence:** recordedBy: Daniel Murcia & Dumas Galvez; sex: 1 female; occurrenceID: 83144A40-7574-593B-AD92-8788252BC825; **Location:** country: Panama; locality: Coiba; verbatimLocality: Sendero Los Monos; verbatimCoordinates: 7° 36' 2.891''N 81° 43' 35.187''O; **Event:** eventDate: 29-01-22

#### Elaver spp.

997CF164-8CD0-5CA0-B128-A66E780680B8

##### Materials

- **Type status:** Other material. **Occurrence:** recordedBy: Daniel Murcia & Dumas Galvez; sex: 1 immature; occurrenceID: 5089C426-7133-5495-BF82-97C229CBCCDD; **Location:** country: Panama; locality: Coiba; verbatimLocality: Sendero de Coiba AIP; verbatimCoordinates: 7° 30' 53.708''N 81° 52' 0.411''O; **Event:** eventDate: 6-12-22
- **Type status:** Other material. **Occurrence:** recordedBy: Daniel Murcia & Dumas Galvez; sex: 1 immature; occurrenceID: C379FCB6-C71D-5909-8BD6-EDDDC2EDB9E8; **Location:** country: Panama; locality: Coiba; verbatimLocality: Isla Rancheria; verbatimCoordinates: 7° 30' 53.708''N 81° 52' 0.411''O; **Event:** eventDate: 24-03-22
- **Type status:** Other material. **Occurrence:** recordedBy: Daniel Murcia & Dumas Galvez; sex: 1 immature; occurrenceID: BC5F176E-B8F9-5936-AA08-9BFCB2B1FE46; **Location:** country: Panama; locality: Coiba; verbatimLocality: Playa Hermosa; verbatimCoordinates: 7° 38' 14.867''N 81° 42' 10.497''O; **Event:** eventDate: 25-03-22
- **Type status:** Other material. **Occurrence:** recordedBy: Daniel Murcia & Dumas Galvez; sex: 1 immature; occurrenceID: D6475F13-B224-51CF-98B8-F1F2C48522AC; **Location:** country: Panama; locality: Coiba; verbatimLocality: Playa Hermosa; verbatimCoordinates: 7° 36' 2.891''N 81° 43' 35.187''O; **Event:** eventDate: 26-03-22
- **Type status:** Other material. **Occurrence:** recordedBy: Daniel Murcia & Dumas Galvez; sex: 1 immature; occurrenceID: CF3B539B-0BB5-5416-926B-6BEE30F4DF9C; **Location:** country: Panama; locality: Coiba; verbatimLocality: Sendero Los Monos; verbatimCoordinates: 7° 36' 2.891''N 81° 43' 35.187''O; **Event:** eventDate: 29-01-22
- **Type status:** Other material. **Occurrence:** recordedBy: Daniel Murcia & Dumas Galvez; sex: 4 immature; occurrenceID: 3D65F298-7202-559C-B16A-6E023AF6076D; **Location:** country: Panama; locality: Coiba; verbatimLocality: Sendero Los Monos; verbatimCoordinates: 7° 36' 2.891''N 81° 43' 35.187''O; **Event:** eventDate: 18-10-22
- **Type status:** Other material. **Occurrence:** recordedBy: Daniel Murcia & Dumas Galvez; sex: 1 immature; occurrenceID: FA5894F7-FC1F-5DD0-93EA-029CDFAAACD7; **Location:** country: Panama; locality: Coiba; verbatimLocality: Sendero Los Monos; verbatimCoordinates: 7° 36' 4.903''N 81° 43' 29.06''O; **Event:** eventDate: 8-12-22

#### Castianeira sp. 1

1BB1204C-AD38-5056-A63B-4395A84BAA4A

##### Materials

- **Type status:** Other material. **Occurrence:** recordedBy: Daniel Murcia & Dumas Galvez; sex: 1 female; occurrenceID: BA58A453-A449-58C7-AE62-DC86D489176E; **Location:** country: Panama; locality: Coiba; verbatimLocality: Sendero de Coiba AIP; verbatimCoordinates: 7° 36' 4.903''N 81° 43' 29.06''O; **Event:** eventDate: 24-03-22

#### Castianeira sp. 2

D82FF2AE-FC67-5741-9739-E000A36D595E

##### Materials

- **Type status:** Other material. **Occurrence:** recordedBy: Daniel Murcia & Dumas Galvez; sex: 1 immature; occurrenceID: BE6D581A-1427-55E3-A936-BA606896D365; **Location:** country: Panama; locality: Coiba; verbatimLocality: Playa Hermosa; verbatimCoordinates: 7° 30' 53.708''N 81° 52' 0.411''O; **Event:** eventDate: 1-07-22
- **Type status:** Other material. **Occurrence:** recordedBy: Daniel Murcia & Dumas Galvez; sex: 1 immature; occurrenceID: BD89565C-F3D7-53E9-B5D5-87ECFDB54336; **Location:** country: Panama; locality: Coiba; verbatimLocality: Isla Rancheria; verbatimCoordinates: 7° 38' 14.867''N 81° 42' 10.497''O; **Event:** eventDate: 24-03-22

#### Corinna bulbosa

F. O. Pickard-Cambridge, 1899

6A12454A-8C5B-52BF-BD49-AD0D6861785A

##### Materials

- **Type status:** Other material. **Occurrence:** recordedBy: Daniel Murcia & Dumas Galvez; sex: 1 male; occurrenceID: 0AC2D24D-CC7A-5F41-B1CC-39F0258511F3; **Location:** country: Panama; locality: Coiba; verbatimLocality: Sendero de Coiba AIP; verbatimCoordinates: 7° 36' 4.903''N 81° 43' 29.06''O; **Event:** eventDate: 6-12-22
- **Type status:** Other material. **Occurrence:** recordedBy: Daniel Murcia & Dumas Galvez; sex: 1 female; occurrenceID: 561DD17B-5F33-5113-BAE7-4B7F6EA87941; **Location:** country: Panama; locality: Coiba; verbatimLocality: Sendero Los Monos; verbatimCoordinates: 7° 36' 2.891''N 81° 43' 35.187''O; **Event:** eventDate: 18-10-22
- **Type status:** Other material. **Occurrence:** recordedBy: Daniel Murcia & Dumas Galvez; sex: 1 male; occurrenceID: B111A17C-EF42-5997-9F0D-FAFA555D21D2; **Location:** country: Panama; locality: Coiba; verbatimLocality: Sendero de Coiba AIP; verbatimCoordinates: 7° 36' 4.903''N 81° 43' 29.06''O; **Event:** eventDate: 7-06-23

##### Distribution

Mexico to Panama

##### Notes

MX-CA

#### Corinna sp. 1

9C02515A-5ED9-52C1-8C47-26DB30394870

##### Materials

- **Type status:** Other material. **Occurrence:** recordedBy: Daniel Murcia & Dumas Galvez; sex: 1 female; occurrenceID: 8B4609A2-0060-57B3-9471-A1F20EC6324B; **Location:** country: Panama; locality: Coiba; verbatimLocality: Playa Hermosa; verbatimCoordinates: 7° 30' 53.708''N 81° 52' 0.411''O; **Event:** eventDate: 26-03-22

#### Creugas cf. mucronatus

9D9CC911-120A-5A0D-834B-2ECEA3379895

##### Materials

- **Type status:** Other material. **Occurrence:** recordedBy: Daniel Murcia & Dumas Galvez; sex: 1 female; occurrenceID: 034BBD18-D8D1-5DCA-87D9-12CD9E4DD943; **Location:** country: Panama; locality: Coiba; verbatimLocality: Sendero Los Monos; verbatimCoordinates: 7° 36' 2.891''N 81° 43' 35.187''O; **Event:** eventDate: 8-12-22

#### Mazax spinosa

(Simon, 1898)

006A619D-1E92-5D5C-A032-59A07CBE6EA9

##### Materials

- **Type status:** Other material. **Occurrence:** recordedBy: Daniel Murcia & Dumas Galvez; sex: 1 immature; occurrenceID: 2E3981E3-2CBC-5C14-8B69-3F1EB1A52E80; **Location:** country: Panama; locality: Coiba; verbatimLocality: Est. Coiba AIP Principal; verbatimCoordinates: 7° 36' 0.461''N 81° 43' 27.094''O; **Event:** eventDate: 25-08-21
- **Type status:** Other material. **Occurrence:** recordedBy: Daniel Murcia & Dumas Galvez; sex: 1 male; occurrenceID: F069A072-1634-5170-A0CF-5C23DE33C1E8; **Location:** country: Panama; locality: Coiba; verbatimLocality: Est. MiAmbiente Principal; verbatimCoordinates: 7° 37' 37.024''N 81° 43' 46.56''O; **Event:** eventDate: 25-08-21
- **Type status:** Other material. **Occurrence:** recordedBy: Daniel Murcia & Dumas Galvez; sex: 1 immature; occurrenceID: E321D702-CBCB-5433-A24A-4E40A78D45D3; **Location:** country: Panama; locality: Coiba; verbatimLocality: Isla Rancheria; verbatimCoordinates: 7° 38' 14.867''N 81° 42' 10.497''O; **Event:** eventDate: 24-03-22
- **Type status:** Other material. **Occurrence:** recordedBy: Daniel Murcia & Dumas Galvez; sex: 1 female; occurrenceID: 6906BC78-9A4A-5D2B-9519-26976C21F0F3; **Location:** country: Panama; locality: Coiba; verbatimLocality: Mirador Alto; verbatimCoordinates: 7° 37' 33.488''N 81° 43' 41.199''O; **Event:** eventDate: 26-08-21
- **Type status:** Other material. **Occurrence:** recordedBy: Daniel Murcia & Dumas Galvez; sex: 2 female, 1 immature; occurrenceID: 5B8496BA-B045-56FC-8FE6-B635D11A1146; **Location:** country: Panama; locality: Coiba; verbatimLocality: Playa Hermosa; verbatimCoordinates: 7° 30' 53.708''N 81° 52' 0.411''O; **Event:** eventDate: 25-03-22
- **Type status:** Other material. **Occurrence:** recordedBy: Daniel Murcia & Dumas Galvez; sex: 1 female; occurrenceID: 0DE9201D-C023-5F00-9391-D5019BBF632E; **Location:** country: Panama; locality: Coiba; verbatimLocality: Playa Hermosa; verbatimCoordinates: 7° 30' 53.708''N 81° 52' 0.411''O; **Event:** eventDate: 26-03-22

##### Distribution

Guatemala, Panama, St. Lucia, St. Vincent

##### Notes

CA-C

#### Simonestus sp. 1

7ED14227-820D-5A94-9878-068D665E91FC

##### Materials

- **Type status:** Other material. **Occurrence:** recordedBy: Daniel Murcia & Dumas Galvez; sex: 1 female; occurrenceID: 318E3C66-EC82-5441-BAEC-B330B209361D; **Location:** country: Panama; locality: Coiba; verbatimLocality: Sendero Los Monos; verbatimCoordinates: 7° 36' 2.891''N 81° 43' 35.187''O; **Event:** eventDate: 8-12-22
- **Type status:** Other material. **Occurrence:** recordedBy: Daniel Murcia & Dumas Galvez; sex: 1 immature; occurrenceID: AFEE1EFA-EEF5-5733-A445-E9E411FA927F; **Location:** country: Panama; locality: Coiba; verbatimLocality: Sendero Los Monos; verbatimCoordinates: 7° 36' 2.891''N 81° 43' 35.187''O; **Event:** eventDate: 7-02-23

#### Acanthoctenus lamarrei

Arizala, Labarque & Polotow, 2021

179E1A30-8F20-50FB-A467-762ED62221EC

##### Materials

- **Type status:** Other material. **Occurrence:** recordedBy: Daniel Murcia & Dumas Galvez; sex: 1 female, 1 male, 2 immature; occurrenceID: BF42E545-F400-59F7-BF1D-37E8B69CAB95; **Location:** country: Panama; locality: Coiba; verbatimLocality: Sendero de Coiba AIP; verbatimCoordinates: 7° 36' 4.903''N 81° 43' 29.06''O; **Event:** eventDate: 6-12-22
- **Type status:** Other material. **Occurrence:** recordedBy: Daniel Murcia & Dumas Galvez; sex: 2 female, 4 male; occurrenceID: EBA28470-BC5C-5E26-A5C9-5F5F8A93A826; **Location:** country: Panama; locality: Coiba; verbatimLocality: Sendero de Coiba AIP; verbatimCoordinates: 7° 36' 4.903''N 81° 43' 29.06''O; **Event:** eventDate: 7-12-22

##### Distribution

Panama (endemic)

##### Notes

PA

#### Ancylometes bogotensis

(Keyserling, 1877)

245F65E4-9791-5D4C-9870-6586575928C1

##### Materials

- **Type status:** Other material. **Occurrence:** recordedBy: Daniel Murcia & Dumas Galvez; sex: 2 female, 1 male, 3 immature; occurrenceID: 76535110-2E9C-595A-A18D-91FA54C88DE5; **Location:** country: Panama; locality: Coiba; verbatimLocality: Playa Hermosa; verbatimCoordinates: 7° 30' 53.708''N 81° 52' 0.411''O; **Event:** eventDate: 1-07-22
- **Type status:** Other material. **Occurrence:** recordedBy: Daniel Murcia & Dumas Galvez; sex: 1 female; occurrenceID: 5BD9FC83-CEE3-5D7E-B858-53867004301D; **Location:** country: Panama; locality: Coiba; verbatimLocality: Playa Hermosa; verbatimCoordinates: 7° 30' 53.708''N 81° 52' 0.411''O; **Event:** eventDate: 25-03-22
- **Type status:** Other material. **Occurrence:** recordedBy: Daniel Murcia & Dumas Galvez; sex: 1 female; occurrenceID: 922B9C93-09CF-5940-8679-468329309501; **Location:** country: Panama; locality: Coiba; verbatimLocality: Sendero Los Monos; verbatimCoordinates: 7° 36' 2.891''N 81° 43' 35.187''O; **Event:** eventDate: 29-01-22

##### Distribution

Honduras to Bolivia

##### Notes

CA-SA

#### Ctenus nigrolineatus

Berland, 1913

36E217C4-7E14-5905-A481-F15D1D9D37EB

##### Materials

- **Type status:** Other material. **Occurrence:** recordedBy: Daniel Murcia & Dumas Galvez; sex: 1 female; occurrenceID: ADEE04E1-C6C6-5E9A-9E15-B1048B7C7859; **Location:** country: Panama; locality: Coiba; verbatimLocality: Sendero de Coiba AIP; verbatimCoordinates: 7° 36' 4.903''N 81° 43' 29.06''O; **Event:** eventDate: 6-12-22
- **Type status:** Other material. **Occurrence:** recordedBy: Daniel Murcia & Dumas Galvez; sex: 2 immature; occurrenceID: 9F1E7D1F-90BC-59D3-A9BF-4747FD48A216; **Location:** country: Panama; locality: Coiba; verbatimLocality: Sendero de Coiba AIP; verbatimCoordinates: 7° 36' 4.903''N 81° 43' 29.06''O; **Event:** eventDate: 7-12-22
- **Type status:** Other material. **Occurrence:** recordedBy: Daniel Murcia & Dumas Galvez; sex: 1 female; occurrenceID: 81CDFEB2-C234-51E2-A171-99732A573075; **Location:** country: Panama; locality: Coiba; verbatimLocality: Sendero de Coiba AIP; verbatimCoordinates: 7° 36' 4.903''N 81° 43' 29.06''O; **Event:** eventDate: 24-03-22
- **Type status:** Other material. **Occurrence:** recordedBy: Daniel Murcia & Dumas Galvez; sex: 1 male; occurrenceID: AE020F11-037C-5DC1-B5FB-870B6BCA9730; **Location:** country: Panama; locality: Coiba; verbatimLocality: Sendero Los Monos; verbatimCoordinates: 7° 36' 2.891''N 81° 43' 35.187''O; **Event:** eventDate: 26-08-21
- **Type status:** Other material. **Occurrence:** recordedBy: Daniel Murcia & Dumas Galvez; sex: 1 female; occurrenceID: 3ED93DD1-5CE5-53A3-84C6-EE0214616DB2; **Location:** country: Panama; locality: Coiba; verbatimLocality: Isla Jicaron; verbatimCoordinates: 7° 17' 16.022''N 81° 46' 25.359''O; **Event:** eventDate: 8-12-23

##### Distribution

Ecuador

##### Notes

PA, EC, First record for Panama

#### Gen. 1 sp. 1

A6278CC3-58C0-55AC-BE5E-40C003E883B9

##### Materials

- **Type status:** Other material. **Occurrence:** recordedBy: Daniel Murcia & Dumas Galvez; sex: 1 male; occurrenceID: 42B74417-1001-5947-BE2D-9608CEB8A6D4; **Location:** country: Panama; locality: Coiba; verbatimLocality: Playa Hermosa; verbatimCoordinates: 7° 30' 53.708''N 81° 52' 0.411''O; **Event:** eventDate: 25-03-22

##### Notes

PA

#### Kiekie barrocolorado

Polotow & Brescovit, 2018

D642A7A1-45BA-579B-8685-FA548F64525C

##### Materials

- **Type status:** Other material. **Occurrence:** recordedBy: Daniel Murcia & Dumas Galvez; sex: 1 immature; occurrenceID: 776864EB-9AB4-57B2-B627-7EF8E02A70A8; **Location:** country: Panama; locality: Coiba; verbatimLocality: Est. MiAmbiente Principal; verbatimCoordinates: 7° 37' 37.024''N 81° 43' 46.56''O; **Event:** eventDate: 25-08-21
- **Type status:** Other material. **Occurrence:** recordedBy: Daniel Murcia & Dumas Galvez; sex: 1 female; occurrenceID: B7480A13-30C6-5719-8794-47FDFB8778D4; **Location:** country: Panama; locality: Coiba; verbatimLocality: Playa Hermosa; verbatimCoordinates: 7° 30' 53.708''N 81° 52' 0.411''O; **Event:** eventDate: 26-03-22
- **Type status:** Other material. **Occurrence:** recordedBy: Daniel Murcia & Dumas Galvez; sex: 1 male; occurrenceID: 4418ACAF-49B8-5575-91E1-BEFF4C53D8EB; **Location:** country: Panama; locality: Coiba; verbatimLocality: Sendero de Coiba AIP; verbatimCoordinates: 7° 36' 4.903''N 81° 43' 29.06''O; **Event:** eventDate: 24-03-22
- **Type status:** Other material. **Occurrence:** recordedBy: Daniel Murcia & Dumas Galvez; sex: 2 male; occurrenceID: 5A235C02-B5D7-596F-99B2-ABE419DB1FBA; **Location:** country: Panama; locality: Coiba; verbatimLocality: Sendero Los Monos; verbatimCoordinates: 7° 36' 2.891''N 81° 43' 35.187''O; **Event:** eventDate: 19-10-22
- **Type status:** Other material. **Occurrence:** recordedBy: Daniel Murcia & Dumas Galvez; sex: 1 male; occurrenceID: 136F49C6-5D62-516F-A7A8-A8C93DEE00B8; **Location:** country: Panama; locality: Coiba; verbatimLocality: Sendero Santa Cruz; verbatimCoordinates: 7° 37' 32.47''N 81° 43' 51.632''O; **Event:** eventDate: 25-08-21
- **Type status:** Other material. **Occurrence:** recordedBy: Daniel Murcia & Dumas Galvez; sex: 1 female, 1 immature; occurrenceID: 468A8FC7-A648-54D8-9A3D-77E45CAB74BC; **Location:** country: Panama; locality: Coiba; verbatimLocality: Sendero Los Monos; verbatimCoordinates: 7° 36' 2.891''N 81° 43' 35.187''O; **Event:** eventDate: 8-12-22

##### Distribution

Panama (endemic)

##### Notes

PA

#### Kiekie panamensis

Polotow & Brescovit, 2018

85EC3AF9-3B64-5696-97D0-5ACB93ED69B0

##### Materials

- **Type status:** Other material. **Occurrence:** recordedBy: Daniel Murcia & Dumas Galvez; sex: 1 female, 1 male; occurrenceID: 7DB99689-5BC1-5941-8208-D27BDB37AB3A; **Location:** country: Panama; locality: Coiba; verbatimLocality: Playa Hermosa; verbatimCoordinates: 7° 30' 53.708''N 81° 52' 0.411''O; **Event:** eventDate: 25-03-22

##### Distribution

Panama (endemic)

##### Notes

CA-SA

#### Phoneutria depilata

(Strand, 1909)

4543D2F2-0038-5276-BE53-346E0150401F

##### Materials

- **Type status:** Other material. **Occurrence:** recordedBy: Daniel Murcia & Dumas Galvez; sex: 1 female; occurrenceID: 146BB78B-5FF8-576A-98B9-88014B0F44DD; **Location:** country: Panama; locality: Coiba; verbatimLocality: Playa Hermosa; verbatimCoordinates: 7° 30' 53.708''N 81° 52' 0.411''O; **Event:** eventDate: 26-03-22
- **Type status:** Other material. **Occurrence:** recordedBy: Daniel Murcia & Dumas Galvez; sex: 1 immature; occurrenceID: 5A8DA959-AD88-595C-BCEF-BE00B148A561; **Location:** country: Panama; locality: Coiba; verbatimLocality: Sendero Los Monos; verbatimCoordinates: 7° 36' 2.891''N 81° 43' 35.187''O; **Event:** eventDate: 29-01-22
- **Type status:** Other material. **Occurrence:** recordedBy: Daniel Murcia & Dumas Galvez; sex: 1 male; occurrenceID: F0CCC73E-3845-51C0-8367-FA27F1CE34FC; **Location:** country: Panama; locality: Coiba; verbatimLocality: Sendero Los Monos; verbatimCoordinates: 7° 36' 2.891''N 81° 43' 35.187''O; **Event:** eventDate: 8-12-22
- **Type status:** Other material. **Occurrence:** recordedBy: Daniel Murcia & Dumas Galvez; sex: 1 female; occurrenceID: 0690EAC1-6EFC-5AD1-B315-F0145FB080B1; **Location:** country: Panama; locality: Coiba; verbatimLocality: Sendero Los Monos; verbatimCoordinates: 7° 36' 2.891''N 81° 43' 35.187''O; **Event:** eventDate: 7-02-23
- **Type status:** Other material. **Occurrence:** recordedBy: Daniel Murcia & Dumas Galvez; sex: 2 male; occurrenceID: C5DAB046-6850-5260-A55C-65550C2C3A38; **Location:** country: Panama; locality: Coiba; verbatimLocality: Sendero Los Monos; verbatimCoordinates: 7° 36' 2.891''N 81° 43' 35.187''O; **Event:** eventDate: 7-02-23

##### Distribution

Guatemala, Honduras, Nicaragua, Costa Rica, Panama, Colombia, Ecuador

#### Bolostromus panamanus

(Petrunkevitch, 1925)

6DB8906F-869C-5311-90B9-0A9E5542063D

##### Materials

- **Type status:** Other material. **Occurrence:** recordedBy: Daniel Murcia & Dumas Galvez; sex: 1 female; occurrenceID: 24EAC612-8ED5-5259-8120-267DFB64DB49; **Location:** country: Panama; locality: Coiba; verbatimLocality: Sendero Los Monos; verbatimCoordinates: 7° 36' 2.891''N 81° 43' 35.187''O; **Event:** eventDate: 7-12-22
- **Type status:** Other material. **Occurrence:** recordedBy: Daniel Murcia & Dumas Galvez; sex: 1 female; occurrenceID: 4653E52B-0015-5EF0-A5F9-5D883D861E30; **Location:** country: Panama; locality: Coiba; verbatimLocality: Isla Rancheria; verbatimCoordinates: 7° 38' 14.867''N 81° 42' 10.497''O; **Event:** eventDate: 24-03-22
- **Type status:** Other material. **Occurrence:** recordedBy: Daniel Murcia & Dumas Galvez; sex: 1 male; occurrenceID: 18399CE1-B85F-5633-9F54-92C7E2329783; **Location:** country: Panama; locality: Coiba; verbatimLocality: Playa Hermosa; verbatimCoordinates: 7° 30' 53.708''N 81° 52' 0.411''O; **Event:** eventDate: 26-03-22

##### Distribution

Costa Rica, Panama

##### Notes

CR-PA

#### Labahitha marginata

(Kishida, 1936)

48732FE2-C48D-59B2-B9CF-4AC851ECF32A

##### Materials

- **Type status:** Other material. **Occurrence:** recordedBy: Daniel Murcia & Dumas Galvez; sex: 1 female; occurrenceID: ECC261E8-B301-58B7-AE41-0485E138942A; **Location:** country: Panama; locality: Coiba; verbatimLocality: Isla Rancheria; verbatimCoordinates: 7° 38' 14.867''N 81° 42' 10.497''O; **Event:** eventDate: 24-03-22

##### Distribution

Taiwan, Philippines, Papua New Guinea, Pacifi Is. Introduced to Mexico, Central America, Brazil

##### Notes

MX-SA

#### Zimiromus tropicalis

(Banks, 1909)

7D0039F9-101C-5F5C-AF27-23DCAE1A3473

##### Materials

- **Type status:** Other material. **Occurrence:** recordedBy: Daniel Murcia & Dumas Galvez; sex: 1 female; occurrenceID: 298DD300-8F7F-58D2-9A48-E99820276D3B; **Location:** country: Panama; locality: Coiba; verbatimLocality: Isla Rancheria; verbatimCoordinates: 7° 38' 14.867''N 81° 42' 10.497''O; **Event:** eventDate: 24-03-22

##### Distribution

Costa Rica, Panama

##### Notes

CR-PA

#### Neotama mexicana

(O. Pickard-Cambridge, 1893)

F8C01086-BDA2-5871-ABD7-4C7F23C5CFDD

##### Materials

- **Type status:** Other material. **Occurrence:** recordedBy: Daniel Murcia & Dumas Galvez; sex: 1 female; occurrenceID: 1A42E382-05DC-5E1B-AE38-760F300B9304; **Location:** country: Panama; locality: Coiba; verbatimLocality: Sendero Los Monos; verbatimCoordinates: 7° 36' 2.891''N 81° 43' 35.187''O; **Event:** eventDate: 19-10-22
- **Type status:** Other material. **Occurrence:** recordedBy: Daniel Murcia & Dumas Galvez; sex: 1 immature; occurrenceID: 4F809216-D5C2-5586-BA08-55EDD8D31BCA; **Location:** country: Panama; locality: Coiba; verbatimLocality: Sendero Santa Cruz; verbatimCoordinates: 7° 37' 32.47''N 81° 43' 51.632''O; **Event:** eventDate: 28-08-21

##### Distribution

USA to Peru, Guyana

##### Notes

NA-SA

#### Allocosa cf. panamena

9B934C36-F1C2-5680-85BC-66FF0B8BDBB8

##### Materials

- **Type status:** Other material. **Occurrence:** recordedBy: Daniel Murcia & Dumas Galvez; sex: 2 male; occurrenceID: 00C50569-D207-5EC8-8D76-3131C00AA32C; **Location:** country: Panama; locality: Coiba; verbatimLocality: Sendero de Coiba AIP; verbatimCoordinates: 7° 36' 4.903''N 81° 43' 29.06''O; **Event:** eventDate: 7-12-22
- **Type status:** Other material. **Occurrence:** recordedBy: Daniel Murcia & Dumas Galvez; sex: 3 female, 2 male; occurrenceID: 265D721B-B450-5B15-89C9-169593339C95; **Location:** country: Panama; locality: Coiba; verbatimLocality: Est. Coiba AIP Principal; verbatimCoordinates: 7° 36' 0.461''N 81° 43' 27.094''O; **Event:** eventDate: 25-08-21
- **Type status:** Other material. **Occurrence:** recordedBy: Daniel Murcia & Dumas Galvez; sex: 1 immature; occurrenceID: C7BC608A-CFA8-5E49-BA38-95D8F6B59349; **Location:** country: Panama; locality: Coiba; verbatimLocality: Sendero Los Monos; verbatimCoordinates: 7° 36' 2.891''N 81° 43' 35.187''O; **Event:** eventDate: 29-01-22

#### Arctosa sp. 1

343F16B5-2E91-521A-A849-1F18AF780D7E

##### Materials

- **Type status:** Other material. **Occurrence:** recordedBy: Daniel Murcia & Dumas Galvez; sex: 1 male; occurrenceID: 81539C74-84BE-5DA1-89E9-B2A578F0DB0D; **Location:** country: Panama; locality: Coiba; verbatimLocality: Sendero Los Monos; verbatimCoordinates: 7° 36' 2.891''N 81° 43' 35.187''O; **Event:** eventDate: 16-09-21

#### Hogna sp. 1

76CE10A6-3DDF-5CB7-8DDC-A829EA75CB6D

##### Materials

- **Type status:** Other material. **Occurrence:** recordedBy: Daniel Murcia & Dumas Galvez; sex: 2 male; occurrenceID: 4BBCC9D9-2357-5FDC-BB4B-910E0F97BB87; **Location:** country: Panama; locality: Coiba; verbatimLocality: Antigua Carcel Principal; verbatimCoordinates: 7° 30' 25.049''N 81° 42' 5.065''O; **Event:** eventDate: 25-08-21
- **Type status:** Other material. **Occurrence:** recordedBy: Daniel Murcia & Dumas Galvez; sex: 1 female; occurrenceID: 0D65A9AB-B468-5A87-8067-EA4B6779514E; **Location:** country: Panama; locality: Coiba; verbatimLocality: Est. MiAmbiente Principal; verbatimCoordinates: 7° 37' 37.024''N 81° 43' 46.56''O; **Event:** eventDate: 25-08-21

#### Gelanor zonatus

(C. L. Koch, 1845)

2AC5A800-49DD-55A7-9771-502C181A7588

##### Materials

- **Type status:** Other material. **Occurrence:** recordedBy: Daniel Murcia & Dumas Galvez; sex: 1 immature; occurrenceID: 940EA213-0F6C-55A4-86F5-0D950F73EB74; **Location:** country: Panama; locality: Coiba; verbatimLocality: Sendero Los Monos; verbatimCoordinates: 7° 36' 2.891''N 81° 43' 35.187''O; **Event:** eventDate: 19-10-22
- **Type status:** Other material. **Occurrence:** recordedBy: Daniel Murcia & Dumas Galvez; sex: 2 male; occurrenceID: EA191453-60D1-5068-A671-C5B1706A9600; **Location:** country: Panama; locality: Coiba; verbatimLocality: Sendero Los Monos; verbatimCoordinates: 7° 36' 2.891''N 81° 43' 35.187''O; **Event:** eventDate: 29-01-22
- **Type status:** Other material. **Occurrence:** recordedBy: Daniel Murcia & Dumas Galvez; sex: 1 male; occurrenceID: 35629E32-BBEF-5752-98DB-FBD86A6D257B; **Location:** country: Panama; locality: Coiba; verbatimLocality: Sendero Los Monos; verbatimCoordinates: 7° 36' 2.891''N 81° 43' 35.187''O; **Event:** eventDate: 8-02-23

##### Distribution

Mexico to Uruguay

##### Notes

MX-SA

#### Mimetus trituberculatus

O. Pickard-Cambridge, 1899

1EE78470-430E-56A6-9A05-1E8A7E08EAD6

##### Materials

- **Type status:** Other material. **Occurrence:** recordedBy: Daniel Murcia & Dumas Galvez; sex: 2 female, 1 male; occurrenceID: 78ED0D6A-633B-5D7B-84CA-C304E1FEC063; **Location:** country: Panama; locality: Coiba; verbatimLocality: Playa Hermosa; verbatimCoordinates: 7° 30' 53.708''N 81° 52' 0.411''O; **Event:** eventDate: 1-07-22
- **Type status:** Other material. **Occurrence:** recordedBy: Daniel Murcia & Dumas Galvez; sex: 1 female; occurrenceID: A550BF52-ECB6-583F-AC15-EB385ECAE8D2; **Location:** country: Panama; locality: Coiba; verbatimLocality: Sendero de Coiba AIP; verbatimCoordinates: 7° 36' 4.903''N 81° 43' 29.06''O; **Event:** eventDate: 6-12-22
- **Type status:** Other material. **Occurrence:** recordedBy: Daniel Murcia & Dumas Galvez; sex: 1 male; occurrenceID: 3C4AADE1-4E32-5218-9732-95B0BDCBB808; **Location:** country: Panama; locality: Coiba; verbatimLocality: Est. MiAmbiente Principal; verbatimCoordinates: 7° 37' 37.024''N 81° 43' 46.56''O; **Event:** eventDate: 29-01-22
- **Type status:** Other material. **Occurrence:** recordedBy: Daniel Murcia & Dumas Galvez; sex: 1 female; occurrenceID: 08E62BBA-9930-5C38-AB5A-2740C1ECDEAA; **Location:** country: Panama; locality: Coiba; verbatimLocality: Isla Rancheria; verbatimCoordinates: 7° 38' 14.867''N 81° 42' 10.497''O; **Event:** eventDate: 24-03-22
- **Type status:** Other material. **Occurrence:** recordedBy: Daniel Murcia & Dumas Galvez; sex: 1 female; occurrenceID: AFA3DA5D-8B5B-56FC-8A52-67AE02EF21E6; **Location:** country: Panama; locality: Coiba; verbatimLocality: Sendero Los Monos; verbatimCoordinates: 7° 36' 2.891''N 81° 43' 35.187''O; **Event:** eventDate: 18-10-22
- **Type status:** Other material. **Occurrence:** recordedBy: Daniel Murcia & Dumas Galvez; sex: 1 male, 1 immature; occurrenceID: C1B5A6CF-62CC-51FC-B0AA-3BD765E7C9D8; **Location:** country: Panama; locality: Coiba; verbatimLocality: Sendero Los Monos; verbatimCoordinates: 7° 36' 2.891''N 81° 43' 35.187''O; **Event:** eventDate: 19-10-22
- **Type status:** Other material. **Occurrence:** recordedBy: Daniel Murcia & Dumas Galvez; sex: 2 female; occurrenceID: F46388B6-84F0-50FF-A96C-9BB0DB408941; **Location:** country: Panama; locality: Coiba; verbatimLocality: Sendero Los Monos; verbatimCoordinates: 7° 36' 2.891''N 81° 43' 35.187''O; **Event:** eventDate: 7-02-23
- **Type status:** Other material. **Occurrence:** recordedBy: Daniel Murcia & Dumas Galvez; sex: 1 immature; occurrenceID: 20F9AC54-D1FF-59E8-8DD3-618DE42A6FAA; **Location:** country: Panama; locality: Coiba; verbatimLocality: Sendero Los Monos; verbatimCoordinates: 7° 36' 2.891''N 81° 43' 35.187''O; **Event:** eventDate: 8-02-23

##### Distribution

Panama (endemic)

##### Notes

PA

#### Mimetus verecundus

Chickering, 1947

025AED93-ED00-59D3-A0EC-3EF1AAC224FD

##### Materials

- **Type status:** Other material. **Occurrence:** recordedBy: Daniel Murcia & Dumas Galvez; sex: 1 female; occurrenceID: 5D79FA29-74EA-523D-A6D0-C7559677E013; **Location:** country: Panama; locality: Coiba; verbatimLocality: Sendero Los Monos; verbatimCoordinates: 7° 36' 2.891''N 81° 43' 35.187''O; **Event:** eventDate: 7-02-23

##### Distribution

Panama (endemic)

##### Notes

PA

#### Trichonephila clavipes

(Linnaeus, 1767)

F8363E5B-51B1-5E47-9633-63FACFC15E1F

##### Materials

- **Type status:** Other material. **Occurrence:** recordedBy: Daniel Murcia & Dumas Galvez; sex: 1 female, 1 male; occurrenceID: 0D3B409D-D86C-5F18-B986-702BE87CD754; **Location:** country: Panama; locality: Coiba; verbatimLocality: Isla Canales Afuera; verbatimCoordinates: 7° 41' 15.77''N 81° 37' 47.539''O; **Event:** eventDate: 27-08-21

##### Distribution

USA to Argentina. Introduced to São Tomé and Príncipe

##### Notes

NA-SA

#### Costarina cf. recondita

D23A1C27-BFE3-5089-8B56-5816CA0E10D2

##### Materials

- **Type status:** Other material. **Occurrence:** recordedBy: Daniel Murcia & Dumas Galvez; sex: 1 female; occurrenceID: E7EC5845-BC7A-559D-9691-E07F454E5646; **Location:** country: Panama; locality: Coiba; verbatimLocality: Sendero de Coiba AIP; verbatimCoordinates: 7° 36' 4.903''N 81° 43' 29.06''O; **Event:** eventDate: 7-12-22

#### Ponsoonops sp. 1

B8CC01F2-D48C-5792-8C97-D7316897FA6D

##### Materials

- **Type status:** Other material. **Occurrence:** recordedBy: Daniel Murcia & Dumas Galvez; sex: 1 male; occurrenceID: 1FEE7713-E1C6-5821-96C8-56BC69032965; **Location:** country: Panama; locality: Coiba; verbatimLocality: Sendero de Coiba AIP; verbatimCoordinates: 7° 36' 4.903''N 81° 43' 29.06''O; **Event:** eventDate: 7-12-22

#### Hamataliwa sp. 1

D90F3EE8-2686-5533-B953-3B2DC5550EB1

##### Materials

- **Type status:** Other material. **Occurrence:** recordedBy: Daniel Murcia & Dumas Galvez; sex: 1 immature; occurrenceID: 51EAECCE-BF7F-59E1-86A3-F9B9C4DB6405; **Location:** country: Panama; locality: Coiba; verbatimLocality: Sendero Los Monos; verbatimCoordinates: 7° 36' 2.891''N 81° 43' 35.187''O; **Event:** eventDate: 29-01-22

#### Metagonia delicata

(O. Pickard-Cambridge, 1895)

AF4C9D42-8DEB-5D87-AA57-8DEF4842D123

##### Materials

- **Type status:** Other material. **Occurrence:** recordedBy: Daniel Murcia & Dumas Galvez; sex: 1 female; occurrenceID: 76762A0E-F57C-5D80-A259-17697259E4D4; **Location:** country: Panama; locality: Coiba; verbatimLocality: Playa Hermosa; verbatimCoordinates: 7° 30' 53.708''N 81° 52' 0.411''O; **Event:** eventDate: 1-07-22
- **Type status:** Other material. **Occurrence:** recordedBy: Daniel Murcia & Dumas Galvez; sex: 1 male; occurrenceID: 50E2A2A5-29E9-54AC-93E5-902243F6A756; **Location:** country: Panama; locality: Coiba; verbatimLocality: Sendero de Coiba AIP; verbatimCoordinates: 7° 36' 4.903''N 81° 43' 29.06''O; **Event:** eventDate: 6-12-22
- **Type status:** Other material. **Occurrence:** recordedBy: Daniel Murcia & Dumas Galvez; sex: 1 immature; occurrenceID: 62AADBCB-8825-500E-8F74-6A6C95101149; **Location:** country: Panama; locality: Coiba; verbatimLocality: Playa Hermosa; verbatimCoordinates: 7° 30' 53.708''N 81° 52' 0.411''O; **Event:** eventDate: 26-03-22
- **Type status:** Other material. **Occurrence:** recordedBy: Daniel Murcia & Dumas Galvez; sex: 1 immature; occurrenceID: 0A700E0E-416F-53F3-8F1D-6FDECF10377B; **Location:** country: Panama; locality: Coiba; verbatimLocality: Playa Hermosa; verbatimCoordinates: 7° 30' 53.708''N 81° 52' 0.411''O; **Event:** eventDate: 27-03-22
- **Type status:** Other material. **Occurrence:** recordedBy: Daniel Murcia & Dumas Galvez; sex: 1 female; occurrenceID: 46947813-84E7-57C5-8FB9-4FC79099ABC8; **Location:** country: Panama; locality: Coiba; verbatimLocality: Sendero Los Monos; verbatimCoordinates: 7° 36' 2.891''N 81° 43' 35.187''O; **Event:** eventDate: 18-10-22

##### Distribution

Mexico to Panama

#### Metagonia sp. 1

07E898BA-B1DD-5F26-AE2A-50E67D31C724

##### Materials

- **Type status:** Other material. **Occurrence:** recordedBy: Daniel Murcia & Dumas Galvez; sex: 1 immature; occurrenceID: F7B6ACDF-BFF6-51E8-B224-56CF8D5A7CD6; **Location:** country: Panama; locality: Coiba; verbatimLocality: Playa Hermosa; verbatimCoordinates: 7° 30' 53.708''N 81° 52' 0.411''O; **Event:** eventDate: 1-07-22

#### Modisimus cf. guatuso

89A88E53-71BF-51EB-B6C1-D068E3B3E258

##### Materials

- **Type status:** Other material. **Occurrence:** recordedBy: Daniel Murcia & Dumas Galvez; sex: 1 male; occurrenceID: 3F7F8CA6-8688-5A25-A630-59E4E7FD40FA; **Location:** country: Panama; locality: Coiba; verbatimLocality: Sendero Los Monos; verbatimCoordinates: 7° 36' 2.891''N 81° 43' 35.187''O; **Event:** eventDate: 18-10-22
- **Type status:** Other material. **Occurrence:** recordedBy: Daniel Murcia & Dumas Galvez; sex: 1 male; occurrenceID: D49D1C0B-43B1-5A9F-932E-56CFFD11892E; **Location:** country: Panama; locality: Coiba; verbatimLocality: Sendero Los Monos; verbatimCoordinates: 7° 36' 2.891''N 81° 43' 35.187''O; **Event:** eventDate: 19-10-22

##### Notes

MX-CA

#### Modisimus sp. 1

2794943B-8791-55C4-8557-33ABA1826EEA

##### Materials

- **Type status:** Other material. **Occurrence:** recordedBy: Daniel Murcia & Dumas Galvez; sex: 2 female; occurrenceID: FF44C61C-640E-59EE-9F8C-2FE35119D6F0; **Location:** country: Panama; locality: Coiba; verbatimLocality: Playa Hermosa; verbatimCoordinates: 7° 30' 53.708''N 81° 52' 0.411''O; **Event:** eventDate: 26-03-22

#### Physocyclus sp. 1

1EE5C53C-5E1E-5542-BC06-4C6997F2AA9E

##### Materials

- **Type status:** Other material. **Occurrence:** recordedBy: Daniel Murcia & Dumas Galvez; sex: 2 female; occurrenceID: 0972761A-DDFD-5065-A096-5D2901CADEB5; **Location:** country: Panama; locality: Coiba; verbatimLocality: Est. MiAmbiente Principal; verbatimCoordinates: 7° 37' 37.024''N 81° 43' 46.56''O; **Event:** eventDate: 29-01-22
- **Type status:** Other material. **Occurrence:** recordedBy: Daniel Murcia & Dumas Galvez; sex: 1 female; occurrenceID: FB402EC7-6C0E-5693-8D6E-DE8D7D07D914; **Location:** country: Panama; locality: Coiba; verbatimLocality: Isla Rancheria; verbatimCoordinates: 7° 38' 14.867''N 81° 42' 10.497''O; **Event:** eventDate: 24-03-22

#### Acragas peckhami

(Chickering, 1946)

7E76765A-AC5C-55EE-A250-55CC57CF411B

##### Materials

- **Type status:** Other material. **Occurrence:** recordedBy: Daniel Murcia & Dumas Galvez; sex: 1 male; occurrenceID: 7C67E915-BD07-5A36-980B-907DE3C99170; **Location:** country: Panama; locality: Coiba; verbatimLocality: Isla Canales Afuera; verbatimCoordinates: 7° 41' 15.77''N 81° 37' 47.539''O; **Event:** eventDate: 27-08-21
- **Type status:** Other material. **Occurrence:** recordedBy: Daniel Murcia & Dumas Galvez; sex: 1 male; occurrenceID: AA4932F2-5103-55B7-B966-36019FE12F52; **Location:** country: Panama; locality: Coiba; verbatimLocality: Isla Rancheria; verbatimCoordinates: 7° 38' 14.867''N 81° 42' 10.497''O; **Event:** eventDate: 24-03-22
- **Type status:** Other material. **Occurrence:** recordedBy: Daniel Murcia & Dumas Galvez; sex: 1 male; occurrenceID: 6CDE8C66-23FD-5298-BD0A-2017C5C34A61; **Location:** country: Panama; locality: Coiba; verbatimLocality: Playa Hermosa; verbatimCoordinates: 7° 30' 53.708''N 81° 52' 0.411''O; **Event:** eventDate: 25-03-22
- **Type status:** Other material. **Occurrence:** recordedBy: Daniel Murcia & Dumas Galvez; sex: 1 female; occurrenceID: 52B30ED7-B6F1-53ED-9B99-3F07F14D5B0E; **Location:** country: Panama; locality: Coiba; verbatimLocality: San Juan; verbatimCoordinates: 7° 27' 34.902''N 81° 43' 18.613''O; **Event:** eventDate: 26-08-21
- **Type status:** Other material. **Occurrence:** recordedBy: Daniel Murcia & Dumas Galvez; sex: 1 female; occurrenceID: E82C6865-B2C6-5D0F-A578-2FFF08948DF8; **Location:** country: Panama; locality: Coiba; verbatimLocality: Sendero Los Monos; verbatimCoordinates: 7° 36' 2.891''N 81° 43' 35.187''O; **Event:** eventDate: 29-01-22

##### Distribution

Panama, Colombia

##### Notes

PA-CO

#### Anasaitis canalis

(Chamberlin, 1925)

93EF0DC7-40CD-5175-9D13-38701465A5D6

##### Materials

- **Type status:** Other material. **Occurrence:** recordedBy: Daniel Murcia & Dumas Galvez; sex: 1 female, 1 male; occurrenceID: C96B75B7-99A2-5F23-A981-280E2EA54F50; **Location:** country: Panama; locality: Coiba; verbatimLocality: Isla Canales Afuera; verbatimCoordinates: 7° 41' 15.77''N 81° 37' 47.539''O; **Event:** eventDate: 27-08-21
- **Type status:** Other material. **Occurrence:** recordedBy: Daniel Murcia & Dumas Galvez; sex: 1 male; occurrenceID: 56CF4831-C17A-51AC-A53E-1DAE05088791; **Location:** country: Panama; locality: Coiba; verbatimLocality: Mirador Alto; verbatimCoordinates: 7° 37' 33.488''N 81° 43' 41.199''O; **Event:** eventDate: 16-09-21
- **Type status:** Other material. **Occurrence:** recordedBy: Daniel Murcia & Dumas Galvez; sex: 2 female; occurrenceID: 6881F442-47B1-5931-A694-C26EF4260226; **Location:** country: Panama; locality: Coiba; verbatimLocality: Playa Hermosa; verbatimCoordinates: 7° 30' 53.708''N 81° 52' 0.411''O; **Event:** eventDate: 25-03-22
- **Type status:** Other material. **Occurrence:** recordedBy: Daniel Murcia & Dumas Galvez; sex: 1 female; occurrenceID: 4975D5DD-2012-5F1D-81B1-AB3AE4E6F045; **Location:** country: Panama; locality: Coiba; verbatimLocality: Playa Hermosa; verbatimCoordinates: 7° 30' 53.708''N 81° 52' 0.411''O; **Event:** eventDate: 26-03-22
- **Type status:** Other material. **Occurrence:** recordedBy: Daniel Murcia & Dumas Galvez; sex: 1 female; occurrenceID: 3F996572-AA6D-5E95-AC80-E164E6BA971A; **Location:** country: Panama; locality: Coiba; verbatimLocality: Playa Hermosa; verbatimCoordinates: 7° 30' 53.708''N 81° 52' 0.411''O; **Event:** eventDate: 27-03-22

##### Distribution

Panama, Colombia

##### Notes

PA-CO

#### Chapoda gitae

Zhang & Maddison, 2012

899C7045-7D8F-5EF4-A557-A50DD66F23FF

##### Materials

- **Type status:** Other material. **Occurrence:** recordedBy: Daniel Murcia & Dumas Galvez; sex: 1 male; occurrenceID: B786D4FE-7BAF-5889-8AB0-F7C8166437F0; **Location:** country: Panama; locality: Coiba; verbatimLocality: San Juan; verbatimCoordinates: 7° 27' 34.902''N 81° 43' 18.613''O; **Event:** eventDate: 26-08-21

##### Distribution

Colombia, Ecuador

##### Notes

CO-EC,First record for Panama

#### Chapoda recondita

(G. W. Peckham & E. G. Peckham, 1896)

64442F09-537E-53C6-A18F-196642788AB3

##### Materials

- **Type status:** Other material. **Occurrence:** recordedBy: Daniel Murcia & Dumas Galvez; sex: 1 female, 1 male; occurrenceID: FB07B3AA-59A9-5944-9CA7-D391E2FBE403; **Location:** country: Panama; locality: Coiba; verbatimLocality: Playa Hermosa; verbatimCoordinates: 7° 30' 53.708''N 81° 52' 0.411''O; **Event:** eventDate: 25-03-22
- **Type status:** Other material. **Occurrence:** recordedBy: Daniel Murcia & Dumas Galvez; sex: 1 female; occurrenceID: D0429F39-85E8-5C9A-9C32-7323A13E14EA; **Location:** country: Panama; locality: Coiba; verbatimLocality: Sendero Los Monos; verbatimCoordinates: 7° 36' 2.891''N 81° 43' 35.187''O; **Event:** eventDate: 26-08-21
- **Type status:** Other material. **Occurrence:** recordedBy: Daniel Murcia & Dumas Galvez; sex: 1 female; occurrenceID: 25A279B9-8843-5FC3-9E1D-1751D3C6E92D; **Location:** country: Panama; locality: Coiba; verbatimLocality: Sendero Los Monos; verbatimCoordinates: 7° 36' 2.891''N 81° 43' 35.187''O; **Event:** eventDate: 29-01-22
- **Type status:** Other material. **Occurrence:** recordedBy: Daniel Murcia & Dumas Galvez; sex: 1 male; occurrenceID: 6AF99D77-86FC-562B-AA11-42802C9FA02B; **Location:** country: Panama; locality: Coiba; verbatimLocality: Sendero Los Monos; verbatimCoordinates: 7° 36' 2.891''N 81° 43' 35.187''O; **Event:** eventDate: 7-02-23
- **Type status:** Other material. **Occurrence:** recordedBy: Daniel Murcia & Dumas Galvez; sex: 2 female, 1 immature; occurrenceID: 11E54B69-C281-5A90-B8CE-F68816CC50C5; **Location:** country: Panama; locality: Coiba; verbatimLocality: Sendero Los Monos; verbatimCoordinates: 7° 36' 2.891''N 81° 43' 35.187''O; **Event:** eventDate: 8-02-23

##### Distribution

Guatemala, Costa Rica, Panama

##### Notes

GT, CR-PA

#### Cobanus extensus

(G. W. Peckham & E. G. Peckham, 1896)

7D0E2411-EC94-5AC0-ABA6-C2CF53C45C61

##### Materials

- **Type status:** Other material. **Occurrence:** recordedBy: Daniel Murcia & Dumas Galvez; sex: 2 female; occurrenceID: FE5269A3-5908-597D-B461-DD1319106F91; **Location:** country: Panama; locality: Coiba; verbatimLocality: Isla Canales Afuera; verbatimCoordinates: 7° 41' 15.77''N 81° 37' 47.539''O; **Event:** eventDate: 27-08-21
- **Type status:** Other material. **Occurrence:** recordedBy: Daniel Murcia & Dumas Galvez; sex: 1 female; occurrenceID: 1AA65485-0AD4-5F5E-89FE-519A847B4A57; **Location:** country: Panama; locality: Coiba; verbatimLocality: Isla Rancheria; verbatimCoordinates: 7° 38' 14.867''N 81° 42' 10.497''O; **Event:** eventDate: 24-03-22
- **Type status:** Other material. **Occurrence:** recordedBy: Daniel Murcia & Dumas Galvez; sex: 2 female; occurrenceID: 8761D4CE-16F3-5958-BE71-E20B5848512F; **Location:** country: Panama; locality: Coiba; verbatimLocality: Playa Hermosa; verbatimCoordinates: 7° 30' 53.708''N 81° 52' 0.411''O; **Event:** eventDate: 26-03-22

##### Distribution

Panama (endemic)

##### Notes

PA

#### Colonus sp. 1

48E5CCA9-3278-5998-91AB-932599E71B74

##### Materials

- **Type status:** Other material. **Occurrence:** recordedBy: Daniel Murcia & Dumas Galvez; sex: 1 immature; occurrenceID: DAFA7711-22AD-527A-B098-BC571660DC71; **Location:** country: Panama; locality: Coiba; verbatimLocality: Sendero de Coiba AIP; verbatimCoordinates: 7° 36' 4.903''N 81° 43' 29.06''O; **Event:** eventDate: 6-12-22
- **Type status:** Other material. **Occurrence:** recordedBy: Daniel Murcia & Dumas Galvez; sex: 1 immature; occurrenceID: B314D03E-DBF6-59F5-BB32-E16CA02E9AE2; **Location:** country: Panama; locality: Coiba; verbatimLocality: Playa Hermosa; verbatimCoordinates: 7° 30' 53.708''N 81° 52' 0.411''O; **Event:** eventDate: 25-03-22

#### Corythalia opima

(G. W. Peckham & E. G. Peckham, 1885)

1D9F2242-5475-5B56-9B14-1A2DC722EE4D

##### Materials

- **Type status:** Other material. **Occurrence:** recordedBy: Daniel Murcia & Dumas Galvez; sex: 1 male; occurrenceID: 5CBB7DA1-E3A7-536F-8A99-4A4F4539BD64; **Location:** country: Panama; locality: Coiba; verbatimLocality: Isla Rancheria; verbatimCoordinates: 7° 38' 14.867''N 81° 42' 10.497''O; **Event:** eventDate: 24-03-22
- **Type status:** Other material. **Occurrence:** recordedBy: Daniel Murcia & Dumas Galvez; sex: 1 immature; occurrenceID: 58D443F7-5ECB-58EF-8D07-9B094113A5FA; **Location:** country: Panama; locality: Coiba; verbatimLocality: Sendero Los Monos; verbatimCoordinates: 7° 36' 2.891''N 81° 43' 35.187''O; **Event:** eventDate: 29-01-22
- **Type status:** Other material. **Occurrence:** recordedBy: Daniel Murcia & Dumas Galvez; sex: 1 female; occurrenceID: C4C2B4B1-FFA0-5C8B-9FA3-0316A188A17F; **Location:** country: Panama; locality: Coiba; verbatimLocality: Sendero de Coiba AIP; verbatimCoordinates: 7° 36' 4.903''N 81° 43' 29.06''O; **Event:** eventDate: 7-06-23

##### Distribution

USA, Mexico, Guatemala, El Salvador

##### Notes

NA-CA

#### Corythalia spiralis

(F. O. Pickard-Cambridge, 1901)

51F5E80B-7768-545A-A464-281D00904B0E

##### Materials

- **Type status:** Other material. **Occurrence:** recordedBy: Daniel Murcia & Dumas Galvez; sex: 1 male; occurrenceID: 97C834BA-9724-5671-A5E4-16986133D246; **Location:** country: Panama; locality: Coiba; verbatimLocality: Isla Rancheria; verbatimCoordinates: 7° 38' 14.867''N 81° 42' 10.497''O; **Event:** eventDate: 24-03-22
- **Type status:** Other material. **Occurrence:** recordedBy: Daniel Murcia & Dumas Galvez; sex: 1 male; occurrenceID: DC15A179-A12C-5330-8749-77DEB22314B6; **Location:** country: Panama; locality: Coiba; verbatimLocality: Playa Hermosa; verbatimCoordinates: 7° 30' 53.708''N 81° 52' 0.411''O; **Event:** eventDate: 25-03-22

##### Distribution

Colombia, Venezuela, French Guiana, Brazil

##### Notes

SA

#### Corythalia sulphurea

(F. O. Pickard-Cambridge, 1901)

8034BD16-B2F3-5020-BEDF-8EDDAF16FD80

##### Materials

- **Type status:** Other material. **Occurrence:** recordedBy: Daniel Murcia & Dumas Galvez; sex: 1 female; occurrenceID: B2F17678-6746-516B-B2DA-1432090F7236; **Location:** country: Panama; locality: Coiba; verbatimLocality: Isla Canales Afuera; verbatimCoordinates: 7° 41' 15.77''N 81° 37' 47.539''O; **Event:** eventDate: 30-01-22

##### Distribution

Costa Rica, Panama

##### Notes

CR-PA

#### Habronattus mexicanus

(G. W. Peckham & E. G. Peckham, 1896)

F00DEC9E-933F-5A3A-BB22-FD43A053AC5F

##### Materials

- **Type status:** Other material. **Occurrence:** recordedBy: Daniel Murcia & Dumas Galvez; sex: 1 female; occurrenceID: E8FEC9CB-1BCD-5A08-8ABD-35BF0C1038DF; **Location:** country: Panama; locality: Coiba; verbatimLocality: Antigua Carcel Principal; verbatimCoordinates: 7° 30' 25.049''N 81° 42' 5.065''O; **Event:** eventDate: 25-08-21

##### Distribution

USA to Panama, Caribbean

##### Notes

NA-C-CA

#### Leptofreya bifurcata

(F. O. Pickard-Cambridge, 1901)

02FC162B-1AF8-5B96-9E1A-2A8F80A9E290

##### Materials

- **Type status:** Other material. **Occurrence:** recordedBy: Daniel Murcia & Dumas Galvez; sex: 1 male; occurrenceID: F220BCD6-FABB-5C64-9415-53B5DC2BA376; **Location:** country: Panama; locality: Coiba; verbatimLocality: Isla Rancheria; verbatimCoordinates: 7° 38' 14.867''N 81° 42' 10.497''O; **Event:** eventDate: 24-03-22

##### Distribution

Mexico, Panama

##### Notes

MX, PA

#### Lyssomanes sp. 1

24D747BD-7719-5EDD-803F-64B98F6F2768

##### Materials

- **Type status:** Other material. **Occurrence:** recordedBy: Daniel Murcia & Dumas Galvez; sex: 1 immature; occurrenceID: D1B4E4F0-CD71-53A5-8746-0F641D1B3D2A; **Location:** country: Panama; locality: Coiba; verbatimLocality: Est. MiAmbiente Principal; verbatimCoordinates: 7° 37' 37.024''N 81° 43' 46.56''O; **Event:** eventDate: 16-09-21
- **Type status:** Other material. **Occurrence:** recordedBy: Daniel Murcia & Dumas Galvez; sex: 1 immature; occurrenceID: 3471EACC-16A8-5898-8B85-096854E2FB79; **Location:** country: Panama; locality: Coiba; verbatimLocality: Mirador Alto; verbatimCoordinates: 7° 37' 33.488''N 81° 43' 41.199''O; **Event:** eventDate: 16-09-21
- **Type status:** Other material. **Occurrence:** recordedBy: Daniel Murcia & Dumas Galvez; sex: 1 immature; occurrenceID: 81BC818A-4184-5EDD-825F-B13C1092E025; **Location:** country: Panama; locality: Coiba; verbatimLocality: Playa Hermosa; verbatimCoordinates: 7° 30' 53.708''N 81° 52' 0.411''O; **Event:** eventDate: 25-03-22
- **Type status:** Other material. **Occurrence:** recordedBy: Daniel Murcia & Dumas Galvez; sex: 1 immature; occurrenceID: CE64CE45-79FC-5A38-B27D-E3C30C45E8A0; **Location:** country: Panama; locality: Coiba; verbatimLocality: Sendero Los Monos; verbatimCoordinates: 7° 36' 2.891''N 81° 43' 35.187''O; **Event:** eventDate: 26-08-21
- **Type status:** Other material. **Occurrence:** recordedBy: Daniel Murcia & Dumas Galvez; sex: 1 immature; occurrenceID: 6C5C7CCF-A9A6-5725-94E8-C0D91919AD9A; **Location:** country: Panama; locality: Coiba; verbatimLocality: Sendero Los Monos; verbatimCoordinates: 7° 36' 2.891''N 81° 43' 35.187''O; **Event:** eventDate: 29-01-22

#### Menemerus bivittatus

(Dufour, 1831)

C9D48723-8147-57FE-A1F4-30AC3E426909

##### Materials

- **Type status:** Other material. **Occurrence:** recordedBy: Daniel Murcia & Dumas Galvez; sex: 1 female; occurrenceID: 3AD65345-92BA-5FB7-B882-CA4D00C83C62; **Location:** country: Panama; locality: Coiba; verbatimLocality: Isla Rancheria; verbatimCoordinates: 7° 38' 14.867''N 81° 42' 10.497''O; **Event:** eventDate: 24-03-22

##### Distribution

Africa. Introduced to North, Central and South America, southern Europe, Turkey, India, China, Taiwan, Japan, Australia, Pacific Is.

##### Notes

NA-CA-SA

#### Myrmapana panamensis

(Galiano, 1969)

0A3C0CCC-FBB6-5BD6-A956-B58425C8414B

##### Materials

- **Type status:** Other material. **Occurrence:** recordedBy: Daniel Murcia & Dumas Galvez; sex: 1 male, 1 immature; occurrenceID: 1D44DE73-5332-57B7-8D07-142BCA0AD42F; **Location:** country: Panama; locality: Coiba; verbatimLocality: Est. MiAmbiente Principal; verbatimCoordinates: 7° 37' 37.024''N 81° 43' 46.56''O; **Event:** eventDate: 16-09-21

##### Distribution

Panama, Argentina

##### Notes

PA, AR

#### Noegus spiralifer

(F. O. Pickard-Cambridge, 1901)

B1CF4099-8E33-5097-A679-1278268AAF79

##### Materials

- **Type status:** Other material. **Occurrence:** recordedBy: Daniel Murcia & Dumas Galvez; sex: 1 male; occurrenceID: 764003A6-0398-53F9-BAFD-CBF4A8BC4508; **Location:** country: Panama; locality: Coiba; verbatimLocality: Sendero Los Monos; verbatimCoordinates: 7° 36' 2.891''N 81° 43' 35.187''O; **Event:** eventDate: 29-01-22

##### Distribution

Guatemala, Panama

##### Notes

GU, PA

#### Psecas sp. 1

4317AD0F-4D53-5B5E-A741-423635FDF8CD

##### Materials

- **Type status:** Other material. **Occurrence:** recordedBy: Daniel Murcia & Dumas Galvez; sex: 1 immature; occurrenceID: 9118715F-CBC5-5049-9FCB-780FC6D09ED1; **Location:** country: Panama; locality: Coiba; verbatimLocality: Sendero de Coiba AIP; verbatimCoordinates: 7° 36' 4.903''N 81° 43' 29.06''O; **Event:** eventDate: 6-12-22

#### Sarinda nigra

G. W. Peckham & E. G. Peckham, 1892

D076CCED-DDB6-5CAE-9219-956A2DF359F3

##### Materials

- **Type status:** Other material. **Occurrence:** recordedBy: Daniel Murcia & Dumas Galvez; sex: 2 female, 1 male, 1 immature; occurrenceID: 2D8E1941-2FFF-5327-A7BC-14E81EC793F7; **Location:** country: Panama; locality: Coiba; verbatimLocality: Playa Hermosa; verbatimCoordinates: 7° 30' 53.708''N 81° 52' 0.411''O; **Event:** eventDate: 25-03-22

##### Distribution

Nicaragua, Guyana, Brazil, Paraguay, Argentina

##### Notes

NI, SA, First record for Panama

#### Sidusa flavens

(G. W. Peckham & E. G. Peckham, 1896)

DA99700A-F280-548E-A006-C2B66B7D2618

##### Materials

- **Type status:** Other material. **Occurrence:** recordedBy: Daniel Murcia & Dumas Galvez; sex: 1 immature; occurrenceID: 1104A932-BAB8-566E-9E98-0CABFE0FC926; **Location:** country: Panama; locality: Coiba; verbatimLocality: Sendero Los Monos; verbatimCoordinates: 7° 36' 2.891''N 81° 43' 35.187''O; **Event:** eventDate: 8-12-22
- **Type status:** Other material. **Occurrence:** recordedBy: Daniel Murcia & Dumas Galvez; sex: 2 male; occurrenceID: 44CC24B0-8AC5-5A84-A0AB-D977D4BCB5EE; **Location:** country: Panama; locality: Coiba; verbatimLocality: Isla Canales Afuera; verbatimCoordinates: 7° 41' 15.77''N 81° 37' 47.539''O; **Event:** eventDate: 30-01-22
- **Type status:** Other material. **Occurrence:** recordedBy: Daniel Murcia & Dumas Galvez; sex: 1 male; occurrenceID: 0578936E-587E-5B7C-96AB-446E7E42CA7B; **Location:** country: Panama; locality: Coiba; verbatimLocality: Mirador Alto; verbatimCoordinates: 7° 37' 33.488''N 81° 43' 41.199''O; **Event:** eventDate: 16-09-21
- **Type status:** Other material. **Occurrence:** recordedBy: Daniel Murcia & Dumas Galvez; sex: 2 male; occurrenceID: 5C59B780-FACD-525B-B365-1165D06309D5; **Location:** country: Panama; locality: Coiba; verbatimLocality: Mirador Alto; verbatimCoordinates: 7° 37' 33.488''N 81° 43' 41.199''O; **Event:** eventDate: 26-08-21
- **Type status:** Other material. **Occurrence:** recordedBy: Daniel Murcia & Dumas Galvez; sex: 1 male; occurrenceID: F1CD9E23-9BC4-5789-8FB1-093999AD1D4B; **Location:** country: Panama; locality: Coiba; verbatimLocality: Mirador Gambute; verbatimCoordinates: 7° 37' 41.657''N 81° 43' 59.174''O; **Event:** eventDate: 27-08-21
- **Type status:** Other material. **Occurrence:** recordedBy: Daniel Murcia & Dumas Galvez; sex: 1 male; occurrenceID: 6CBC98D6-E981-572E-B30B-C7836E68DFF2; **Location:** country: Panama; locality: Coiba; verbatimLocality: San Juan; verbatimCoordinates: 7° 27' 34.902''N 81° 43' 18.613''O; **Event:** eventDate: 16-09-21
- **Type status:** Other material. **Occurrence:** recordedBy: Daniel Murcia & Dumas Galvez; sex: 1 male; occurrenceID: 445FCD8B-34FF-5802-ACA8-C2E4DC6C95AA; **Location:** country: Panama; locality: Coiba; verbatimLocality: Sendero Los Monos; verbatimCoordinates: 7° 36' 2.891''N 81° 43' 35.187''O; **Event:** eventDate: 19-10-22
- **Type status:** Other material. **Occurrence:** recordedBy: Daniel Murcia & Dumas Galvez; sex: 1 male; occurrenceID: 96FA7ACA-A653-506D-A1A0-0F855517571E; **Location:** country: Panama; locality: Coiba; verbatimLocality: Sendero Los Monos; verbatimCoordinates: 7° 36' 2.891''N 81° 43' 35.187''O; **Event:** eventDate: 26-08-21
- **Type status:** Other material. **Occurrence:** recordedBy: Daniel Murcia & Dumas Galvez; sex: 1 male; occurrenceID: F7EBB24D-9FCD-55A5-8300-19872459F9E0; **Location:** country: Panama; locality: Coiba; verbatimLocality: Sendero Los Monos; verbatimCoordinates: 7° 36' 2.891''N 81° 43' 35.187''O; **Event:** eventDate: 29-01-22

##### Distribution

Panama (endemic)

##### Notes

PA

#### Xanthofreya albosignata

(F. O. Pickard-Cambridge, 1901)

4F30C802-398B-5D1F-9435-E409A520DF22

##### Materials

- **Type status:** Other material. **Occurrence:** recordedBy: Daniel Murcia & Dumas Galvez; sex: 1 male; occurrenceID: 6E345B8D-3E08-5739-B189-5D1221956DA3; **Location:** country: Panama; locality: Coiba; verbatimLocality: Sendero Los Monos; verbatimCoordinates: 7° 36' 2.891''N 81° 43' 35.187''O; **Event:** eventDate: 26-08-21

##### Distribution

Guatemala, Panama, Colombia, Brazil

##### Notes

GU, PA-SA

#### Xanthofreya arraijanica

(Chickering, 1946)

610D5A69-1307-528C-944E-FD464D4E7BAE

##### Materials

- **Type status:** Other material. **Occurrence:** recordedBy: Daniel Murcia & Dumas Galvez; sex: 1 male; occurrenceID: DDA077EA-202F-564D-9F20-2832AA249E10; **Location:** country: Panama; locality: Coiba; verbatimLocality: Sendero Los Monos; verbatimCoordinates: 7° 36' 2.891''N 81° 43' 35.187''O; **Event:** eventDate: 18-10-22

##### Distribution

Panama, Colombia

##### Notes

PA-CO

#### Scytodes cf. intricata

162EB104-01EF-596B-9169-6B3521754382

##### Materials

- **Type status:** Other material. **Occurrence:** recordedBy: Daniel Murcia & Dumas Galvez; sex: 1 female, 1 male, 1 immature; occurrenceID: 8F1C3C78-FB14-589E-A340-210D1CA82968; **Location:** country: Panama; locality: Coiba; verbatimLocality: Sendero de Coiba AIP; verbatimCoordinates: 7° 36' 4.903''N 81° 43' 29.06''O; **Event:** eventDate: 6-12-22
- **Type status:** Other material. **Occurrence:** recordedBy: Daniel Murcia & Dumas Galvez; sex: 1 male, 1 immature; occurrenceID: 89A06E5F-8665-52CD-A7ED-F031CB4AAC12; **Location:** country: Panama; locality: Coiba; verbatimLocality: Sendero de Coiba AIP; verbatimCoordinates: 7° 36' 4.903''N 81° 43' 29.06''O; **Event:** eventDate: 7-12-22
- **Type status:** Other material. **Occurrence:** recordedBy: Daniel Murcia & Dumas Galvez; sex: 1 female; occurrenceID: 576E6C64-CA13-5530-904B-B41D6547C286; **Location:** country: Panama; locality: Coiba; verbatimLocality: Sendero Los Monos; verbatimCoordinates: 7° 36' 2.891''N 81° 43' 35.187''O; **Event:** eventDate: 8-12-22
- **Type status:** Other material. **Occurrence:** recordedBy: Daniel Murcia & Dumas Galvez; sex: 1 immature; occurrenceID: 44AA8AB2-2DAA-5702-A98B-E56A3E549951; **Location:** country: Panama; locality: Coiba; verbatimLocality: Antigua Carcel Principal; verbatimCoordinates: 7° 30' 25.049''N 81° 42' 5.065''O; **Event:** eventDate: 25-08-21
- **Type status:** Other material. **Occurrence:** recordedBy: Daniel Murcia & Dumas Galvez; sex: 1 immature; occurrenceID: 4038DA7D-5759-5EE2-A23A-6570F53106E1; **Location:** country: Panama; locality: Coiba; verbatimLocality: Est. MiAmbiente Principal; verbatimCoordinates: 7° 37' 37.024''N 81° 43' 46.56''O; **Event:** eventDate: 29-01-22
- **Type status:** Other material. **Occurrence:** recordedBy: Daniel Murcia & Dumas Galvez; sex: 1 immature; occurrenceID: 4F1AC5B2-775E-5FE9-8479-4A7AFFEC0B05; **Location:** country: Panama; locality: Coiba; verbatimLocality: Sendero Los Monos; verbatimCoordinates: 7° 36' 2.891''N 81° 43' 35.187''O; **Event:** eventDate: 18-10-22
- **Type status:** Other material. **Occurrence:** recordedBy: Daniel Murcia & Dumas Galvez; sex: 1 female, 1 male; occurrenceID: 3941392A-C5DE-5310-9612-51F9EA51FA20; **Location:** country: Panama; locality: Coiba; verbatimLocality: Sendero Los Monos; verbatimCoordinates: 7° 36' 2.891''N 81° 43' 35.187''O; **Event:** eventDate: 19-10-22
- **Type status:** Other material. **Occurrence:** recordedBy: Daniel Murcia & Dumas Galvez; sex: 1 immature; occurrenceID: F9A5E908-19F9-54FA-8269-535FE9CCA98A; **Location:** country: Panama; locality: Coiba; verbatimLocality: Sendero Los Monos; verbatimCoordinates: 7° 36' 2.891''N 81° 43' 35.187''O; **Event:** eventDate: 29-01-22
- **Type status:** Other material. **Occurrence:** recordedBy: Daniel Murcia & Dumas Galvez; sex: 1 immature; occurrenceID: 94B1D00E-35C5-5CA0-A7D3-19B82015BDE0; **Location:** country: Panama; locality: Coiba; verbatimLocality: Sendero Los Monos; verbatimCoordinates: 7° 36' 2.891''N 81° 43' 35.187''O; **Event:** eventDate: 7-02-23

#### Scytodes fusca

Walckenaer, 1837

A8C6BCD9-0BDB-5BB6-9441-A86FF1B7AD32

##### Materials

- **Type status:** Other material. **Occurrence:** recordedBy: Daniel Murcia & Dumas Galvez; sex: 1 female; occurrenceID: 3F6CAFB9-0AD4-52CC-A90E-D5D70C5C2C4B; **Location:** country: Panama; locality: Coiba; verbatimLocality: Sendero de Coiba AIP; verbatimCoordinates: 7° 36' 4.903''N 81° 43' 29.06''O; **Event:** eventDate: 6-12-22
- **Type status:** Other material. **Occurrence:** recordedBy: Daniel Murcia & Dumas Galvez; sex: 1 female; occurrenceID: B343063C-CF89-5FC8-A0F5-9F2D20DE994E; **Location:** country: Panama; locality: Coiba; verbatimLocality: Isla Canales Afuera; verbatimCoordinates: 7° 41' 15.77''N 81° 37' 47.539''O; **Event:** eventDate: 27-08-21
- **Type status:** Other material. **Occurrence:** recordedBy: Daniel Murcia & Dumas Galvez; sex: 1 male, 3 immature; occurrenceID: 6CE71C29-1E13-5824-9969-B04601F4F6A4; **Location:** country: Panama; locality: Coiba; verbatimLocality: Isla Rancheria; verbatimCoordinates: 7° 38' 14.867''N 81° 42' 10.497''O; **Event:** eventDate: 24-03-22
- **Type status:** Other material. **Occurrence:** recordedBy: Daniel Murcia & Dumas Galvez; sex: 1 male, 1 immature; occurrenceID: 21A890F8-C926-5916-8C61-9B4DBCB04B0A; **Location:** country: Panama; locality: Coiba; verbatimLocality: Sendero de Coiba AIP; verbatimCoordinates: 7° 36' 4.903''N 81° 43' 29.06''O; **Event:** eventDate: 24-03-22
- **Type status:** Other material. **Occurrence:** recordedBy: Daniel Murcia & Dumas Galvez; sex: 1 female; occurrenceID: BCA950B5-CFF1-5FD1-A05F-6D4F406476A0; **Location:** country: Panama; locality: Coiba; verbatimLocality: Sendero Los Monos; verbatimCoordinates: 7° 36' 2.891''N 81° 43' 35.187''O; **Event:** eventDate: 19-10-22

##### Distribution

Northern to Southern America. Introduced to Europe, Africa, Seychelles, India, Myanmar, China, Japan, Hawaii

##### Notes

NA-SA

#### Scytodes sp. 1

09A01AC7-BE1C-5831-8C1F-496D76A8292B

##### Materials

- **Type status:** Other material. **Occurrence:** recordedBy: Daniel Murcia & Dumas Galvez; sex: 1 immature; occurrenceID: FFC25A87-9ACE-5611-BFF9-B85DB4A7B11F; **Location:** country: Panama; locality: Coiba; verbatimLocality: Playa Hermosa; verbatimCoordinates: 7° 30' 53.708''N 81° 52' 0.411''O; **Event:** eventDate: 25-03-22

#### Selenops sp. 1

88B4A63B-4C93-5178-A6F1-8BDAC343B3A3

##### Materials

- **Type status:** Other material. **Occurrence:** recordedBy: Daniel Murcia & Dumas Galvez; sex: 1 immature; occurrenceID: C0976EF6-43D7-519A-9865-036418DF2CA0; **Location:** country: Panama; locality: Coiba; verbatimLocality: Est. MiAmbiente Principal; verbatimCoordinates: 7° 37' 37.024''N 81° 43' 46.56''O; **Event:** eventDate: 25-08-21
- **Type status:** Other material. **Occurrence:** recordedBy: Daniel Murcia & Dumas Galvez; sex: 1 immature; occurrenceID: 93299E6E-644C-5FEC-B041-DB15D1FB2510; **Location:** country: Panama; locality: Coiba; verbatimLocality: Sendero Los Monos; verbatimCoordinates: 7° 36' 2.891''N 81° 43' 35.187''O; **Event:** eventDate: 19-10-22

#### Selenops sp. 2

CB3439E5-103B-5B28-A31B-5BCD2DE63764

##### Materials

- **Type status:** Other material. **Occurrence:** recordedBy: Daniel Murcia & Dumas Galvez; sex: 1 immature; occurrenceID: 91F6D440-4292-5D95-B0AB-3A1BE2B42AA5; **Location:** country: Panama; locality: Coiba; verbatimLocality: Playa Hermosa; verbatimCoordinates: 7° 30' 53.708''N 81° 52' 0.411''O; **Event:** eventDate: 25-03-22

#### Senoculus rubicundus

Chickering, 1953

B2571B3C-53E1-5D97-8419-5DC9ED772ED9

##### Materials

- **Type status:** Other material. **Occurrence:** recordedBy: Daniel Murcia & Dumas Galvez; sex: 1 female; occurrenceID: 39D62BE1-3DC8-5306-8591-CD6F64590DE8; **Location:** country: Panama; locality: Coiba; verbatimLocality: Playa Hermosa; verbatimCoordinates: 7° 30' 53.708''N 81° 52' 0.411''O; **Event:** eventDate: 1-07-22
- **Type status:** Other material. **Occurrence:** recordedBy: Daniel Murcia & Dumas Galvez; sex: 1 immature; occurrenceID: 74245696-00B1-528C-AAB0-3568FD2FA5A3; **Location:** country: Panama; locality: Coiba; verbatimLocality: Sendero de Coiba AIP; verbatimCoordinates: 7° 36' 4.903''N 81° 43' 29.06''O; **Event:** eventDate: 7-12-22
- **Type status:** Other material. **Occurrence:** recordedBy: Daniel Murcia & Dumas Galvez; sex: 3 immature; occurrenceID: B497FB19-D3C0-5000-906E-887BEB7D9576; **Location:** country: Panama; locality: Coiba; verbatimLocality: Isla Canales Afuera; verbatimCoordinates: 7° 41' 15.77''N 81° 37' 47.539''O; **Event:** eventDate: 27-08-21
- **Type status:** Other material. **Occurrence:** recordedBy: Daniel Murcia & Dumas Galvez; sex: 2 immature; occurrenceID: 39EF6FE4-80DF-56DF-9DBE-4953B6C04425; **Location:** country: Panama; locality: Coiba; verbatimLocality: Isla Canales Afuera; verbatimCoordinates: 7° 41' 15.77''N 81° 37' 47.539''O; **Event:** eventDate: 30-01-22
- **Type status:** Other material. **Occurrence:** recordedBy: Daniel Murcia & Dumas Galvez; sex: 1 immature; occurrenceID: D94AC782-2AB8-5C96-BB1C-125A6FD28E73; **Location:** country: Panama; locality: Coiba; verbatimLocality: Mirador Gambute; verbatimCoordinates: 7° 37' 41.657''N 81° 43' 59.174''O; **Event:** eventDate: 27-08-21
- **Type status:** Other material. **Occurrence:** recordedBy: Daniel Murcia & Dumas Galvez; sex: 1 immature; occurrenceID: DB3B930B-5268-5CA9-9655-2B62C95C3370; **Location:** country: Panama; locality: Coiba; verbatimLocality: Playa Hermosa; verbatimCoordinates: 7° 30' 53.708''N 81° 52' 0.411''O; **Event:** eventDate: 27-03-22
- **Type status:** Other material. **Occurrence:** recordedBy: Daniel Murcia & Dumas Galvez; sex: 1 immature; occurrenceID: 6F31614E-A0E5-5C0A-8225-DAD5347E6915; **Location:** country: Panama; locality: Coiba; verbatimLocality: Sendero Los Monos; verbatimCoordinates: 7° 36' 2.891''N 81° 43' 35.187''O; **Event:** eventDate: 19-10-22
- **Type status:** Other material. **Occurrence:** recordedBy: Daniel Murcia & Dumas Galvez; sex: 1 immature; occurrenceID: D7D1AB6D-DB9D-5414-A910-EF498A4F85EF; **Location:** country: Panama; locality: Coiba; verbatimLocality: Sendero Los Monos; verbatimCoordinates: 7° 36' 2.891''N 81° 43' 35.187''O; **Event:** eventDate: 29-01-22
- **Type status:** Other material. **Occurrence:** recordedBy: Daniel Murcia & Dumas Galvez; sex: 1 immature; occurrenceID: 2108C484-6B90-500C-8AAC-615D85460417; **Location:** country: Panama; locality: Coiba; verbatimLocality: Sendero Santa Cruz; verbatimCoordinates: 7° 37' 32.47''N 81° 43' 51.632''O; **Event:** eventDate: 28-08-21

##### Distribution

Panama (endemic)

##### Notes

PA

#### Meri sp. 1

4CE5FA67-588C-5242-BCD0-3B9FC5764287

##### Materials

- **Type status:** Other material. **Occurrence:** recordedBy: Daniel Murcia & Dumas Galvez; sex: 1 immature; occurrenceID: 044D18F7-C0EE-53A9-B00B-D13E035BB3A2; **Location:** country: Panama; locality: Coiba; verbatimLocality: Playa Hermosa; verbatimCoordinates: 7° 30' 53.708''N 81° 52' 0.411''O; **Event:** eventDate: 25-03-22

#### Meri sp. 2

C0A034C3-E205-5596-AE20-5D36DF90EBAD

##### Materials

- **Type status:** Other material. **Occurrence:** recordedBy: Daniel Murcia & Dumas Galvez; sex: 1 immature; occurrenceID: 271F2E10-3EBF-53A4-A7B4-54DFEC086B0D; **Location:** country: Panama; locality: Coiba; verbatimLocality: Isla Canales Afuera; verbatimCoordinates: 7° 41' 15.77''N 81° 37' 47.539''O; **Event:** eventDate: 30-01-22
- **Type status:** Other material. **Occurrence:** recordedBy: Daniel Murcia & Dumas Galvez; sex: 2 immature; occurrenceID: 437F1135-0BF4-5973-922E-1206974BE511; **Location:** country: Panama; locality: Coiba; verbatimLocality: Sendero Los Monos; verbatimCoordinates: 7° 36' 2.891''N 81° 43' 35.187''O; **Event:** eventDate: 19-10-22

#### Nolavia sp. 1

D928FC4C-E6FB-594F-974D-EF6598F7B38A

##### Materials

- **Type status:** Other material. **Occurrence:** recordedBy: Daniel Murcia & Dumas Galvez; sex: 1 female; occurrenceID: 7C9C285D-EC17-5F4F-9904-39F890330BF4; **Location:** country: Panama; locality: Coiba; verbatimLocality: Est. MiAmbiente Principal; verbatimCoordinates: 7° 37' 37.024''N 81° 43' 46.56''O; **Event:** eventDate: 29-01-22

#### Nolavia stylifer

(F. O. Pickard-Cambridge, 1900)

2D9A00DD-92FE-5CC7-85C0-AC7F23E408A1

##### Materials

- **Type status:** Other material. **Occurrence:** recordedBy: Daniel Murcia & Dumas Galvez; sex: 1 male; occurrenceID: 53B17A08-602D-5C95-AD7E-15181F209E40; **Location:** country: Panama; locality: Coiba; verbatimLocality: Playa Hermosa; verbatimCoordinates: 7° 30' 53.708''N 81° 52' 0.411''O; **Event:** eventDate: 26-03-22

##### Distribution

Mexico, Brazil

##### Notes

MX, BR

#### Sparianthis chickeringi

(Gertsch, 1941)

78CDA46B-6D8E-54AD-BE96-F3F1D798894D

##### Materials

- **Type status:** Other material. **Occurrence:** recordedBy: Daniel Murcia & Dumas Galvez; sex: 1 immature; occurrenceID: 1086C1D3-912B-58BF-97B3-2078FA0AFA48; **Location:** country: Panama; locality: Coiba; verbatimLocality: Sendero de Coiba AIP; verbatimCoordinates: 7° 36' 4.903''N 81° 43' 29.06''O; **Event:** eventDate: 24-03-22
- **Type status:** Other material. **Occurrence:** recordedBy: Daniel Murcia & Dumas Galvez; sex: 1 female; occurrenceID: A369E71D-7C3A-54EF-89DE-A3D17A05BBC5; **Location:** country: Panama; locality: Coiba; verbatimLocality: Sendero Santa Cruz; verbatimCoordinates: 7° 37' 32.47''N 81° 43' 51.632''O; **Event:** eventDate: 28-08-21

##### Distribution

Panama (endemic)

##### Notes

PA

#### Uaiuara sp.1

34A8D743-3E56-5AF9-AB7A-C605613B7C18

##### Materials

- **Type status:** Other material. **Occurrence:** recordedBy: Daniel Murcia & Dumas Galvez; sex: 2 immature; occurrenceID: 5830AD2B-6812-599A-A92A-F20791A1192C; **Location:** country: Panama; locality: Coiba; verbatimLocality: Sendero Los Monos; verbatimCoordinates: 7° 36' 2.891''N 81° 43' 35.187''O; **Event:** eventDate: 19-10-22

#### Dolichognatha pentagona

(Hentz, 1850)

98EE2F37-1884-57A9-8683-D4D9C7C2263A

##### Materials

- **Type status:** Other material. **Occurrence:** recordedBy: Daniel Murcia & Dumas Galvez; sex: 1 female, 1 male; occurrenceID: C3DFD38F-F91D-53BC-B455-D46D8F3AF9C8; **Location:** country: Panama; locality: Coiba; verbatimLocality: Sendero de Coiba AIP; verbatimCoordinates: 7° 36' 4.903''N 81° 43' 29.06''O; **Event:** eventDate: 6-12-22
- **Type status:** Other material. **Occurrence:** recordedBy: Daniel Murcia & Dumas Galvez; sex: 2 immature; occurrenceID: E3F1B0E2-9B85-56B7-BAC9-98F1EF67E7BD; **Location:** country: Panama; locality: Coiba; verbatimLocality: Sendero Santa Cruz; verbatimCoordinates: 7° 37' 32.47''N 81° 43' 51.632''O; **Event:** eventDate: 28-08-21

##### Distribution

USA to Venezuela

##### Notes

NA-SA

#### Dolichognatha spinosa

(Petrunkevitch, 1939)

04831174-33E8-5BBE-9C72-EF584FBCAD30

##### Materials

- **Type status:** Other material. **Occurrence:** recordedBy: Daniel Murcia & Dumas Galvez; sex: 1 male; occurrenceID: EABBF50E-5464-522B-A6A4-359472D3A74F; **Location:** country: Panama; locality: Coiba; verbatimLocality: Sendero Los Monos; verbatimCoordinates: 7° 36' 2.891''N 81° 43' 35.187''O; **Event:** eventDate: 8-12-22
- **Type status:** Other material. **Occurrence:** recordedBy: Daniel Murcia & Dumas Galvez; sex: 1 male; occurrenceID: CE47FA2A-4E53-5EA1-83A6-144849CA8330; **Location:** country: Panama; locality: Coiba; verbatimLocality: Isla Rancheria; verbatimCoordinates: 7° 38' 14.867''N 81° 42' 10.497''O; **Event:** eventDate: 24-03-22
- **Type status:** Other material. **Occurrence:** recordedBy: Daniel Murcia & Dumas Galvez; sex: 1 male; occurrenceID: F176E1ED-F5EC-5F09-867A-C2F87724B878; **Location:** country: Panama; locality: Coiba; verbatimLocality: Playa Hermosa; verbatimCoordinates: 7° 30' 53.708''N 81° 52' 0.411''O; **Event:** eventDate: 25-03-22
- **Type status:** Other material. **Occurrence:** recordedBy: Daniel Murcia & Dumas Galvez; sex: 2 female, 1 male, 5 immature; occurrenceID: 3E152039-8A7D-5250-AF31-221BCC8838F2; **Location:** country: Panama; locality: Coiba; verbatimLocality: Isla Jicaron; verbatimCoordinates: 7° 17' 16.022''N 81° 46' 25.359''O; **Event:** eventDate: 8-12-23

##### Distribution

Panama (endemic)

##### Notes

PA

#### Leucauge saphes

Chamberlin & Ivie, 1936

CF9AB0AE-3F3B-50CF-8D5D-74FB92C1CCA1

##### Materials

- **Type status:** Other material. **Occurrence:** recordedBy: Daniel Murcia & Dumas Galvez; sex: 1 immature; occurrenceID: EF9F2E96-AAA2-5A29-9F2B-D8936357C8C1; **Location:** country: Panama; locality: Coiba; verbatimLocality: Sendero de Coiba AIP; verbatimCoordinates: 7° 36' 4.903''N 81° 43' 29.06''O; **Event:** eventDate: 6-12-22
- **Type status:** Other material. **Occurrence:** recordedBy: Daniel Murcia & Dumas Galvez; sex: 1 female; occurrenceID: 4EAB7CE6-726C-597B-99D1-134CC6C73487; **Location:** country: Panama; locality: Coiba; verbatimLocality: Sendero Los Monos; verbatimCoordinates: 7° 36' 2.891''N 81° 43' 35.187''O; **Event:** eventDate: 19-10-22

##### Distribution

Panama (endemic)

##### Notes

PA

#### Leucauge sp. 1

B55C1C92-9E9C-5504-B845-0DAEB045771E

##### Materials

- **Type status:** Other material. **Occurrence:** recordedBy: Daniel Murcia & Dumas Galvez; sex: 2 female, 1 immature; occurrenceID: 0CF7C721-C1DA-5D78-8E0B-EFEF4393101B; **Location:** country: Panama; locality: Coiba; verbatimLocality: Isla Jicaron; verbatimCoordinates: 7° 17' 16.022''N 81° 46' 25.359''O; **Event:** eventDate: 8-12-23

#### Leucauge sp. 2

27B72D54-0175-51AD-B2B6-B7EDDFDB04A7

##### Materials

- **Type status:** Other material. **Occurrence:** recordedBy: Daniel Murcia & Dumas Galvez; sex: 1 immature; occurrenceID: 0B04A064-45B7-5BEF-B005-3088FDCE051E; **Location:** country: Panama; locality: Coiba; verbatimLocality: Isla Canales Afuera; verbatimCoordinates: 7° 41' 15.77''N 81° 37' 47.539''O; **Event:** eventDate: 27-08-21

#### Metabus cf. debilis

ED5213DB-3DAE-590E-9525-9CCFDCD14DC8

##### Materials

- **Type status:** Other material. **Occurrence:** recordedBy: Daniel Murcia & Dumas Galvez; sex: 1 male; occurrenceID: AA23AA05-6A46-58C9-B034-29343A79F968; **Location:** country: Panama; locality: Coiba; verbatimLocality: Isla Rancheria; verbatimCoordinates: 7° 38' 14.867''N 81° 42' 10.497''O; **Event:** eventDate: 24-03-22

#### Tetragnatha cambridgei

Roewer, 1942

8CC7B966-2213-5D55-8958-AA79F543FD5C

##### Materials

- **Type status:** Other material. **Occurrence:** recordedBy: Daniel Murcia & Dumas Galvez; sex: 1 male; occurrenceID: 9D93D283-7EA1-5F36-8466-CBF855D4B230; **Location:** country: Panama; locality: Coiba; verbatimLocality: Isla Rancheria; verbatimCoordinates: 7° 38' 14.867''N 81° 42' 10.497''O; **Event:** eventDate: 24-03-22

##### Distribution

Mexico, Central America, Puerto Rico

##### Notes

MX-C-CA

#### Tetragnatha pallida

O. Pickard-Cambridge, 1889

30C5E92A-D6E6-500C-AE82-96343329FA39

##### Materials

- **Type status:** Other material. **Occurrence:** recordedBy: Daniel Murcia & Dumas Galvez; sex: 1 female; occurrenceID: A365160C-6DBC-5B50-9398-3D4D631F670C; **Location:** country: Panama; locality: Coiba; verbatimLocality: Sendero de Coiba AIP; verbatimCoordinates: 7° 36' 4.903''N 81° 43' 29.06''O; **Event:** eventDate: 6-12-22
- **Type status:** Other material. **Occurrence:** recordedBy: Daniel Murcia & Dumas Galvez; sex: 1 female; occurrenceID: A31094C0-42EE-506F-9D26-E977193D0EDE; **Location:** country: Panama; locality: Coiba; verbatimLocality: Est. Coiba AIP Principal; verbatimCoordinates: 7° 36' 0.461''N 81° 43' 27.094''O; **Event:** eventDate: 26-08-21
- **Type status:** Other material. **Occurrence:** recordedBy: Daniel Murcia & Dumas Galvez; sex: 1 male; occurrenceID: 6043ED06-B904-54D3-BED3-DBBB593BD1AB; **Location:** country: Panama; locality: Coiba; verbatimLocality: Est. MiAmbiente Principal; verbatimCoordinates: 7° 37' 37.024''N 81° 43' 46.56''O; **Event:** eventDate: 16-09-21
- **Type status:** Other material. **Occurrence:** recordedBy: Daniel Murcia & Dumas Galvez; sex: 1 female; occurrenceID: E8B97222-1050-5A9A-9900-3B98E86287FB; **Location:** country: Panama; locality: Coiba; verbatimLocality: Est. MiAmbiente Principal; verbatimCoordinates: 7° 37' 37.024''N 81° 43' 46.56''O; **Event:** eventDate: 29-01-22
- **Type status:** Other material. **Occurrence:** recordedBy: Daniel Murcia & Dumas Galvez; sex: 1 immature; occurrenceID: 87FB6AD8-CB7B-5584-BCFC-E6DAE9AF5585; **Location:** country: Panama; locality: Coiba; verbatimLocality: Isla Canales Afuera; verbatimCoordinates: 7° 41' 15.77''N 81° 37' 47.539''O; **Event:** eventDate: 30-01-22
- **Type status:** Other material. **Occurrence:** recordedBy: Daniel Murcia & Dumas Galvez; sex: 1 male; occurrenceID: 5D5176A6-AA2D-510C-A985-A9024799F457; **Location:** country: Panama; locality: Coiba; verbatimLocality: Isla Rancheria; verbatimCoordinates: 7° 38' 14.867''N 81° 42' 10.497''O; **Event:** eventDate: 24-03-22
- **Type status:** Other material. **Occurrence:** recordedBy: Daniel Murcia & Dumas Galvez; sex: 2 female, 1 male; occurrenceID: 2BAE982B-8CAC-55FB-B757-3F21F2590987; **Location:** country: Panama; locality: Coiba; verbatimLocality: Playa Hermosa; verbatimCoordinates: 7° 30' 53.708''N 81° 52' 0.411''O; **Event:** eventDate: 25-03-22
- **Type status:** Other material. **Occurrence:** recordedBy: Daniel Murcia & Dumas Galvez; sex: 1 female; occurrenceID: 3445E298-AB72-5E74-99D1-233538EFC271; **Location:** country: Panama; locality: Coiba; verbatimLocality: Playa Hermosa; verbatimCoordinates: 7° 30' 53.708''N 81° 52' 0.411''O; **Event:** eventDate: 26-03-22
- **Type status:** Other material. **Occurrence:** recordedBy: Daniel Murcia & Dumas Galvez; sex: 1 female; occurrenceID: 3CC58CE4-E3FE-57E8-9B81-87BE5622B578; **Location:** country: Panama; locality: Coiba; verbatimLocality: Sendero de Coiba AIP; verbatimCoordinates: 7° 36' 4.903''N 81° 43' 29.06''O; **Event:** eventDate: 24-03-22
- **Type status:** Other material. **Occurrence:** recordedBy: Daniel Murcia & Dumas Galvez; sex: 1 female; occurrenceID: 9269A181-DE26-5DCF-A631-2FEF3F87A7B9; **Location:** country: Panama; locality: Coiba; verbatimLocality: Sendero Los Monos; verbatimCoordinates: 7° 36' 2.891''N 81° 43' 35.187''O; **Event:** eventDate: 7-02-23
- **Type status:** Other material. **Occurrence:** recordedBy: Daniel Murcia & Dumas Galvez; sex: 1 female; occurrenceID: 10218825-516E-545D-9DBB-FB1B70025996; **Location:** country: Panama; locality: Coiba; verbatimLocality: Sendero Los Monos; verbatimCoordinates: 7° 36' 2.891''N 81° 43' 35.187''O; **Event:** eventDate: 18-10-22
- **Type status:** Other material. **Occurrence:** recordedBy: Daniel Murcia & Dumas Galvez; sex: 2 female; occurrenceID: 352C2A34-1557-54BC-8EAE-0B792931836E; **Location:** country: Panama; locality: Coiba; verbatimLocality: Sendero Los Monos; verbatimCoordinates: 7° 36' 2.891''N 81° 43' 35.187''O; **Event:** eventDate: 8-02-23

##### Distribution

Costa Rica, Panama

##### Notes

CR-PA

#### Tetragnatha sp. 3

18280DE8-E3F1-50C4-8CCB-8AEDEF8A3724

##### Materials

- **Type status:** Other material. **Occurrence:** recordedBy: Daniel Murcia & Dumas Galvez; sex: 1 female; occurrenceID: CC3E0388-9485-5629-8E70-E7441715A2D2; **Location:** country: Panama; locality: Coiba; verbatimLocality: Sendero Los Monos; verbatimCoordinates: 7° 36' 2.891''N 81° 43' 35.187''O; **Event:** eventDate: 7-02-23
- **Type status:** Other material. **Occurrence:** recordedBy: Daniel Murcia & Dumas Galvez; sex: 4 female; occurrenceID: 2834AF5D-F875-593A-8E51-DAA201DDE316; **Location:** country: Panama; locality: Coiba; verbatimLocality: Isla Canales Afuera; verbatimCoordinates: 7° 41' 15.77''N 81° 37' 47.539''O; **Event:** eventDate: 27-08-21
- **Type status:** Other material. **Occurrence:** recordedBy: Daniel Murcia & Dumas Galvez; sex: 1 female; occurrenceID: 62494FD8-3FF0-56F5-8B27-C4C7AD2E4F25; **Location:** country: Panama; locality: Coiba; verbatimLocality: Isla Canales Afuera; verbatimCoordinates: 7° 41' 15.77''N 81° 37' 47.539''O; **Event:** eventDate: 30-01-22
- **Type status:** Other material. **Occurrence:** recordedBy: Daniel Murcia & Dumas Galvez; sex: 1 female; occurrenceID: 82B39054-D60F-51DF-9953-A243214423EE; **Location:** country: Panama; locality: Coiba; verbatimLocality: Playa Hermosa; verbatimCoordinates: 7° 30' 53.708''N 81° 52' 0.411''O; **Event:** eventDate: 25-03-22
- **Type status:** Other material. **Occurrence:** recordedBy: Daniel Murcia & Dumas Galvez; sex: 1 female, 1 male; occurrenceID: A59BF4A6-B17E-592A-B6B2-81128944BD6D; **Location:** country: Panama; locality: Coiba; verbatimLocality: Sendero Los Monos; verbatimCoordinates: 7° 36' 2.891''N 81° 43' 35.187''O; **Event:** eventDate: 18-10-22
- **Type status:** Other material. **Occurrence:** recordedBy: Daniel Murcia & Dumas Galvez; sex: 1 immature; occurrenceID: 38A2B839-822A-539F-B439-B644463B5825; **Location:** country: Panama; locality: Coiba; verbatimLocality: Sendero Los Monos; verbatimCoordinates: 7° 36' 2.891''N 81° 43' 35.187''O; **Event:** eventDate: 29-01-22
- **Type status:** Other material. **Occurrence:** recordedBy: Daniel Murcia & Dumas Galvez; sex: 1 male; occurrenceID: E361DBEE-3C4D-5791-B5FD-FCA7FE949D1B; **Location:** country: Panama; locality: Coiba; verbatimLocality: Sendero Los Monos; verbatimCoordinates: 7° 36' 2.891''N 81° 43' 35.187''O; **Event:** eventDate: 8-02-23

##### Notes

MX-CA-C-SA

#### Tetragnatha tenuissima

O. Pickard-Cambridge, 1889

F2568ACF-AC33-51FB-B2D7-2A91D3FC7EAC

##### Materials

- **Type status:** Other material. **Occurrence:** recordedBy: Daniel Murcia & Dumas Galvez; sex: 2 immature; occurrenceID: 24F77944-F93E-5902-84FE-545DCB142629; **Location:** country: Panama; locality: Coiba; verbatimLocality: Sendero Los Monos; verbatimCoordinates: 7° 36' 2.891''N 81° 43' 35.187''O; **Event:** eventDate: 8-12-22
- **Type status:** Other material. **Occurrence:** recordedBy: Daniel Murcia & Dumas Galvez; sex: 1 immature; occurrenceID: 1D88DF1C-B3EB-58EA-A4A7-3534087D92BE; **Location:** country: Panama; locality: Coiba; verbatimLocality: Est. MiAmbiente Principal; verbatimCoordinates: 7° 37' 37.024''N 81° 43' 46.56''O; **Event:** eventDate: 29-01-22
- **Type status:** Other material. **Occurrence:** recordedBy: Daniel Murcia & Dumas Galvez; sex: 1 immature; occurrenceID: B362C341-B8A5-5889-AFDE-78B2EE1EAE96; **Location:** country: Panama; locality: Coiba; verbatimLocality: Isla Canales Afuera; verbatimCoordinates: 7° 41' 15.77''N 81° 37' 47.539''O; **Event:** eventDate: 30-01-22
- **Type status:** Other material. **Occurrence:** recordedBy: Daniel Murcia & Dumas Galvez; sex: 2 immature; occurrenceID: 95654F96-9D17-5C5C-AB81-25025B93BFFB; **Location:** country: Panama; locality: Coiba; verbatimLocality: Playa Hermosa; verbatimCoordinates: 7° 30' 53.708''N 81° 52' 0.411''O; **Event:** eventDate: 25-03-22
- **Type status:** Other material. **Occurrence:** recordedBy: Daniel Murcia & Dumas Galvez; sex: 1 immature; occurrenceID: D9A4039E-1016-5D17-9DEB-EF700CBA9106; **Location:** country: Panama; locality: Coiba; verbatimLocality: Isla Jicaron; verbatimCoordinates: 7° 17' 16.022''N 81° 46' 25.359''O; **Event:** eventDate: 8-12-23

##### Distribution

Mexico, Central America, Caribbean to Brazil, Argentina

#### Sericopelma sp. 1

A5091440-F379-536C-AAB8-1C05C49DD4B7

##### Materials

- **Type status:** Other material. **Occurrence:** recordedBy: Daniel Murcia & Dumas Galvez; sex: 1 female; occurrenceID: E3D73508-F913-538F-90FA-450D77CBFBE1; **Location:** country: Panama; locality: Coiba; verbatimLocality: Sendero de Coiba AIP; verbatimCoordinates: 7° 36' 4.903''N 81° 43' 29.06''O; **Event:** eventDate: 7-12-22

#### Ariamnes attenuatus

O. Pickard-Cambridge, 1881

DE30D814-66F2-55D2-BF47-AFD3DEC1DA22

##### Materials

- **Type status:** Other material. **Occurrence:** recordedBy: Daniel Murcia & Dumas Galvez; sex: 2 female, 1 male; occurrenceID: 6B582BF5-F7BC-59D7-AA76-73881624F86E; **Location:** country: Panama; locality: Coiba; verbatimLocality: Isla Canales Afuera; verbatimCoordinates: 7° 41' 15.77''N 81° 37' 47.539''O; **Event:** eventDate: 30-01-22
- **Type status:** Other material. **Occurrence:** recordedBy: Daniel Murcia & Dumas Galvez; sex: 1 immature; occurrenceID: 2360F224-9548-5575-8F29-55E013439EB1; **Location:** country: Panama; locality: Coiba; verbatimLocality: Playa Hermosa; verbatimCoordinates: 7° 30' 53.708''N 81° 52' 0.411''O; **Event:** eventDate: 25-03-22
- **Type status:** Other material. **Occurrence:** recordedBy: Daniel Murcia & Dumas Galvez; sex: 1 female; occurrenceID: E66BE5CB-4816-5D6D-8539-A22E7F3E561C; **Location:** country: Panama; locality: Coiba; verbatimLocality: Sendero Los Monos; verbatimCoordinates: 7° 36' 2.891''N 81° 43' 35.187''O; **Event:** eventDate: 19-10-22

##### Distribution

Costa Rica, Caribbean to Argentina

##### Notes

CR, C-SA

#### Chrysso albomaculata

O. Pickard-Cambridge, 1882

80683980-69FD-5B84-8C1A-D91469783C68

##### Materials

- **Type status:** Other material. **Occurrence:** recordedBy: Daniel Murcia & Dumas Galvez; sex: 1 female; occurrenceID: A3F8AE92-DA38-593F-8636-50AB052DBF1E; **Location:** country: Panama; locality: Coiba; verbatimLocality: Sendero Los Monos; verbatimCoordinates: 7° 36' 2.891''N 81° 43' 35.187''O; **Event:** eventDate: 7-02-23

##### Distribution

USA, Mexico to Brazil, Caribbean

##### Notes

NA-C-SA

#### Chrysso cf. vallensis

9D9BBA8C-A291-5BCC-8728-C31E409B7DA5

##### Materials

- **Type status:** Other material. **Occurrence:** recordedBy: Daniel Murcia & Dumas Galvez; sex: 1 male; occurrenceID: 24BE7D2C-D90B-593D-9A81-99E3B4955A5A; **Location:** country: Panama; locality: Coiba; verbatimLocality: Playa Hermosa; verbatimCoordinates: 7° 30' 53.708''N 81° 52' 0.411''O; **Event:** eventDate: 25-03-22
- **Type status:** Other material. **Occurrence:** recordedBy: Daniel Murcia & Dumas Galvez; sex: 1 female; occurrenceID: 8032D16E-044A-511A-9349-E88900DFB7A0; **Location:** country: Panama; locality: Coiba; verbatimLocality: Sendero Los Monos; verbatimCoordinates: 7° 36' 2.891''N 81° 43' 35.187''O; **Event:** eventDate: 18-10-22
- **Type status:** Other material. **Occurrence:** recordedBy: Daniel Murcia & Dumas Galvez; sex: 1 female; occurrenceID: D1D6B54B-0C7D-5405-B8AC-4837DA313485; **Location:** country: Panama; locality: Coiba; verbatimLocality: Sendero Los Monos; verbatimCoordinates: 7° 36' 2.891''N 81° 43' 35.187''O; **Event:** eventDate: 29-01-22

#### Cryptachaea cf. taeniata

CA6D7FA1-5DB3-5D17-8F67-F19152436588

##### Materials

- **Type status:** Other material. **Occurrence:** recordedBy: Daniel Murcia & Dumas Galvez; sex: 3 female, 1 immature; occurrenceID: E65A72F5-FB79-5044-AF2C-5BD5C1EEC946; **Location:** country: Panama; locality: Coiba; verbatimLocality: Playa Hermosa; verbatimCoordinates: 7° 30' 53.708''N 81° 52' 0.411''O; **Event:** eventDate: 1-07-22
- **Type status:** Other material. **Occurrence:** recordedBy: Daniel Murcia & Dumas Galvez; sex: 1 female; occurrenceID: CB6EF2E1-69F5-5DBE-8336-9E17C67381C5; **Location:** country: Panama; locality: Coiba; verbatimLocality: Sendero de Coiba AIP; verbatimCoordinates: 7° 36' 4.903''N 81° 43' 29.06''O; **Event:** eventDate: 6-12-22
- **Type status:** Other material. **Occurrence:** recordedBy: Daniel Murcia & Dumas Galvez; sex: 2 female, 1 immature; occurrenceID: 0D5D8E97-8B35-5361-A8BF-9ED4B7971128; **Location:** country: Panama; locality: Coiba; verbatimLocality: Sendero Santa Cruz; verbatimCoordinates: 7° 37' 32.47''N 81° 43' 51.632''O; **Event:** eventDate: 28-08-21

#### Dipoena sp. 1

553B756D-CD35-590D-AD7D-23D6CECF7BF2

##### Materials

- **Type status:** Other material. **Occurrence:** recordedBy: Daniel Murcia & Dumas Galvez; sex: 1 male; occurrenceID: 793569A8-15ED-5425-8A86-D1E3998C2669; **Location:** country: Panama; locality: Coiba; verbatimLocality: Isla Canales Afuera; verbatimCoordinates: 7° 41' 15.77''N 81° 37' 47.539''O; **Event:** eventDate: 27-08-21

#### Dipoena sp. 2

046E1705-B955-568C-8EF5-A040581FE6C5

##### Materials

- **Type status:** Other material. **Occurrence:** recordedBy: Daniel Murcia & Dumas Galvez; sex: 1 female; occurrenceID: 7F6A4308-3A71-5495-B6E7-24C8B1E9DC36; **Location:** country: Panama; locality: Coiba; verbatimLocality: Sendero de Coiba AIP; verbatimCoordinates: 7° 36' 4.903''N 81° 43' 29.06''O; **Event:** eventDate: 7-12-22

#### Emertonella cf. taczanowskii

ABB849F4-09F3-5ABF-8274-B6A81833A217

##### Materials

- **Type status:** Other material. **Occurrence:** recordedBy: Daniel Murcia & Dumas Galvez; sex: 1 male; occurrenceID: B80F152F-BDEC-58C4-B6CC-5D329A78CA2A; **Location:** country: Panama; locality: Coiba; verbatimLocality: Playa Hermosa; verbatimCoordinates: 7° 30' 53.708''N 81° 52' 0.411''O; **Event:** eventDate: 26-03-22

#### Episinus cf. pyrus

11A82B25-39F2-5FFA-8195-BE16D38F14AE

##### Materials

- **Type status:** Other material. **Occurrence:** recordedBy: Daniel Murcia & Dumas Galvez; sex: 1 immature; occurrenceID: 39A5F64E-6456-52D9-A0CA-4869076D7544; **Location:** country: Panama; locality: Coiba; verbatimLocality: Sendero de Coiba AIP; verbatimCoordinates: 7° 36' 4.903''N 81° 43' 29.06''O; **Event:** eventDate: 6-12-22
- **Type status:** Other material. **Occurrence:** recordedBy: Daniel Murcia & Dumas Galvez; sex: 1 immature; occurrenceID: A4BE865F-7A1B-5C0A-A062-DCD0A9B51F61; **Location:** country: Panama; locality: Coiba; verbatimLocality: Sendero Los Monos; verbatimCoordinates: 7° 36' 2.891''N 81° 43' 35.187''O; **Event:** eventDate: 29-01-22
- **Type status:** Other material. **Occurrence:** recordedBy: Daniel Murcia & Dumas Galvez; sex: 1 immature; occurrenceID: A0C672EA-96EC-5254-84B5-7AE79CAE6F6D; **Location:** country: Panama; locality: Coiba; verbatimLocality: Sendero Santa Cruz; verbatimCoordinates: 7° 37' 32.47''N 81° 43' 51.632''O; **Event:** eventDate: 28-08-21

#### Episinus sp. 1

912CFAB6-2B58-5819-A64B-C3F9E98A1CE8

##### Materials

- **Type status:** Other material. **Occurrence:** recordedBy: Daniel Murcia & Dumas Galvez; sex: 1 male; occurrenceID: CF631F79-7719-53A1-AAC5-8F77CFD1F541; **Location:** country: Panama; locality: Coiba; verbatimLocality: Isla Canales Afuera; verbatimCoordinates: 7° 41' 15.77''N 81° 37' 47.539''O; **Event:** eventDate: 30-01-22

#### Episinus sp. 2

9DA0F5ED-EA57-555F-970D-6B47651D9295

##### Materials

- **Type status:** Other material. **Occurrence:** recordedBy: Daniel Murcia & Dumas Galvez; sex: 1 female; occurrenceID: 2DD3F7D9-50EA-5598-B70C-6536D1D6F963; **Location:** country: Panama; locality: Coiba; verbatimLocality: Sendero Los Monos; verbatimCoordinates: 7° 36' 2.891''N 81° 43' 35.187''O; **Event:** eventDate: 29-01-22
- **Type status:** Other material. **Occurrence:** recordedBy: Daniel Murcia & Dumas Galvez; sex: 2 male; occurrenceID: F7334640-0674-587B-8C6D-4A2E4807CAF1; **Location:** country: Panama; locality: Coiba; verbatimLocality: Sendero Los Monos; verbatimCoordinates: 7° 36' 2.891''N 81° 43' 35.187''O; **Event:** eventDate: 18-10-22

#### Hentziectypus sp. 1

23CFC5A0-71DE-530B-9AFF-F2D5C853322D

##### Materials

- **Type status:** Other material. **Occurrence:** recordedBy: Daniel Murcia & Dumas Galvez; sex: 1 female; occurrenceID: 9ACABE36-98F4-57E8-B880-479A428A0643; **Location:** country: Panama; locality: Coiba; verbatimLocality: Est. MiAmbiente Principal; verbatimCoordinates: 7° 37' 37.024''N 81° 43' 46.56''O; **Event:** eventDate: 25-08-21

#### Neopisinus bruneoviridis

(Mello-Leitão, 1948)

102375BB-C9F8-5E82-A82E-1D8C54E7C45D

##### Materials

- **Type status:** Other material. **Occurrence:** recordedBy: Daniel Murcia & Dumas Galvez; sex: 1 female; occurrenceID: B0A6C714-8B44-5376-8D58-89A9856F53A5; **Location:** country: Panama; locality: Coiba; verbatimLocality: Playa Hermosa; verbatimCoordinates: 7° 30' 53.708''N 81° 52' 0.411''O; **Event:** eventDate: 1-07-22
- **Type status:** Other material. **Occurrence:** recordedBy: Daniel Murcia & Dumas Galvez; sex: 1 female; occurrenceID: 9CEFC13E-D8A7-5C48-9798-AA3D05F9804D; **Location:** country: Panama; locality: Coiba; verbatimLocality: Sendero Los Monos; verbatimCoordinates: 7° 36' 2.891''N 81° 43' 35.187''O; **Event:** eventDate: 7-02-23
- **Type status:** Other material. **Occurrence:** recordedBy: Daniel Murcia & Dumas Galvez; sex: 1 female; occurrenceID: 1666F5B0-78A5-5ED5-A980-F70DA43565E8; **Location:** country: Panama; locality: Coiba; verbatimLocality: Isla Rancheria; verbatimCoordinates: 7° 38' 14.867''N 81° 42' 10.497''O; **Event:** eventDate: 24-03-22

##### Distribution

Panama, Trinidad to Brazil

##### Notes

PA, C-BR

#### Neopisinus putus

(O. Pickard-Cambridge, 1894)

1B7B77B8-768E-5150-A521-CDAA4CAD71F9

##### Materials

- **Type status:** Other material. **Occurrence:** recordedBy: Daniel Murcia & Dumas Galvez; sex: 1 female, 2 male; occurrenceID: 06E9C204-A928-560A-A7E6-8B40665EF9D4; **Location:** country: Panama; locality: Coiba; verbatimLocality: Playa Hermosa; verbatimCoordinates: 7° 30' 53.708''N 81° 52' 0.411''O; **Event:** eventDate: 1-07-22
- **Type status:** Other material. **Occurrence:** recordedBy: Daniel Murcia & Dumas Galvez; sex: 1 female, 1 male; occurrenceID: 1285749A-EBD0-5B9C-8751-B7E1708E5A2A; **Location:** country: Panama; locality: Coiba; verbatimLocality: Sendero de Coiba AIP; verbatimCoordinates: 7° 36' 4.903''N 81° 43' 29.06''O; **Event:** eventDate: 6-12-22
- **Type status:** Other material. **Occurrence:** recordedBy: Daniel Murcia & Dumas Galvez; sex: 1 female, 1 immature; occurrenceID: E8406FC0-740A-5140-9C1D-670CBC742AE9; **Location:** country: Panama; locality: Coiba; verbatimLocality: Sendero de Coiba AIP; verbatimCoordinates: 7° 36' 4.903''N 81° 43' 29.06''O; **Event:** eventDate: 7-12-22
- **Type status:** Other material. **Occurrence:** recordedBy: Daniel Murcia & Dumas Galvez; sex: 2 female, 1 male; occurrenceID: 8BDCE78D-A48B-5C8F-A3A5-CA4D884E48A7; **Location:** country: Panama; locality: Coiba; verbatimLocality: Sendero Los Monos; verbatimCoordinates: 7° 36' 2.891''N 81° 43' 35.187''O; **Event:** eventDate: 8-12-22
- **Type status:** Other material. **Occurrence:** recordedBy: Daniel Murcia & Dumas Galvez; sex: 1 male; occurrenceID: B6C1153B-2824-5445-834C-E04F5474944A; **Location:** country: Panama; locality: Coiba; verbatimLocality: Sendero Los Monos; verbatimCoordinates: 7° 36' 2.891''N 81° 43' 35.187''O; **Event:** eventDate: 7-02-23
- **Type status:** Other material. **Occurrence:** recordedBy: Daniel Murcia & Dumas Galvez; sex: 1 immature; occurrenceID: 8E644A4E-9402-54A6-A491-6BD60520322C; **Location:** country: Panama; locality: Coiba; verbatimLocality: Est. MiAmbiente Principal; verbatimCoordinates: 7° 37' 37.024''N 81° 43' 46.56''O; **Event:** eventDate: 29-01-22
- **Type status:** Other material. **Occurrence:** recordedBy: Daniel Murcia & Dumas Galvez; sex: 1 immature; occurrenceID: 60380A1E-5F5E-5872-B8E0-8452E72F748D; **Location:** country: Panama; locality: Coiba; verbatimLocality: Isla Rancheria; verbatimCoordinates: 7° 38' 14.867''N 81° 42' 10.497''O; **Event:** eventDate: 24-03-22
- **Type status:** Other material. **Occurrence:** recordedBy: Daniel Murcia & Dumas Galvez; sex: 1 female, 1 male; occurrenceID: 60D7BDEC-90A6-578A-AFB4-578D9B2753F8; **Location:** country: Panama; locality: Coiba; verbatimLocality: Mirador Alto; verbatimCoordinates: 7° 37' 33.488''N 81° 43' 41.199''O; **Event:** eventDate: 26-08-21
- **Type status:** Other material. **Occurrence:** recordedBy: Daniel Murcia & Dumas Galvez; sex: 4 female; occurrenceID: 0ED57D72-BD82-5C56-A6AF-03A4D3CFCBCC; **Location:** country: Panama; locality: Coiba; verbatimLocality: Playa Hermosa; verbatimCoordinates: 7° 30' 53.708''N 81° 52' 0.411''O; **Event:** eventDate: 25-03-22
- **Type status:** Other material. **Occurrence:** recordedBy: Daniel Murcia & Dumas Galvez; sex: 1 male, 2 immature; occurrenceID: 181297F0-71BB-5B73-835C-4C99B44B3D6E; **Location:** country: Panama; locality: Coiba; verbatimLocality: Playa Hermosa; verbatimCoordinates: 7° 30' 53.708''N 81° 52' 0.411''O; **Event:** eventDate: 26-03-22
- **Type status:** Other material. **Occurrence:** recordedBy: Daniel Murcia & Dumas Galvez; sex: 3 female; occurrenceID: EA01231E-5298-5D69-A58F-FC387F4E4278; **Location:** country: Panama; locality: Coiba; verbatimLocality: Sendero de Coiba AIP; verbatimCoordinates: 7° 36' 4.903''N 81° 43' 29.06''O; **Event:** eventDate: 24-03-22
- **Type status:** Other material. **Occurrence:** recordedBy: Daniel Murcia & Dumas Galvez; sex: 1 male, 3 immature; occurrenceID: 05C07003-D4F4-54B4-9AED-65B589543B01; **Location:** country: Panama; locality: Coiba; verbatimLocality: Sendero Los Monos; verbatimCoordinates: 7° 36' 2.891''N 81° 43' 35.187''O; **Event:** eventDate: 18-10-22
- **Type status:** Other material. **Occurrence:** recordedBy: Daniel Murcia & Dumas Galvez; sex: 3 immature; occurrenceID: A4655CCA-7D68-5513-A3A9-4DB69A19C799; **Location:** country: Panama; locality: Coiba; verbatimLocality: Sendero Los Monos; verbatimCoordinates: 7° 36' 2.891''N 81° 43' 35.187''O; **Event:** eventDate: 19-10-22
- **Type status:** Other material. **Occurrence:** recordedBy: Daniel Murcia & Dumas Galvez; sex: 1 male; occurrenceID: A63D49CC-528A-5955-8E74-3EBA1AD151E8; **Location:** country: Panama; locality: Coiba; verbatimLocality: Sendero Los Monos; verbatimCoordinates: 7° 36' 2.891''N 81° 43' 35.187''O; **Event:** eventDate: 26-08-21
- **Type status:** Other material. **Occurrence:** recordedBy: Daniel Murcia & Dumas Galvez; sex: 1 female; occurrenceID: A0D101CA-9D66-500A-A094-C6101DE6E2C8; **Location:** country: Panama; locality: Coiba; verbatimLocality: Sendero Los Monos; verbatimCoordinates: 7° 36' 2.891''N 81° 43' 35.187''O; **Event:** eventDate: 29-01-22
- **Type status:** Other material. **Occurrence:** recordedBy: Daniel Murcia & Dumas Galvez; sex: 1 immature; occurrenceID: FD8FA0BB-AD80-5C60-91E9-3491805B7A46; **Location:** country: Panama; locality: Coiba; verbatimLocality: Sendero Santa Cruz; verbatimCoordinates: 7° 37' 32.47''N 81° 43' 51.632''O; **Event:** eventDate: 28-08-21

##### Distribution

Mexico to Panama

##### Notes

MX-CA

#### Neospintharus sp. 1

01A931B5-CDC0-5E5D-9338-A8BC5398009A

##### Materials

- **Type status:** Other material. **Occurrence:** recordedBy: Daniel Murcia & Dumas Galvez; sex: 1 male; occurrenceID: 6AEEA4B4-1575-51AF-866F-A69E25394BED; **Location:** country: Panama; locality: Coiba; verbatimLocality: Sendero Los Monos; verbatimCoordinates: 7° 36' 2.891''N 81° 43' 35.187''O; **Event:** eventDate: 29-01-22

#### Phycosoma altum

(Keyserling, 1886)

D2953299-4ADD-5EA1-9019-D6510049C3F4

##### Materials

- **Type status:** Other material. **Occurrence:** recordedBy: Daniel Murcia & Dumas Galvez; sex: 1 male; occurrenceID: 4D6EE176-B7E1-50D4-9CF9-1156F311C86A; **Location:** country: Panama; locality: Coiba; verbatimLocality: Isla Canales Afuera; verbatimCoordinates: 7° 41' 15.77''N 81° 37' 47.539''O; **Event:** eventDate: 27-08-21
- **Type status:** Other material. **Occurrence:** recordedBy: Daniel Murcia & Dumas Galvez; sex: 1 female; occurrenceID: 22881A83-8957-5DCD-8347-5F923CCCBD41; **Location:** country: Panama; locality: Coiba; verbatimLocality: Isla Rancheria; verbatimCoordinates: 7° 38' 14.867''N 81° 42' 10.497''O; **Event:** eventDate: 24-03-22

##### Distribution

Mexico to Brazil. Introduced to Hawaii

##### Notes

MX-BR

#### Rhomphaea cf. projiciens

E5725CF2-75DA-5168-8939-D431D7795DDB

##### Materials

- **Type status:** Other material. **Occurrence:** recordedBy: Daniel Murcia & Dumas Galvez; sex: 1 female; occurrenceID: D66330A6-8895-5284-BC2A-CCA57B375E9C; **Location:** country: Panama; locality: Coiba; verbatimLocality: Mirador Gambute; verbatimCoordinates: 7° 37' 41.657''N 81° 43' 59.174''O; **Event:** eventDate: 27-08-21
- **Type status:** Other material. **Occurrence:** recordedBy: Daniel Murcia & Dumas Galvez; sex: 2 female; occurrenceID: A6222D9B-837F-5B7E-81F1-BCE9E00A2C23; **Location:** country: Panama; locality: Coiba; verbatimLocality: Sendero Los Monos; verbatimCoordinates: 7° 36' 2.891''N 81° 43' 35.187''O; **Event:** eventDate: 8-02-23

#### Rhomphaea metaltissima

Soares & Camargo, 1948

3D998A15-9EC0-53CD-8828-C79BDA1BE025

##### Materials

- **Type status:** Other material. **Occurrence:** recordedBy: Daniel Murcia & Dumas Galvez; sex: 1 female; occurrenceID: 471E087D-282C-54DB-B66B-62EE196E89E1; **Location:** country: Panama; locality: Coiba; verbatimLocality: Mirador Alto; verbatimCoordinates: 7° 37' 33.488''N 81° 43' 41.199''O; **Event:** eventDate: 26-08-21

##### Distribution

Panama to Brazil

##### Notes

PA-BR

#### Rhomphaea paradoxa

(Taczanowski, 1873)

33222922-CC68-5714-88B9-9832AF5A664D

##### Materials

- **Type status:** Other material. **Occurrence:** recordedBy: Daniel Murcia & Dumas Galvez; sex: 1 female, 1 male; occurrenceID: 0727D213-2540-5544-99D0-92B662F7CABB; **Location:** country: Panama; locality: Coiba; verbatimLocality: Sendero Los Monos; verbatimCoordinates: 7° 36' 2.891''N 81° 43' 35.187''O; **Event:** eventDate: 7-02-23
- **Type status:** Other material. **Occurrence:** recordedBy: Daniel Murcia & Dumas Galvez; sex: 1 female; occurrenceID: A73F4FF3-D6FD-5CCE-8EC9-6AB3C5B38B05; **Location:** country: Panama; locality: Coiba; verbatimLocality: Sendero de Coiba AIP; verbatimCoordinates: 7° 36' 4.903''N 81° 43' 29.06''O; **Event:** eventDate: 7-06-23
- **Type status:** Other material. **Occurrence:** recordedBy: Daniel Murcia & Dumas Galvez; sex: 1 immature; occurrenceID: D715B5F0-A158-5C2F-B705-0E3D5F6E38DF; **Location:** country: Panama; locality: Coiba; verbatimLocality: Antigua Carcel Principal; verbatimCoordinates: 7° 30' 25.049''N 81° 42' 5.065''O; **Event:** eventDate: 25-08-21
- **Type status:** Other material. **Occurrence:** recordedBy: Daniel Murcia & Dumas Galvez; sex: 1 male; occurrenceID: 06860BAE-493B-5945-A50C-0CE991B7E9AB; **Location:** country: Panama; locality: Coiba; verbatimLocality: Mirador Alto; verbatimCoordinates: 7° 37' 33.488''N 81° 43' 41.199''O; **Event:** eventDate: 26-08-21
- **Type status:** Other material. **Occurrence:** recordedBy: Daniel Murcia & Dumas Galvez; sex: 1 male; occurrenceID: 512C407E-C8E9-5B40-BD94-A51192296D17; **Location:** country: Panama; locality: Coiba; verbatimLocality: Sendero Santa Cruz; verbatimCoordinates: 7° 37' 32.47''N 81° 43' 51.632''O; **Event:** eventDate: 28-08-21

##### Distribution

St. Vincent, Mexico to Brazil

##### Notes

MX-B, C

#### Theridion sp. 1

B50EC692-F1A4-52CE-B86B-180C21F67720

##### Materials

- **Type status:** Other material. **Occurrence:** recordedBy: Daniel Murcia & Dumas Galvez; sex: 1 female; occurrenceID: 337A9D0A-8A5F-5EB5-8BE5-343A030F8C3C; **Location:** country: Panama; locality: Coiba; verbatimLocality: Isla Canales Afuera; verbatimCoordinates: 7° 41' 15.77''N 81° 37' 47.539''O; **Event:** eventDate: 30-01-22

#### Thwaitesia affinis

O. Pickard-Cambridge, 1882

9074798B-F0B2-508B-AEEE-703A50857789

##### Materials

- **Type status:** Other material. **Occurrence:** recordedBy: Daniel Murcia & Dumas Galvez; sex: 1 immature; occurrenceID: 247FE1D5-DF9D-51BE-9EB5-37F2527648C9; **Location:** country: Panama; locality: Coiba; verbatimLocality: Est. MiAmbiente Principal; verbatimCoordinates: 7° 37' 37.024''N 81° 43' 46.56''O; **Event:** eventDate: 29-01-22
- **Type status:** Other material. **Occurrence:** recordedBy: Daniel Murcia & Dumas Galvez; sex: 1 male; occurrenceID: 212A65B8-C944-5DCE-AEAE-2EE2567F5FEF; **Location:** country: Panama; locality: Coiba; verbatimLocality: Mirador Alto; verbatimCoordinates: 7° 37' 33.488''N 81° 43' 41.199''O; **Event:** eventDate: 26-08-21
- **Type status:** Other material. **Occurrence:** recordedBy: Daniel Murcia & Dumas Galvez; sex: 1 immature; occurrenceID: B85F2C2F-B843-550F-B353-50555D063843; **Location:** country: Panama; locality: Coiba; verbatimLocality: Playa Hermosa; verbatimCoordinates: 7° 30' 53.708''N 81° 52' 0.411''O; **Event:** eventDate: 25-03-22
- **Type status:** Other material. **Occurrence:** recordedBy: Daniel Murcia & Dumas Galvez; sex: 1 female; occurrenceID: 227F2F9A-3489-576D-A46A-DE95452B41C5; **Location:** country: Panama; locality: Coiba; verbatimLocality: Playa Hermosa; verbatimCoordinates: 7° 30' 53.708''N 81° 52' 0.411''O; **Event:** eventDate: 26-03-22
- **Type status:** Other material. **Occurrence:** recordedBy: Daniel Murcia & Dumas Galvez; sex: 1 female; occurrenceID: A58ECF7E-15B5-554D-81DB-1716266499B4; **Location:** country: Panama; locality: Coiba; verbatimLocality: Sendero Los Monos; verbatimCoordinates: 7° 36' 2.891''N 81° 43' 35.187''O; **Event:** eventDate: 16-09-21
- **Type status:** Other material. **Occurrence:** recordedBy: Daniel Murcia & Dumas Galvez; sex: 1 immature; occurrenceID: 61D36148-574A-55A6-B38F-45787EFFB870; **Location:** country: Panama; locality: Coiba; verbatimLocality: Sendero Los Monos; verbatimCoordinates: 7° 36' 2.891''N 81° 43' 35.187''O; **Event:** eventDate: 18-10-22
- **Type status:** Other material. **Occurrence:** recordedBy: Daniel Murcia & Dumas Galvez; sex: 1 female; occurrenceID: 9362E237-8DC4-5979-93C1-99A2197A9611; **Location:** country: Panama; locality: Coiba; verbatimLocality: Sendero Los Monos; verbatimCoordinates: 7° 36' 2.891''N 81° 43' 35.187''O; **Event:** eventDate: 19-10-22
- **Type status:** Other material. **Occurrence:** recordedBy: Daniel Murcia & Dumas Galvez; sex: 1 male, 2 immature; occurrenceID: 3EC09468-DE5F-5176-ACA8-4F1D919BEC47; **Location:** country: Panama; locality: Coiba; verbatimLocality: Sendero Los Monos; verbatimCoordinates: 7° 36' 2.891''N 81° 43' 35.187''O; **Event:** eventDate: 29-01-22
- **Type status:** Other material. **Occurrence:** recordedBy: Daniel Murcia & Dumas Galvez; sex: 1 female; occurrenceID: 6F9BE165-BE14-5A71-ABE9-6FB69455BD5D; **Location:** country: Panama; locality: Coiba; verbatimLocality: Sendero Santa Cruz; verbatimCoordinates: 7° 37' 32.47''N 81° 43' 51.632''O; **Event:** eventDate: 28-08-21

##### Distribution

Panama to Paraguay

##### Notes

PA-SA

#### Thwaitesia sp. 1

DE5E73F2-AFC5-5A87-8CF4-9E5D24A9D13E

##### Materials

- **Type status:** Other material. **Occurrence:** recordedBy: Daniel Murcia & Dumas Galvez; sex: 1 immature; occurrenceID: 024D3DAF-689E-55DE-BF9A-3DC53E7AE74D; **Location:** country: Panama; locality: Coiba; verbatimLocality: Sendero Los Monos; verbatimCoordinates: 7° 36' 2.891''N 81° 43' 35.187''O; **Event:** eventDate: 8-12-22
- **Type status:** Other material. **Occurrence:** recordedBy: Daniel Murcia & Dumas Galvez; sex: 1 immature; occurrenceID: 324EA92C-8D95-594B-8DA0-EC605D710C68; **Location:** country: Panama; locality: Coiba; verbatimLocality: Isla Canales Afuera; verbatimCoordinates: 7° 41' 15.77''N 81° 37' 47.539''O; **Event:** eventDate: 30-01-22

#### Wamba crispulus

(Simon, 1895)

A1134B8F-171C-5CE3-81DF-275A17478FC2

##### Materials

- **Type status:** Other material. **Occurrence:** recordedBy: Daniel Murcia & Dumas Galvez; sex: 1 female; occurrenceID: 7039FA5C-4EE0-5BC1-AF74-9FACCE9706D9; **Location:** country: Panama; locality: Coiba; verbatimLocality: Isla Rancheria; verbatimCoordinates: 7° 38' 14.867''N 81° 42' 10.497''O; **Event:** eventDate: 24-03-22

##### Distribution

Canada to Brazil, Caribbean

##### Notes

NA-SA, C

#### Epilineutes globosus

(O. Pickard-Cambridge, 1896)

D8436B39-9B58-518C-9667-DDE935B4E40D

##### Materials

- **Type status:** Other material. **Occurrence:** recordedBy: Daniel Murcia & Dumas Galvez; sex: 1 immature; occurrenceID: 23BFE220-5636-5A41-93D8-C34DAED6B53B; **Location:** country: Panama; locality: Coiba; verbatimLocality: Isla Canales Afuera; verbatimCoordinates: 7° 41' 15.77''N 81° 37' 47.539''O; **Event:** eventDate: 27-08-21
- **Type status:** Other material. **Occurrence:** recordedBy: Daniel Murcia & Dumas Galvez; sex: 1 immature; occurrenceID: F4C4DD7F-F6BD-5274-9645-19A6AF8651DD; **Location:** country: Panama; locality: Coiba; verbatimLocality: Isla Canales Afuera; verbatimCoordinates: 7° 41' 15.77''N 81° 37' 47.539''O; **Event:** eventDate: 30-01-22
- **Type status:** Other material. **Occurrence:** recordedBy: Daniel Murcia & Dumas Galvez; sex: 1 immature; occurrenceID: 0EA666CA-8673-5EBB-B322-ACB74A286655; **Location:** country: Panama; locality: Coiba; verbatimLocality: Sendero Los Monos; verbatimCoordinates: 7° 36' 2.891''N 81° 43' 35.187''O; **Event:** eventDate: 26-08-21

##### Distribution

Mexico to Brazil

##### Notes

NA-SA

#### Naatlo sp. 1

0BDA6C60-D720-5796-B39A-0631CD0BDE61

##### Materials

- **Type status:** Other material. **Occurrence:** recordedBy: Daniel Murcia & Dumas Galvez; sex: 1 male; occurrenceID: A85865AD-E74A-54B7-BC27-0F43E65F6843; **Location:** country: Panama; locality: Coiba; verbatimLocality: Sendero Los Monos; verbatimCoordinates: 7° 36' 2.891''N 81° 43' 35.187''O; **Event:** eventDate: 7-02-23

#### Epicadus taczanowskii

(Roewer, 1951)

61DBF584-A96C-5C86-B38E-BDAB7E31BCF4

##### Materials

- **Type status:** Other material. **Occurrence:** recordedBy: Daniel Murcia & Dumas Galvez; sex: 1 male; occurrenceID: 973D8114-0608-555C-B7C2-290567E1B96C; **Location:** country: Panama; locality: Coiba; verbatimLocality: Sendero Los Monos; verbatimCoordinates: 7° 36' 2.891''N 81° 43' 35.187''O; **Event:** eventDate: 29-01-22

##### Distribution

Hispaniola, Costa Rica, Panama to Peru, Bolivia, Brazil

##### Notes

C, CR-SA

#### Epicadus tuberculatus

(Petrunkevitch, 1910)

8FF6BBF0-4FA4-548F-8E2E-14ABBE82EED7

##### Materials

- **Type status:** Other material. **Occurrence:** recordedBy: Daniel Murcia & Dumas Galvez; sex: 1 female; occurrenceID: 931C35E1-F41D-52CC-B23E-E5C41F573D6D; **Location:** country: Panama; locality: Coiba; verbatimLocality: Sendero de Coiba AIP; verbatimCoordinates: 7° 36' 4.903''N 81° 43' 29.06''O; **Event:** eventDate: 7-06-23
- **Type status:** Other material. **Occurrence:** recordedBy: Daniel Murcia & Dumas Galvez; sex: 1 male; occurrenceID: 78CA7897-0426-5768-968D-2077C25277D4; **Location:** country: Panama; locality: Coiba; verbatimLocality: Isla Canales Afuera; verbatimCoordinates: 7° 41' 15.77''N 81° 37' 47.539''O; **Event:** eventDate: 30-01-22

##### Distribution

Panama, Ecuador, Peru, Brazil

##### Notes

PA, EC,PE, BR

#### Misumenoides sp. 1

4854F847-8984-5915-AA58-2EF1D4A04700

##### Materials

- **Type status:** Other material. **Occurrence:** recordedBy: Daniel Murcia & Dumas Galvez; sex: 1 immature; occurrenceID: 330078D5-5323-558D-84A2-29F8024D3B49; **Location:** country: Panama; locality: Coiba; verbatimLocality: Mirador Gambute; verbatimCoordinates: 7° 37' 41.657''N 81° 43' 59.174''O; **Event:** eventDate: 27-08-21

#### Misumenoides sp. 2

C5853164-78D8-5AD2-831C-5C5CEF688E0E

##### Materials

- **Type status:** Other material. **Occurrence:** recordedBy: Daniel Murcia & Dumas Galvez; sex: 1 immature; occurrenceID: DDF24F86-DA8B-59DA-BFE6-EAAC9D2C2C1A; **Location:** country: Panama; locality: Coiba; verbatimLocality: Antigua Carcel Principal; verbatimCoordinates: 7° 30' 25.049''N 81° 42' 5.065''O; **Event:** eventDate: 25-08-21

#### Synema sp. 1

43E645CE-1F2D-5CC9-90EF-41F6DE7F1195

##### Materials

- **Type status:** Other material. **Occurrence:** recordedBy: Daniel Murcia & Dumas Galvez; sex: 1 immature; occurrenceID: BE838814-E203-5531-9160-CD79426015A2; **Location:** country: Panama; locality: Coiba; verbatimLocality: San Juan; verbatimCoordinates: 7° 27' 34.902''N 81° 43' 18.613''O; **Event:** eventDate: 26-08-21

#### Tmarus ineptus

O. Pickard-Cambridge, 1892

5F36D083-4B63-5C78-A37B-D4C97C516ACD

##### Materials

- **Type status:** Other material. **Occurrence:** recordedBy: Daniel Murcia & Dumas Galvez; sex: 1 female; occurrenceID: 102247F9-C070-5DAF-A028-B78AFE7F0601; **Location:** country: Panama; locality: Coiba; verbatimLocality: Mirador Gambute; verbatimCoordinates: 7° 37' 41.657''N 81° 43' 59.174''O; **Event:** eventDate: 27-08-21
- **Type status:** Other material. **Occurrence:** recordedBy: Daniel Murcia & Dumas Galvez; sex: 1 female, 1 immature; occurrenceID: D35984B5-D9CF-5586-868D-49898B38B1DD; **Location:** country: Panama; locality: Coiba; verbatimLocality: Playa Hermosa; verbatimCoordinates: 7° 30' 53.708''N 81° 52' 0.411''O; **Event:** eventDate: 25-03-22
- **Type status:** Other material. **Occurrence:** recordedBy: Daniel Murcia & Dumas Galvez; sex: 1 female, 2 immature; occurrenceID: B66FC272-6A71-5CBC-A057-C98F17AE21BD; **Location:** country: Panama; locality: Coiba; verbatimLocality: San Juan; verbatimCoordinates: 7° 27' 34.902''N 81° 43' 18.613''O; **Event:** eventDate: 16-09-21
- **Type status:** Other material. **Occurrence:** recordedBy: Daniel Murcia & Dumas Galvez; sex: 4 female, 1 immature; occurrenceID: C28C0643-2D79-5C64-B118-40B4D085ED9C; **Location:** country: Panama; locality: Coiba; verbatimLocality: San Juan; verbatimCoordinates: 7° 27' 34.902''N 81° 43' 18.613''O; **Event:** eventDate: 26-08-21
- **Type status:** Other material. **Occurrence:** recordedBy: Daniel Murcia & Dumas Galvez; sex: 1 immature; occurrenceID: E3AB3448-95B1-5618-8405-23BA15FCD816; **Location:** country: Panama; locality: Coiba; verbatimLocality: Sendero Los Monos; verbatimCoordinates: 7° 36' 2.891''N 81° 43' 35.187''O; **Event:** eventDate: 29-01-22

##### Distribution

Panama, Colombia

##### Notes

PA, C

#### Tmarus intentus

O. Pickard-Cambridge, 1892

D5C9F63C-E952-5A9A-B934-5A4FD906286F

##### Materials

- **Type status:** Other material. **Occurrence:** recordedBy: Daniel Murcia & Dumas Galvez; sex: 1 female; occurrenceID: 18A24816-B46A-564E-909B-3236ECA9EFBF; **Location:** country: Panama; locality: Coiba; verbatimLocality: Playa Hermosa; verbatimCoordinates: 7° 30' 53.708''N 81° 52' 0.411''O; **Event:** eventDate: 1-07-22

##### Distribution

Guatemala, Panama

##### Notes

GU, PA

#### Tmarus probus

Chickering, 1950

E48C6110-481A-5181-8D89-2806EA7C0F0D

##### Materials

- **Type status:** Other material. **Occurrence:** recordedBy: Daniel Murcia & Dumas Galvez; sex: 1 male; occurrenceID: AF611E31-8BBF-5523-8CA2-B5992EECFF0F; **Location:** country: Panama; locality: Coiba; verbatimLocality: Isla Rancheria; verbatimCoordinates: 7° 38' 14.867''N 81° 42' 10.497''O; **Event:** eventDate: 24-03-22

##### Distribution

Panama (endemic)

##### Notes

PA

#### Tmarus sp. 1

E12D319C-A6F2-527F-A999-CDEE03E2CAE4

##### Materials

- **Type status:** Other material. **Occurrence:** recordedBy: Daniel Murcia & Dumas Galvez; sex: 1 female; occurrenceID: 0D96AABD-D8F9-5BFB-A2ED-C2ABA51A1D55; **Location:** country: Panama; locality: Coiba; verbatimLocality: Sendero de Coiba AIP; verbatimCoordinates: 7° 36' 4.903''N 81° 43' 29.06''O; **Event:** eventDate: 24-03-22

#### Tmarus studiosus

O. Pickard-Cambridge, 1892

C3CC990E-97B3-5EC8-B724-DC581F948CD4

##### Materials

- **Type status:** Other material. **Occurrence:** recordedBy: Daniel Murcia & Dumas Galvez; sex: 1 female; occurrenceID: 1B9E8A6E-F828-576C-9311-1CA32F9B78E4; **Location:** country: Panama; locality: Coiba; verbatimLocality: Sendero de Coiba AIP; verbatimCoordinates: 7° 36' 4.903''N 81° 43' 29.06''O; **Event:** eventDate: 7-12-22
- **Type status:** Other material. **Occurrence:** recordedBy: Daniel Murcia & Dumas Galvez; sex: 1 female; occurrenceID: 9F5E693E-B0A3-5966-A8CC-01F7D6108769; **Location:** country: Panama; locality: Coiba; verbatimLocality: Est. Coiba AIP Principal; verbatimCoordinates: 7° 36' 0.461''N 81° 43' 27.094''O; **Event:** eventDate: 25-08-21

##### Distribution

Panama (endemic)

##### Notes

PA

#### Trachelas sp. 1

A0CD51F3-0096-5494-BD9C-2803C36170A6

##### Materials

- **Type status:** Other material. **Occurrence:** recordedBy: Daniel Murcia & Dumas Galvez; sex: 1 male; occurrenceID: C2EB62FB-55E1-59E6-B0AE-63B287752393; **Location:** country: Panama; locality: Coiba; verbatimLocality: Mirador Alto; verbatimCoordinates: 7° 37' 33.488''N 81° 43' 41.199''O; **Event:** eventDate: 26-08-21
- **Type status:** Other material. **Occurrence:** recordedBy: Daniel Murcia & Dumas Galvez; sex: 1 male; occurrenceID: 339319CA-2AEE-50EF-8435-B262D8BF40D4; **Location:** country: Panama; locality: Coiba; verbatimLocality: Sendero Los Monos; verbatimCoordinates: 7° 36' 2.891''N 81° 43' 35.187''O; **Event:** eventDate: 29-01-22

#### Cupiennius cf. granadensis

96D4152C-45BA-59E2-B222-FB450E315097

##### Materials

- **Type status:** Other material. **Occurrence:** recordedBy: Daniel Murcia & Dumas Galvez; sex: 4 female, 2 male, 2 immature; occurrenceID: 113918C0-B31A-5B74-A099-7BFFC6A18235; **Location:** country: Panama; locality: Coiba; verbatimLocality: Playa Hermosa; verbatimCoordinates: 7° 30' 53.708''N 81° 52' 0.411''O; **Event:** eventDate: 1-07-22
- **Type status:** Other material. **Occurrence:** recordedBy: Daniel Murcia & Dumas Galvez; sex: 1 immature; occurrenceID: 46C2EC2C-3162-5545-8C24-2CB4743D282B; **Location:** country: Panama; locality: Coiba; verbatimLocality: Sendero de Coiba AIP; verbatimCoordinates: 7° 36' 4.903''N 81° 43' 29.06''O; **Event:** eventDate: 7-12-22
- **Type status:** Other material. **Occurrence:** recordedBy: Daniel Murcia & Dumas Galvez; sex: 2 immature; occurrenceID: 3836C441-2BEC-5203-BA9E-035AE4EDFB0B; **Location:** country: Panama; locality: Coiba; verbatimLocality: Est. MiAmbiente Principal; verbatimCoordinates: 7° 37' 37.024''N 81° 43' 46.56''O; **Event:** eventDate: 29-01-22
- **Type status:** Other material. **Occurrence:** recordedBy: Daniel Murcia & Dumas Galvez; sex: 2 female; occurrenceID: E9BCCBB2-F9F6-522E-9C23-877D55D5685C; **Location:** country: Panama; locality: Coiba; verbatimLocality: Playa Hermosa; verbatimCoordinates: 7° 30' 53.708''N 81° 52' 0.411''O; **Event:** eventDate: 25-03-22
- **Type status:** Other material. **Occurrence:** recordedBy: Daniel Murcia & Dumas Galvez; sex: 1 immature; occurrenceID: 2111A3CC-DC03-5F02-94E8-12CDBB309241; **Location:** country: Panama; locality: Coiba; verbatimLocality: Playa Hermosa; verbatimCoordinates: 7° 30' 53.708''N 81° 52' 0.411''O; **Event:** eventDate: 26-03-22
- **Type status:** Other material. **Occurrence:** recordedBy: Daniel Murcia & Dumas Galvez; sex: 1 immature; occurrenceID: 1AEC5D1C-80CF-585C-A33C-ABD7613DC741; **Location:** country: Panama; locality: Coiba; verbatimLocality: Sendero de Coiba AIP; verbatimCoordinates: 7° 36' 4.903''N 81° 43' 29.06''O; **Event:** eventDate: 24-03-22
- **Type status:** Other material. **Occurrence:** recordedBy: Daniel Murcia & Dumas Galvez; sex: 1 immature; occurrenceID: 32229956-6CA0-5CC6-84B2-35B027C12935; **Location:** country: Panama; locality: Coiba; verbatimLocality: Sendero Los Monos; verbatimCoordinates: 7° 36' 2.891''N 81° 43' 35.187''O; **Event:** eventDate: 18-10-22

#### Cupiennius coccineus

F. O. Pickard-Cambridge, 1901

1B1D6029-3F2A-5049-A212-26A81522673E

##### Materials

- **Type status:** Other material. **Occurrence:** recordedBy: Daniel Murcia & Dumas Galvez; sex: 1 immature; occurrenceID: 0EF43589-9034-5FF4-95BC-FFD9BCF2EE1B; **Location:** country: Panama; locality: Coiba; verbatimLocality: Est. MiAmbiente Principal; verbatimCoordinates: 7° 37' 37.024''N 81° 43' 46.56''O; **Event:** eventDate: 29-01-22

##### Distribution

Costa Rica to Colombia

##### Notes

CR-CO

#### Cupiennius sp. 1

F4DCA987-E0D0-56CE-91E0-A982FAC92412

##### Materials

- **Type status:** Other material. **Occurrence:** recordedBy: Daniel Murcia & Dumas Galvez; sex: 1 immature; occurrenceID: D591C79A-F446-526F-A70A-E7698530E7D0; **Location:** country: Panama; locality: Coiba; verbatimLocality: Sendero Los Monos; verbatimCoordinates: 7° 36' 2.891''N 81° 43' 35.187''O; **Event:** eventDate: 18-10-22

#### Trechaela extensa

(O. Pickard-Cambridge, 1896)

3F6AAC8F-0022-5BC8-BB06-C6EC35774438

##### Materials

- **Type status:** Other material. **Occurrence:** recordedBy: Daniel Murcia & Dumas Galvez; sex: 3 immature; occurrenceID: 7527DFFF-AD61-54FD-BB9E-97DAF5FA19FA; **Location:** country: Panama; locality: Coiba; verbatimLocality: Sendero Los Monos; verbatimCoordinates: 7° 36' 2.891''N 81° 43' 35.187''O; **Event:** eventDate: 7-02-23
- **Type status:** Other material. **Occurrence:** recordedBy: Daniel Murcia & Dumas Galvez; sex: 1 male; occurrenceID: 074DC130-9C7E-55B1-AC38-B07DB7CF9A91; **Location:** country: Panama; locality: Coiba; verbatimLocality: Sendero Los Monos; verbatimCoordinates: 7° 36' 2.891''N 81° 43' 35.187''O; **Event:** eventDate: 7-02-23
- **Type status:** Other material. **Occurrence:** recordedBy: Daniel Murcia & Dumas Galvez; sex: 1 immature; occurrenceID: 8969993C-46D6-5FB9-B106-8FED832D2559; **Location:** country: Panama; locality: Coiba; verbatimLocality: Isla Canales Afuera; verbatimCoordinates: 7° 41' 15.77''N 81° 37' 47.539''O; **Event:** eventDate: 30-01-22

##### Distribution

Mexico to Panama

##### Notes

MX-CA

#### Miagrammopes simus

Chamberlin & Ivie, 1936

F044FE73-3F49-5AE5-8647-9B50597395F0

##### Materials

- **Type status:** Other material. **Occurrence:** recordedBy: Daniel Murcia & Dumas Galvez; sex: 1 female; occurrenceID: ACAABC88-57BE-5482-8528-F090FDC9B5BB; **Location:** country: Panama; locality: Coiba; verbatimLocality: Sendero de Coiba AIP; verbatimCoordinates: 7° 36' 4.903''N 81° 43' 29.06''O; **Event:** eventDate: 6-12-22
- **Type status:** Other material. **Occurrence:** recordedBy: Daniel Murcia & Dumas Galvez; sex: 1 male; occurrenceID: BB7B705E-1925-526F-A428-160481A55490; **Location:** country: Panama; locality: Coiba; verbatimLocality: Sendero Los Monos; verbatimCoordinates: 7° 36' 2.891''N 81° 43' 35.187''O; **Event:** eventDate: 8-12-22
- **Type status:** Other material. **Occurrence:** recordedBy: Daniel Murcia & Dumas Galvez; sex: 1 male; occurrenceID: 4157CD89-FF04-5051-9D3F-A22F174BBDA9; **Location:** country: Panama; locality: Coiba; verbatimLocality: Sendero Los Monos; verbatimCoordinates: 7° 36' 2.891''N 81° 43' 35.187''O; **Event:** eventDate: 18-10-22
- **Type status:** Other material. **Occurrence:** recordedBy: Daniel Murcia & Dumas Galvez; sex: 1 male; occurrenceID: A43ECB9B-C39A-5421-B6DD-AED877E8A076; **Location:** country: Panama; locality: Coiba; verbatimLocality: Sendero Los Monos; verbatimCoordinates: 7° 36' 2.891''N 81° 43' 35.187''O; **Event:** eventDate: 19-10-22

##### Notes

PA

#### Miagrammopes sp. 1

13E0FED8-F3C5-5324-A36E-666C93399A8A

##### Materials

- **Type status:** Other material. **Occurrence:** recordedBy: Daniel Murcia & Dumas Galvez; sex: 1 female, 4 immature; occurrenceID: F46F124D-481E-5C07-9FFA-D7D37F915FD0; **Location:** country: Panama; locality: Coiba; verbatimLocality: Sendero Los Monos; verbatimCoordinates: 7° 36' 2.891''N 81° 43' 35.187''O; **Event:** eventDate: 19-10-22
- **Type status:** Other material. **Occurrence:** recordedBy: Daniel Murcia & Dumas Galvez; sex: 1 immature; occurrenceID: 6D261D60-25D8-5CA4-B8B6-2359C7478BE8; **Location:** country: Panama; locality: Coiba; verbatimLocality: Sendero Los Monos; verbatimCoordinates: 7° 36' 2.891''N 81° 43' 35.187''O; **Event:** eventDate: 29-01-22

##### Distribution

Panama (endemic)

#### Philoponella tingens

(Chamberlin & Ivie, 1936)

DFCF5B4F-F723-5F31-8CCE-36DD25D7717B

##### Materials

- **Type status:** Other material. **Occurrence:** recordedBy: Daniel Murcia & Dumas Galvez; sex: 1 female, 1 male; occurrenceID: D83C92C9-E74C-59D0-AF40-7447DF17BEED; **Location:** country: Panama; locality: Coiba; verbatimLocality: Sendero de Coiba AIP; verbatimCoordinates: 7° 36' 4.903''N 81° 43' 29.06''O; **Event:** eventDate: 6-12-22
- **Type status:** Other material. **Occurrence:** recordedBy: Daniel Murcia & Dumas Galvez; sex: 1 female; occurrenceID: 3D3FFCCB-F1E7-561B-8F83-D06B6D1E98B4; **Location:** country: Panama; locality: Coiba; verbatimLocality: Isla Rancheria; verbatimCoordinates: 7° 38' 14.867''N 81° 42' 10.497''O; **Event:** eventDate: 24-03-22
- **Type status:** Other material. **Occurrence:** recordedBy: Daniel Murcia & Dumas Galvez; sex: 1 immature; occurrenceID: BFFB19FF-524A-508A-9764-DB26AEEBC378; **Location:** country: Panama; locality: Coiba; verbatimLocality: Sendero Los Monos; verbatimCoordinates: 7° 36' 2.891''N 81° 43' 35.187''O; **Event:** eventDate: 18-10-22
- **Type status:** Other material. **Occurrence:** recordedBy: Daniel Murcia & Dumas Galvez; sex: 1 female; occurrenceID: B0DF11D1-CF1D-5AFE-A18A-8AD233FA5DC2; **Location:** country: Panama; locality: Coiba; verbatimLocality: Sendero Los Monos; verbatimCoordinates: 7° 36' 2.891''N 81° 43' 35.187''O; **Event:** eventDate: 29-01-22

##### Distribution

Mexico to Colombia

##### Notes

MX-SA

#### Uloborus sp. 1

F0B5C3C9-860B-599D-B0AF-5FB10635CC34

##### Materials

- **Type status:** Other material. **Occurrence:** recordedBy: Daniel Murcia & Dumas Galvez; sex: 1 immature; occurrenceID: 06916930-90F8-561E-861C-F722FE88571B; **Location:** country: Panama; locality: Coiba; verbatimLocality: Est. Coiba AIP Principal; verbatimCoordinates: 7° 36' 0.461''N 81° 43' 27.094''O; **Event:** eventDate: 26-08-21

#### Uloborus trilineatus

Keyserling, 1883

C06D54F2-1827-5EE0-AF6B-E9D5B2AF07A2

##### Materials

- **Type status:** Other material. **Occurrence:** recordedBy: Daniel Murcia & Dumas Galvez; sex: 1 immature; occurrenceID: 6FA209D7-6562-51A1-B7F4-166FF4058ADB; **Location:** country: Panama; locality: Coiba; verbatimLocality: Sendero de Coiba AIP; verbatimCoordinates: 7° 36' 4.903''N 81° 43' 29.06''O; **Event:** eventDate: 6-12-22
- **Type status:** Other material. **Occurrence:** recordedBy: Daniel Murcia & Dumas Galvez; sex: 1 female; occurrenceID: E0CBA598-FE27-5F23-B35D-B528618DAE47; **Location:** country: Panama; locality: Coiba; verbatimLocality: Sendero Los Monos; verbatimCoordinates: 7° 36' 2.891''N 81° 43' 35.187''O; **Event:** eventDate: 7-02-23
- **Type status:** Other material. **Occurrence:** recordedBy: Daniel Murcia & Dumas Galvez; sex: 1 female; occurrenceID: ED18C528-84F5-5333-A6DB-C483DC384969; **Location:** country: Panama; locality: Coiba; verbatimLocality: Sendero Los Monos; verbatimCoordinates: 7° 36' 2.891''N 81° 43' 35.187''O; **Event:** eventDate: 8-02-23
- **Type status:** Other material. **Occurrence:** recordedBy: Daniel Murcia & Dumas Galvez; sex: 1 female; occurrenceID: B2392EA5-E96C-544A-982B-14CFF7D00D8C; **Location:** country: Panama; locality: Coiba; verbatimLocality: Isla Rancheria; verbatimCoordinates: 7° 38' 14.867''N 81° 42' 10.497''O; **Event:** eventDate: 24-03-22
- **Type status:** Other material. **Occurrence:** recordedBy: Daniel Murcia & Dumas Galvez; sex: 2 immature; occurrenceID: D02DF7DF-3B27-5DAF-B0E7-704154A5F338; **Location:** country: Panama; locality: Coiba; verbatimLocality: Sendero Los Monos; verbatimCoordinates: 7° 36' 2.891''N 81° 43' 35.187''O; **Event:** eventDate: 18-10-22
- **Type status:** Other material. **Occurrence:** recordedBy: Daniel Murcia & Dumas Galvez; sex: 1 female immature; occurrenceID: 98682097-002A-52C8-BBAA-A77A1B18F455; **Location:** country: Panama; locality: Coiba; verbatimLocality: Sendero Los Monos; verbatimCoordinates: 7° 36' 2.891''N 81° 43' 35.187''O; **Event:** eventDate: 19-10-22

##### Distribution

Mexico to Argentina

##### Notes

MX-SA

## Analysis

**Figure 2.**
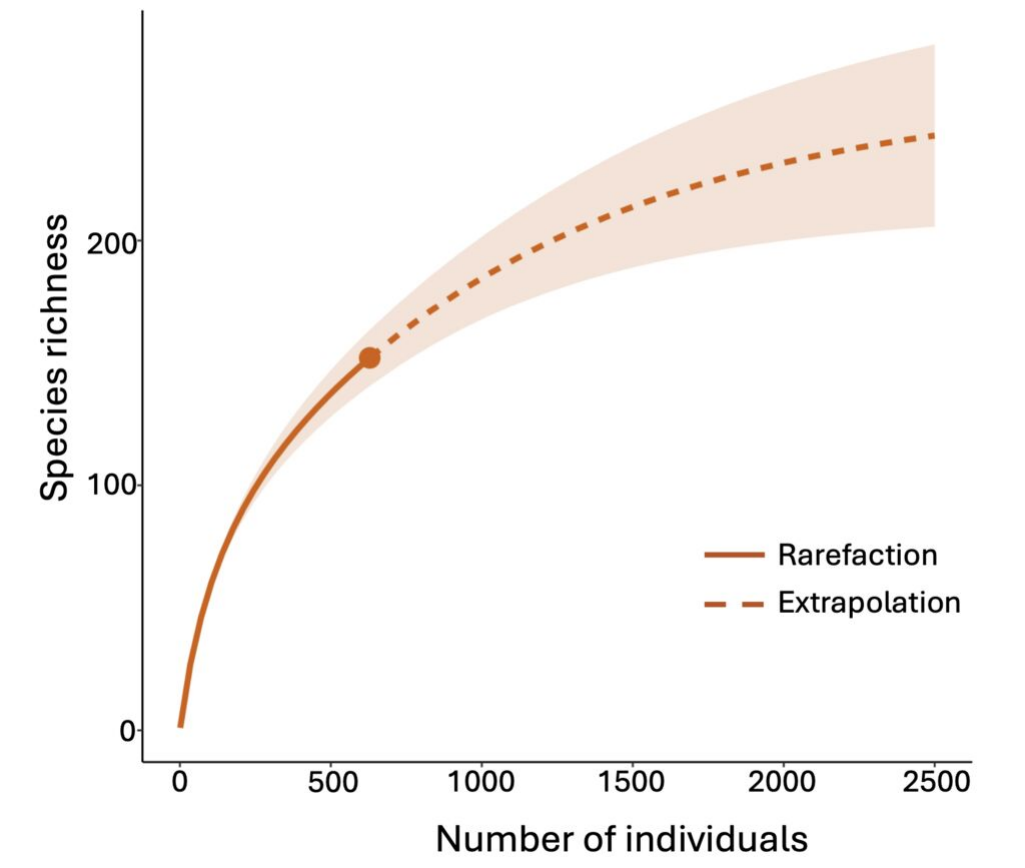
Rarefaction curve showing observed species richness of spiders in Coiba National Park. The dotted line represents the extrapolation of the sampling curve with 95% confidence intervals (shaded area). Sampling was carried out from August 2021 to August 2023 in 13 sites of the Park.

A total of 635 individuals were identified belonging to 152 species from 98 genera in 30 families (Table 2). In terms of species richness per family, we identified 23 species of Araneidae (15%), 22 Theridiidae (14%), 20 Salticidae (13%), 10 Tetragnathidae or Thomisidae (both 7%), seven Ctenidae or Corinnidae (both 5%), six Sparassidae (4%), five Uloboridae or Pholcidae (both 3%), plus four Trechaleidae, three Anyphaenidae, Clubionidae, Lycosidae, Mimetidae, Scytodidae and two Oonopidae, Selenopidae or Theridiosomatidae (all < 2%). We found only one species for the families Caponiidae, Cheiracanthiidae, Cyrtaucheniidae, Filistatidae, Gnaphosidae, Hersiliidae, Oxyopidae, Senoculidae and Trachelidae. These latter families were the least abundant as well, while the families with comparatively more individuals collected were Araneidae (172 specimens, 27%), Theridiidae (81, 13%) Thomisidae (23, 3.6%), Salticidae (65, 10%), Tetragnathidae (56, 8%) and Ctenidae (41, 6%). Overall, our sampling is unlikely to have reported all the species present in the Park and the total number of species could be around 237 species, based on the extrapolation (Fig. 2). Moreover, comparisons between sites still requires more sampling in most places and a systematic use of a more equivalent sampling method in all the sites. For example, site 9 showed the highest richness and abundance, likely due to the higher number of visits as compared to other sites (e.g. 1,7, Suppl. material 2), rather than a true reflection of any richer or more abundant araneofauna. Alternatively, it seems logical that, if given equivalent sampling effort, a forest habitat (e.g. site 1) contains a higher abundance and richness of spiders than a coastal - mangrove habitat (e.g. site 7).

The spiders collected fell into seven functional groups (i.e. guilds, Table 2). The dominant guild was Other Hunters (OH) with 46 species (30%) including the families Salticidae, Ctenidae and Sparassidae. The next most dominant were Orb-Web (OW) with 41 species (27%), followed by Space Webs (CW) with 27 species (18%). Less prevalent were Ground Hunters (GH) with 14 species (9%), then Ambush Hunters (AH) with 12 species (8%). Finally, rare guilds were Specialist (SP) with eight species (5%) and Sensing Web (SW) with four species (3%).

In terms of biogeography, 150 species are of Neotropical origin, with varying ranges of distribution; for example, we can highlight five species in the range NA-CA, twelve species in NA-SA, five species in CA-SA, nine species in MX-SA, seven species in MX-CA, five species in CR-PA and three species in CO-PA, amongst other regional disitributions. The species *Labahithamarginata* (Kishida, 1936) and *Menemerusbivittatus* (Dufour, 1831) have a cosmopolitan distribution (Magalhaes et al. 2022, World Spider Catalog 2023). We found 19 species that are endemic to Panama, but neither of the two endemic species previously documented in the literature for Coiba National Park. In addition, the species *Ctenusnigrolineatus* Berland (1913), *Chapodagitae* Zhang & Maddison, 2012 and *Sarindanigra* Peckham & Peckham 1892 are reported as new country records for Panama (Figs. S1 and S2 in Suppl. material 1). Finally, we found five individuals of Ctenidae that did not match the morphology of any ctenid species reported for the Americas (Genus. 1 sp. 1, Table 2).

## Discussion

Panama contains between 1223 (Nentwig 1993) and 1236 described species of spiders (Murcia-Moreno, unpubl. data), with at least 154 species now recorded for Coiba National Park; 152 in the present study and at least another two in literature (Miranda et al. 2013, Bolzern 2014). Compared to other Pacific Islands, the total number of spider species in Coiba is relatively high. For example, the list of spiders on Easter Island comprises 36 species in a single sampling (Baert et al. 1997). In contrast, Cocos Island comprises 50 species (Víquez 2020), Galapagos Archipelago 159 species (Baert 2013, Baert 2014, Buchholz et al. 2020) and Hawaii Archipelago 168 species (Suman 1964), with different sampling efforts and sampling size (Suman 1964, Baert 2013, Baert 2014, Buchholz et al. 2020, Víquez 2020). Coiba seems to show a predicted higher diversity than Hawaii and Galapagos despite its smaller size and a potential explanation could be its closer proximity to the mainland, which favours higher immigration rates (MacArthur and Wilson 1963).

It is very likely that further work will reveal more species since our rarefaction curve has not yet reached a plateau, which also means that we cannot make meaninful comparisons between islands and sites at this point. Importantly, higher plant diversity, increased complexity of plant architectures and greater prey abundance strongly correlate with higher spider diversity (Greenstone 1984, Theron et al. 2020, Butz et al. 2023). Therefore, the high diversity of spider species in Coiba may be attributed to the high diversity of plants (1200 spp., Ibañez (2011)) and insects (Nieves-Aldrey and Fontal-Cazalla 1997). Barro Colorado, an artificially created island in Central Panama with similar characteristics to Coiba, harbours at least 1300 plant species (Croat 1978) and 1875 species of insects (Smithsonian Tropical Research Institute 2023), which has led to the report of at least 300 spider species (Chamberlin 1925, Banks 1929, Petrunkevitch 1929, Chamberlin and Ivie 1936, Chickering 1936, Chickering 1955, Nentwig 1993). The main island Coiba is approximately 33 times larger than Barro Colorado (15 km^2^) and, therefore, we expect at least the same number of species, despite the conservative estimation of the extrapolation curve (~ 237 spp). Alternatively, the spider diversity in this region may be lower as compared to Central Panama. For instance, a study in the adjacent mainland, in the area of Bahía Honda, found at least 204 spiders species (Quintero A 2005) and 47 of those species were also observed in our study.

Another aspect of utmost importance is the complex interaction of climatic and physical factors, such as high rainfall, humidity and topographic characteristics of Coiba that were not considered during our monitoring. These elements can influence the distribution of the various functional groups within the available microhabitats, because of variation in sites for foraging, shelter or reproduction (Rao 2017). Coiba hosts a wide range of ecological niches in diverse habitats, such as forests, mangroves, shrubs, grasslands, amongst others, which means that different guilds of spiders may be adapting well to these habitats and microhabitats. However, in our study, we monitored mostly sites near the coastline composed of mixed forest, with only one site clearly differing in vegetation composition (location 7, for example, mangrove, herbs), this latter site showing the lowest species richness (9 spp). Future work should be done in the inner parts of the island which would expand our range of microhabitat types and samples sizes to allow meaningful statistical comparisons across sites. A recently installed weather station in Coiba Scientific Station in 2023 can help future studies to obtain accurate estimations of abiotic factors in each habitat and microhabitat type, against which spider population dynamics could be associated.

At the biogeographic level, we have identified species with full distribution in the American continent, Central – South America and North – Central America, in line with the idea that the isthmus of Panama has been a point of encounter and exchange for North and South American spider fauna (Nentwig 1993, Bacon et al. 2015). Nentwig (1993) postulated the Panamanian spider fauna should consist of Central and South American species; however, our results highlight that North American spider fauna also contribute to the overall composition (e.g. NA-CA, MX-CA). Whether some of the allegedly endemic species are truly found only in Panama requires more extensive work in the region. Similarly, species with restricted distribution in Costa Rica – Panama and Colombia – Panama, could suggest that those species were endemic at some point in one of the countries, but some of these are perhaps dispersing more widely across adjacent regions. Overall, the study of invertebrate migrations through the isthmus has received little attention and, for most of the spider species, the dispersal and colonisation processes have not been studied (Nentwig 1993).

It is important to note that, in long-inhabited islands, the presence of human settlements has facilitated the introduction of diverse biota, where it is often unknown whether their arrival occurs naturally or through human intervention (Buchholz et al. 2020, Víquez 2020). In the case of Coiba, there are no settlements, besides the stations from the park rangers and the police. However, the regular visits by tourists to the main island represent an elevated risk for introduction of exotic species. For the cosmopolitan species reported in our dataset, Magalhaes et al. (2022) suggest that the presence of *Labahithamarginata* (Kishida, 1936) on various islands could be due to both natural processes and artificial introductions. To date, this species has been reported from remote Pacific islands, such as Hawaii and the Cook Islands, as well as in regions of continental America extending from Mexico to Brazil. In Panama, this species has been previously reported on the mainland (Magalhaes et al. 2022), but this is the first record of the species for a Panamanian island. In the case of *Menemerusbivittatus*, this species is associated with disturbed environments, often observed in man-made structures (Wesołowska 1999). The first report of this species in the mainland is from before 1901 (Pickard-Cambridge 1901). Future phylogenetic studies of the genus might shed some light into the timing and pattern of colonisation of these species on various islands.

Regarding endemism, Coiba National Park contains a great diversity of fauna and flora, approximately 70 species are considered endemic, including birds, mammals, plants, insects and arachnids (Ridgely and Gwyne 1992, Ibañez et al. 1997, Olson 2007, González et al. 2010, Mendez-Carvajal 2012, Roubik and De Camargo 2012, Miranda et al. 2013, Bolzern 2014, Costas et al. 2015, Daniel and Carrión 2015, Flores et al. 2016, Valdecasas 2021). We identified 18 species of spiders in our data that are endemic to Panama, each newly recorded in Coiba National Park; however, we did not encounter either of the previously reported endemic spiders from only Coiba National Park, namely *Neoctenizaagustinea* Miranda & Arizala, 2013 (Miranda et al. 2013) and *Ponsoonopscoiba* Bolzern, 2014 (Bolzern 2014). *Ctenusnigrolineatus* Berland, 1913 is reported as a new finding for Panama and is based on the fact that the individuals collected match the morphological characteristics evidenced in the literature for females and males (Berland (1913); Silva-Dávila (2003)). However, there is no formal description of the female of this species. The original description made by Berland (1913) was based on male individuals collected in Ecuador. Interestingly, the female that we collected also matches the morphological characteristics of *Ctenusw-notatus* Petrunkevitch 1925, a species endemic to Panama, originally described from only females, without males (Petrunkevitch 1925). In this context, we suspect a possible synonymy between *C.nigrolineatus* and *C.w-notatus*. We are currently working on detailing evidence for formal validation (Berland 1913, Silva-Dávila 2003).

In other island studies, in Galapagos, Buchholz et al. (2020) found a high degree of spider endemism in the Galapagos Islands, with at least 50% of their species as endemics. In comparison, the comparatively low endemism of spiders in Coiba can be explained as the relative recent isolation of the island, around 12,000–18,000 years ago (Fairbanks 1989), as compared to ~ 14 million years of isolation of the Galapagos Archipelago (Werner et al. 1999). However, the fraction of endemic spiders from the Galapagos may be reduced as more studies of allied South American araneofauna are carried out, especially from Ecuador, hence revealing their additional presence on the mainland (Buchholz et al. 2020). Moreover, the five ctenid individuals (= Genus 1 sp. 1) that we could not match with any yet known genus of Ctenidae from the Neotropics seemingly belongs to a new genus currently being studied by a specialist (Hazzi N., comm. pers.). Whether this is a new species can be verified when the species belonging to the genus are officially described. Moreover, we failed to identify 59 species (38%); therefore, it remains unknown at the moment whether they are known species or new species. Further work, including current molecular techniques can provide deeper insights into this uncertainty.

In conclusion, this study provides a taxonomic baseline and shows that Coiba National Park still offers vast possibilities for revealing its complete arachnofauna, even for the relatively well known araneofauna, i.e. spiders. Understanding biodiversity and species distribution is an essential part of conservation programmes. Obtaining this information is useful for the long-term management and effective use of biological resources across Panama and beyond and is important for monitoring and conservation actions, based on distributional, temporal and spatial changes. Establishing a list of species in insular areas - where periodic and long-term research is logistically difficult - compared to continental areas, will be useful for future goals, such as monitoring or conservation assessment. For example, Coiba National Park offers the opportunity to study ecological and evolutionary processes, by comparing spider populations and communities with those in the mainland. Moreover, it is of great importance to incorporate current tools for the evaluation of the local araneofauna, which includes DNA barcoding, monitoring of population and community dynamics, plus use of the newly-installed meteorological station on the Island. For example, DNA barcoding could reveal cryptic species or help identify species when only the juveniles are available (Oh and Lee 2022), while data from the meteorological station can help to understand population and community fluctuations in abundance throughout the year (Pitilin et al. 2019). Finally, this works contributes more widely to the understanding of the less studied Latin American araneofauna as compared to its North American counterpart (Santos et al. 2017).

## Supplementary Material

6A35F628-E18D-55E3-BDEA-443F0672EFE4 10.3897/BDJ.12.e117642.suppl1 Supplementary material 1 Supporting literature and figures Data type References and Figures Brief description References used for spider identification and pictures of the new three species reported for Panama. File: oo_1044037.docx https://binary.pensoft.net/file/1044037 Daniel Murcia & Dumas Gálvez

62852CFA-424A-5583-8FED-23DC93F59656 10.3897/BDJ.12.e117642.suppl2 Supplementary material 2 Table S1. Data type Summary table Brief description Summary of species richness and abundance of spiders per site collected in Coiba National Park, from August 2020 to August 2022. File: oo_1043868.docx https://binary.pensoft.net/file/1043868 Daniel Murcia-Moreno & Dumas Gálvez

## Figures and Tables

**Table 1.** Sampling sites in Coiba National Park.

**Location**	**Coordinates**	**Altitude (m)**	**Habitat type**
1. Old main prison	7°30'16.0488"N, 81°41'50.7084"W	220	Anthropogenic
2. Coiba Scientific Station	7°29'0.3372"N, 81°43'27.1596"W	50	Mixed forest
3. MiAmbiente Station	7°37'37.3224"N, 81°43'46.668"W	70	Anthropogenic
4. Mirador Alto	7°37'34.1364"N, 81°43'39.2952"W	133	Mixed forest
5. Mirador Gambute	7°37'32.7576"N, 81°43'51.7548"W	136	Mixed forest
6. Playa Hermosa	7°31'4.9584"N, 81°51'34.1064"W	20	Mixed forest
7. San Juan	7°29'0.3372"N, 81°47'45.2976"W	0	Coastal beach / Mangrove)
8. Sendero de Coiba AIP	7°29'0.3350"N, 81°47'45.2950"W	80	Mixed forest
9. Sendero Los Monos	7°29'0.3350"N, 81°47'45.2950"W	125	Mixed forest
10. Sendero Santa Cruz	7°37'32.7580"N, 81°43'51.7540"W	134	Mixed forest
11. Canales Afuera Island	7°52'39.6340"N, 81°21'22.1800"W	65	Mixed forest
12. Jicaron Island	7°29'0.335"N, 81°47'45.2950"W	79	Mixed forest
13. Coibita Island	7°38'20.4190"N, 81°42'8.9300"W	121	Mixed forest

**Table 2.** Checklist of spiders in National Park Coiba. Abbreviation for samples: imm = immature. Abbreviations for Guild: AH = Ambush Hunter, CW = Space Web, GH = Ground Hunters, OH = Other Hunters, OW = Orb Web, SP = Specialist and SW = Sensing Web. Affinities: NEO = neotropical, COS = Cosmopolitan, end = endemic and * = new record. Region summarises only the distribution in the American continent: AR = Argentina, BR = Brazil, CA = Central America, C = Caribbean, CO = Colombia, CR = Costa Rica, EC = Ecuador, GT = Guatemala, MX = Mexico, NA = North America, NI = Nicaragua, PA = Panama, PE = PERU, SA = South America. For the location codes, see Table 1. Distributions are based on World Spider Catalogue (2023). All samples are from understorey level with the exception of few individuals collected in the canopy, depicted as ^ca^ with the number next to it indicating the number of individuals sampled.

**Species**	**Samples**	**Guild**	**Location**	**Affinities**	**Distribution**	**Region**
**Fam. Anyphaenidae**		OH				
1. *Macrophyeselongata* Chickering, 1937	1 ♂		6	NEO	Costa Rica, Panama	CR-PA
2. *Wulfilamodestus* Chickering, 1937	1 ♂		8	NEO	Panama (end)	PA
3. *Wulfila* sp. 1	1 ♀		9			
**Fam. Araneidae**		OW				
4. *Acacesiatenella* (L. Koch, 1871)	2 ♂ 2 ♀		6, 10	NEO	Mexico to Brazil, French Guiana, Guyana	MX-SA
5. *Allocyclosabifurca* (McCook, 1887)	1 ♀		11	NEO	USA to Panama, Turks & Caicos, Cuba, Hispaniola	NA-CA-C
6. *Cyclosacaroli* (Hentz, 1850)	9 ♀ 1 imm		2, 6, 8, 9, 12	NEO	USA, Caribbean to Bolivia	NA-C-SA
7. *Eriophoraravilla* (C. L. Koch, 1844)	2 ♀		4, 6	NEO	USA to Brazil	NA-SA
8. *Eustalabifida* F. O. Pickard-Cambridge, 1904	1 ♂ 2 ♀		8, 12	NEO	USA to Panama	NA-CA
9. *Eustalaaffdevia*	5 ♀		6, 12			
10. *Eustalaexigua* Chickering, 1955	1 ♂		6	NEO	Panama (end)	PA
11. *Eustalafuscovittata* (Keyserling, 1864)	1 ♂ 1 ♀		9	NEO	Mexico, Cuba to South America	MX-C-CA
12. *Eustalaguttata* F. O. Pickard-Cambridge, 1904	4 ♂ 5 ♀		1, 3, 5, 6, 8, 9, 13	NEO	Mexico to Brazil	MX-SA
13. *Eustalascutigera* (O. Pickard-Cambridge, 1898)	4 ♂ 10 ♀		8, 9, 12, 13	NEO	Mexico to Panama	MX-CA
14. *Eustalasemifoliata* (O. Pickard-Cambridge, 1899)	2 ♀ 1 imm		4, 9, 11	NEO	Central America	CA
15. *Eustala* sp. 1	1 ♂		8			
16. *Eustala* sp. 2	1 ♀		7			
17. *Eustala* sp. 3	58 imm		3, 4, 6, 7, 8, 9, 10, 12			
18. *Eustalatantula* Chickering, 1955	1 ♂		10	NEO	Panama (end)	
19. *Lariniadirecta* (Hentz, 1847)	1 ♀ 2 imm		7, 9	NEO	USA to Brazil	NA-SA
20. *Metazygiakeyserlingi* Banks, 1929	1 ♂ 4 ♀		9, 12	NEO	Costa Rica, Panama, Colombia, Trinidad	CA-SA
21. *Micrathenahorrida* (Taczanowski, 1873)	1 ♀		12	NEO	Greater Antilles, Mexico to Argentina	MX-C-SA
22. *Parawixiahypocrita* (O. Pickard-Cambridge, 1889)	1 ♀ 9 imm		6, 8, 9, 12	NEO	Guatemala to Brazil	CA-SA
23. *Parawixia* sp. 1	1 ♂		9			
24. *Pronousintus* Levi, 1995	1 ♀ 1 imm		6, 10	NEO	Costa Rica to Brazil	CA-SA
25. *Wagnerianatauricornis* (O. Pickard-Cambridge, 1889)	5 ♂ 19 ♀ 7 imm		6, 8, 9, 10, 11, 12, 13	NEO	USA to Peru	NA-SA
26. *Witicacrassicaudus* (Keyserling, 1865)	2 ♀ 2 imm		9, 11, 12	NEO	Mexico to Peru	MX-SA
**Fam. Caponiidae**		SP				
27. *Nopslargus* Chickering, 1967	1 ♂		8	NEO	Panama (end)	PA
**Fam. Cheiracanthiidae**		OH				
28. *Eutichurusputus* O. Pickard-Cambridge, 1898	1 ♀		4	NEO	Panama, Colombia, Ecuador, Peru, Brazil	PA-SA
**Fam. Clubionidae**		OH				
29. Elavercf.tigrina	1 ♂ 3 ♀		6, 9			
30. *Elaverlutescens* (Schmidt, 1971)	1 ♀		8	NEO	Panama to Brazil	PA-SA
31. *Elaver* sp. 1	1 ♀		9			
32. *Elaver* spp.	11 imm^1ca^		6, 8, 9, 13			
**Fam. Corinnidae**		GH				
33. *Castianeira* sp. 1	1 ♀		9			
34. *Castianeira* sp. 2	1 ♀ 1 imm		6, 13			
35. *Corinnabulbosa* F. O. Pickard-Cambridge, 1899	2 ♂ 1 ♀		8, 9	NEO	Mexico to Panama	MX-CA
36. *Corinna* sp. 1	1 ♀		6			
37. Creugascf.mucronatus	1 ♀		9			
38. *Mazaxspinosa* (Simon, 1898)	1 ♂ 4 ♀ 3 imm		2, 3, 4, 6, 13	NEO	Guatemala, Panama, St. Lucia, St. Vincent	CA-C
39. *Simonestus* sp. 1	1 ♀ 1 imm		9			
**Fam. Ctenidae**		OH				
40. *Acanthoctenuslamarrei* Arizala, Labarque & Polotow, 2021	5 ♂ 3 ♀ 2 imm		8	NEO	Panama (end)	PA
41. *Ancylometesbogotensis* (Keyserling, 1877)	1 ♂ 4 ♀ 3 imm		6, 9	NEO	Honduras to Bolivia	CA-SA
42. *Ctenusnigrolineatus* * Berland, 1913	1 ♂ 3 ♀ 2 imm		8, 9, 12	NEO	Ecuador	PA, EC
43. *Kiekiebarrocolorado* Polotow & Brescovit, 2018	1 ♂		6	NEO	Panama (end)	PA
44. *Kiekiepanamensis* Polotow & Brescovit, 2018	4 ♂ 2 ♀ 2 imm		3, 6, 8, 9, 10	NEO	Panama (end)	PA
45. *Phoneutriadepilata* (Strand, 1909)	1 ♂ 1 ♀		6	NEO	Guatemala, Honduras, Nicaragua, Costa Rica, Panama, Colombia, Ecuador	CA-SA
46. Genus. 1 sp. 1	3 ♂ 2 ♀ 1 imm		6, 9			
**Fam. Cyrtaucheniidae**		SW				
47. *Bolostromuspanamanus* (Petrunkevitch, 1925)	1 ♂ 2 ♀		6, 9, 13	NEO	Costa Rica, Panama	CR-PA
**Fam. Filistatidae**		SW				
48. *Labahithamarginata* (Kishida, 1936)	1 ♀		13	COS	Taiwan, Philippines, Papua New Guinea, Pacifi Is. Introduced to Mexico, Central America, Brazil	MX-SA
**Fam. Gnaphosidae**		GH				
49. *Zimiromustropicalis* (Banks, 1909)	1 ♀		13	NEO	Costa Rica, Panama	CR-PA
**Fam. Hersiliidae**		SW				
50. *Neotamamexicana* (O. Pickard-Cambridge, 1893)	1 ♀ 1 imm		7, 9	NEO	USA to Peru, Guyana	NA-SA
**Fam. Lycosidae**		GH				
51. *Allocosacf.panamena*	4 ♂ 3 ♀ 1 imm		2, 8, 9			
52. *Arctosa* sp. 1	1 ♂		9			
53. *Hogna* sp. 1	2 ♂ 1 ♀		1, 3			
**Fam. Mimetidae**		SP				
54. *Gelanorzonatus* (C. L. Koch, 1845)	3 ♂ 1 imm		9	NEO	Mexico to Uruguay	MX-SA
55. *Mimetustrituberculatus* O. Pickard-Cambridge, 1899	3 ♂ 8 ♀ 2 imm		3, 6, 8, 9, 13	NEO	Panama (end)	PA
56. *Mimetusverecundus* Chickering, 1947	1 ♀		9	NEO	Panama (end)	PA
**Fam. Nephilidae**		OW				
57. *Trichonephilaclavipes* (Linnaeus, 1767)	1 ♂ 1 ♀		11	NEO	USA to Argentina. Introduced to São Tomé and Príncipe	NA-SA
**Fam. Oonopidae**		GH				
58. Costarinacf.recondita	1 ♀		8			
59. *Ponsoonops* sp. 1	1 ♂		8			
**Fam. Oxyopidae**		OH				
60. *Hamataliwa* sp. 1	1 imm		9			
**Fam. Pholcidae**		CW				
61. Modisimuscf.guatuso	1 ♂ 2 ♀ 2 imm		6, 8, 9			
62. *Modisimus* sp. 1	1 imm		6			
63. *Metagoniadelicata* (O. Pickard-Cambridge, 1895)	2 ♂		9	NEO	Mexico to Panama	MX-CA
64. *Metagonia* sp. 1	2 ♀^ca^		6			
65. *Physocyclus* sp. 1	3 ♀		3, 13			
**Fam. Salticidae**		OH				
66. *Acragaspeckhami* (Chickering, 1946)	3 ♂ 2 ♀		6, 7, 9, 11, 13	NEO	Panama, Colombia	PA-CO
67. *Anasaitiscanalis* (Chamberlin, 1925)	2 ♂ 5 ♀		6, 8, 11	NEO	Panama, Colombia	PA-CO
68. *Chapodagitae* * Zhang & Maddison, 2012	1 ♂		7	NEO	Colombia, Ecuador	CO-EC
69. *Chapodarecondita* (G. W. Peckham & E. G. Peckham, 1896)	2 ♂ 5 ♀ 1 imm		9	NEO	Guatemala, Costa Rica, Panama	GT, CR-PA
70. *Cobanusextensus* (G. W. Peckham & E. G. Peckham, 1896)	5 ♀		6, 11, 13	NEO	Panama (end)	PA
71. *Colonus* sp. 1	2 imm		6, 8			
72. *Corythaliaopima* (G. W. Peckham & E. G. Peckham, 1885)	1 ♂ 1 ♀ 1 imm		8, 9, 13	NEO	USA, Mexico, Guatemala, El Salvador	NA-CA
73. *Corythaliaspiralis* (F. O. Pickard-Cambridge, 1901)	2 ♂		6, 13	NEO	Colombia, Venezuela, French Guiana, Brazil	SA
						
74. *Corythaliasulphurea* (F. O. Pickard-Cambridge, 1901)	1 ♀		11	NEO	Costa Rica, Panama	CR-PA
75. *Habronattusmexicanus* (G. W. Peckham & E. G. Peckham, 1896)	1 ♀		1	NEO	USA to Panama, Caribbean	NA-C-CA
76. *Leptofreyabifurcata* (F. O. Pickard-Cambridge, 1901)	1 ♂		13	NEO	Mexico, Panama	MX, PA
77. *Lyssomanes* sp. 1	5 imm		3, 6, 8, 9			
78. *Menemerusbivittatus* (Dufour, 1831)	1 ♀		13	COS	Africa. Introduced to North, Central and South America, southern Europe, Turkey, India, China, Taiwan, Japan, Australia, Pacific Is.	NA-CA-SA
79. *Myrmapanapanamensis* (Galiano, 1969)	1 ♂ 1 imm		3	NEO	Panama, Argentina	PA, AR
80. *Noegusspiralifer* (F. O. Pickard-Cambridge, 1901)	1 ♂		9	NEO	Guatemala, Panama	GU, PA
81. *Psecas* sp. 1	1 imm		8			
82. *Sarindanigra* * G. W. Peckham & E. G. Peckham, 1892	1 ♂ 2 ♀ 1 imm		6	NEO	Nicaragua, Guyana, Brazil, Paraguay, Argentina	NI, SA
83. *Sidusaflavens* (G. W. Peckham & E. G. Peckham, 1896)	10 ♂ 1 imm		4, 5, 7, 8, 9, 11	NEO	Panama (end)	PA
84. *Xanthofreyaalbosignata* (F. O. Pickard-Cambridge, 1901)	1 ♂		9	NEO	Guatemala, Panama, Colombia, Brazil	GU, PA-SA
85. *Xanthofreyaarraijanica* (Chickering, 1946)	1 ♂		9	NEO	Panama, Colombia	PA, CO
**Fam. Scytodidae**		OH				
86. Scytodescf.intricata	3 ♂ 3 ♀ 7 imm		1, 3, 8, 9			
87. *Scytodesfusca* Walckenaer, 1837	2 ♂ 3 ♀ 4 imm		8, 9, 11, 13	NEO	Northern to Southern America. Introduced to Europe, Africa, Seychelles, India, Myanmar, China, Japan, Hawaii	NA-SA
88. *Scytodes* sp. 1	1 imm		6			
**Fam. Selenopidae**		AH				
89. *Selenops* sp. 1	2 imm		3, 9			
90. *Selenops* sp. 2	1 imm		6			
**Fam. Senoculidae**		OH				
91. *Senoculusrubicundus* Chickering, 1953	1 ♀ 10 imm		4, 6, 8, 9, 10, 11	NEO	Panama (end)	PA
**Fam. Sparassidae**		OH				
92. *Meri* sp. 1	1 imm		6			
93. *Meri* sp. 2	3 imm		9, 11			
94. *Nolavia* sp. 1	1 ♀		3			
95. *Nolaviastylifer* (F. O. Pickard-Cambridge, 1900)	1 ♂		6	NEO	Mexico, Brazil	MX, BR
96. *Sparianthischickeringi* (Gertsch, 1941)	1 ♀ 1 imm		8, 10	NEO	Panama (end)	PA
97. *Uaiuara* sp. 1	1 imm		9			
**Fam. Tetragnathidae**		OW				
98. *Dolichognathapentagona* (Hentz, 1850)	1 ♂ 1 ♀ 2 imm		8, 10	NEO	USA to Venezuela	NA-SA
99. *Dolichognathaspinosa* (Petrunkevitch, 1939)	4 ♂ 2 ♀ 5 imm		6, 9, 12, 13	NEO	Panama (end)	PA
100. *Leucaugesaphes* Chamberlin & Ivie, 1936	1 ♀ 1 imm		8, 9	NEO	Panama (end)	PA
101. *Leucauge* sp. 1	1 imm		11			
102. *Leucauge* sp. 2	2 ♀ 1 imm		12			
103. Metabuscf.debilis	1 ♂		13			
104. *Tetragnathacambridgei* Roewer, 1942	1 ♂		13	NEO	Mexico, Central America, Puerto Rico	MX-C-CA
105. *Tetragnathapallida* O. Pickard-Cambridge, 1889	3 ♂ 11 ♀ 1 imm		2, 3, 6, 8, 9, 11, 13	NEO	Costa Rica, Panama	CR, PA
106. *Tetragnathatenuissima* O. Pickard-Cambridge, 1889	2 ♂ 8 ♀ 1 imm		6, 9, 11	NEO	Mexico, Central America, Caribbean to Brazil, Argentina	MX-CA-C-SA
107. *Tetragnatha* sp. 3	7 imm		3, 6, 9, 11, 12			
**Fam. Theraphosidae**		GH				
108. *Sericopelma* sp. 1	1 ♀		8			
**Fam. Theridiidae**		CW				
109. *Ariamnesattenuatus* O. Pickard-Cambridge, 1881	1 ♂ 3 ♀ 1 imm		6, 9, 11	NEO	Costa Rica, Caribbean to Argentina	CR, C-SA
110. *Chryssoalbomaculata* O. Pickard-Cambridge, 1882	1 ♀		9	NEO	USA, Mexico to Brazil, Caribbean	NA-C-SA
111. Chryssocf.vallensis	1 ♂ 2 ♀		6, 9			
112. Cryptachaeacf.taeniata	6 ♀ 2 imm		6, 8, 10			
113. *Dipoena* sp. 1	1 ♂		11			
114. *Dipoena* sp. 2	1 ♀		8			
115. Emertonellacf.taczanowskii	1 ♂		6			
116. Episinuscf.pyrus	3 imm		8, 9, 10			
117. *Episinus* sp. 1	1 ♂		11			
118. *Episinus* sp. 2	2 ♂ 1 ♀		9			
119. *Hentziectypus* sp. 1	1 ♀		3			
120. *Neopisinusbruneoviridis* (Mello-Leitão, 1948)	3 ♀		6, 9, 13	NEO	Panama, Trinidad to Brazil	PA, C-BR
121. *Neopisinusputus* (O. Pickard-Cambridge, 1894)	9 ♂ 14 ♀10 imm		3, 4, 6, 8, 9, 10, 13	NEO	Mexico to Panama	MX-CA
122. *Neospintharus* sp. 1	1 ♂		9			
123. *Phycosomaaltum* (Keyserling, 1886)	1 ♂ 1 ♀		11, 13	NEO	Mexico to Brazil. Introduced to Hawaii	MX-BR
124. Rhomphaeacf.projiciens	3 ♀		5, 9			
125. *Rhomphaeametaltissima* Soares & Camargo, 1948	1 ♀		4	NEO	Panama to Brazil	PA-BR
126. *Rhomphaeaparadoxa* (Taczanowski, 1873)	3 ♂ 2 ♀ 1 imm		1, 4, 8, 9, 10	NEO	St. Vincent, Mexico to Brazil	MX-BR, C
127. *Theridion* sp. 1	1 ♀		11			
128. *Thwaitesiaaffinis* O. Pickard-Cambridge, 1882	2 ♂ 4 ♀ 5 imm^ca^		3, 4, 6, 9, 10	NEO	Panama to Paraguay	PA-SA
129. *Thwaitesia* sp. 1	2 imm		9, 11			
130. *Wambacrispulus* (Simon, 1895)	1 ♀		13	NEO	Canada to Brazil, Caribbean	NA-SA, C
**Fam. Theridiosomatidae**		OW				
131. *Epilineutesglobosus* (O. Pickard-Cambridge, 1896)	3 imm		9, 11	NEO	Mexico to Brazil	NA-SA
132. *Naatlo* sp. 1	1 ♂		9			
**Fam. Thomisidae**		AH				
133. *Epicadustaczanowskii* (Roewer, 1951)	1 ♂		9	NEO	Hispaniola, Costa Rica, Panama to Peru, Bolivia, Brazil	C, CR-SA
134. *Epicadustuberculatus* (Petrunkevitch, 1910)	1 ♂ 1 ♀		8, 11	NEO	Panama, Ecuador, Peru, Brazil	PA, EC,PE, BR
135. *Misumenoides* sp. 1	1 imm		5			
136. *Misumenoides* sp. 2	1 imm		1			
137. *Synema* sp. 1	1 imm		7			
138. *Tmarusineptus* O. Pickard-Cambridge, 1892	7 ♀ 5 imm		5, 6, 7, 9	NEO	Panama, Colombia	PA, CO
139. *Tmarusintentus* O. Pickard-Cambridge, 1892	1 ♀		6	NEO	Guatemala, Panama	GU, PA
140. *Tmarusprobus* Chickering, 1950	1 ♂		13	NEO	Panama (end)	PA
141. *Tmarus* sp. 1	1 ♀		8			
142. *Tmarusstudiosus* O. Pickard-Cambridge, 1892	2 ♀		2, 8	NEO	Panama (end)	PA
**Fam. Trachelidae**		GH				
143. *Trachelas* sp. 1	2 ♂		4, 9			
**Fam. Trechaleidae**		SP				
144. Cupienniuscf.granadensis	2 ♂ 6 ♀ 8 imm		3, 6, 8, 9			
145. *Cupienniuscoccineus* F. O. Pickard-Cambridge, 1901	1 imm		3	NEO	Costa Rica to Colombia	CR-CO
146. *Cupiennius* sp. 1	1 imm		9			
147. *Trechaelaextensa* (O. Pickard-Cambridge, 1896)	1 ♂, 4 imm		9, 11	NEO	Mexico to Panama	MX-CA
**Fam. Uloboridae**		OW				
148. *Miagrammopessimus* Chamberlin & Ivie, 1936	3 ♂ 1 ♀		8, 9	NEO	Panama (end)	PA
149. *Miagrammopes* sp. 1	1 ♀ 2 imm		9			
150. *Philoponellatingens* (Chamberlin & Ivie, 1936)	1 ♂ 3 ♀ 1 imm		8, 9, 13	NEO	Mexico to Colombia	MX-SA
151. *Uloborus* sp. 1	1 imm		2			
152. *Uloborustrilineatus* Keyserling, 1883	3 ♀ 4 imm		8, 9, 13	NEO	Mexico to Argentina	MX-SA
