# Supplementary material for: Preliminary checklist of spiders (Araneae) from Coiba National Park, Panama
Source: Biodivers Data J. 2024 Jul 31;12:e117642. doi: 10.3897/BDJ.12.e117642 (PMC11306922; doi:10.3897/BDJ.12.e117642)
Supplement: Supplementary material 1 — Supporting literature and figures [file bdj-12-e117642-s001.docx]

**Supplementary Material**

*Supporting literature for spider identification*

Arizala, S., Labarque, F. M. & Polotow, D. (2021). Revision of the Neotropical spider genus Acanthoctenus (Araneae: Ctenidae: Acanthocteninae). Zootaxa 4920(1): 1-55. [incl. Erratum: Zootaxa 5032(4): 600] doi:10.11646/zootaxa.4920.1.1

Barth, F. G. & Cordes, D. (2008). Key to the genus Cupiennius (Araneae, Ctenidae). Stapfia 88: 225-228.

Bayer, S., Höfer, H. & Metzner, H. (2020). Revision of the genus Corythalia C.L. Koch, 1850, part 1: diagnosis and new species from South America (Araneae: Salticidae: Salticinae: Euophryini). Zootaxa 4806(1): 1-144. doi:10.11646/zootaxa.4806.1.1

Bonaldo, A. B., Lise, A. A., Ramírez, M. J. & Saturnino, R. (2018). Revisiting the spider genus Eutichurus Simon, 1897 (Araneae, Eutichuridae): new species and complementary descriptions. Zootaxa 4382(2): 231-346. doi:10.11646/zootaxa.4382.2.6

Brescovit, A. D. & Rheims, C. A. (2001). Notes on the genus Scytodes (Araneae, Scytodidae) in Central and South America. Journal of Arachnology 29: 312-329.

Chickering, A. M. (1937). The Clubionidae of Barro Colorado Island, Panama. Transactions of the American Microscopical Society 56(1): 1-47. doi:10.2307/3222720

Chickering, A. M. (1940). New Anyphaenidae from Panama with notes on known species. Transactions of the American Microscopical Society 59(1): 78-122. doi:10.2307/3222819

Chickering, A. M. (1941). The Senoculidae of Panama. Papers of the Michigan Academy of Science, Arts and Letters 26: 195-218.

Chickering, A. M. (1946). The Salticidae of Panama. Bulletin of the Museum of Comparative Zoology 97: 1-474.

Chickering, A. M. (1947). The Mimetidae (Araneae) of Panama. Transactions of the American Microscopical Society 66(3): 221-248. doi:10.2307/3223388

Chickering, A. M. (1949). The Gnaphosidae of Panama. Transactions of the American Microscopical Society 68(4): 314-331. doi:10.2307/3223273

Chickering, A. M. (1950a). Three new dictynids (Araneae) from Panama. Papers of the Michigan Academy of Science, Arts and Letters 34: 85-96.

Chickering, A. M. (1950b). The spider genus Tmarus (Thomisidae) in Panama. Bulletin of the Museum of Comparative Zoology 103(4): 213-255, pl. 1-4.

Chickering, A. M. (1953). Two new species of Senoculus from Panama. Transactions of the American Microscopical Society 72(3): 281-287. doi:10.2307/3223456

Chickering, A. M. (1955). The genus Eustala (Araneae, Argiopidae) in Central America. Bulletin of the Museum of Comparative Zoology 112(6): 389-518.

Chickering, A. M. (1961). The genus Micrathena (Araneae, Argiopidae) in Central America. Bulletin of the Museum of Comparative Zoology 125(13): 389-470.

Chickering, A. M. (1965). Panamanian spiders of the genus Tmarus (Araneae, Thomisidae). Bulletin of the Museum of Comparative Zoology 133(7): 337-368.

Chickering, A. M. (1966). Five new species of the genus Tmarus (Araneae, Thomisidae) from the West Indies. Psyche, Cambridge 72(3, 1965): 229-240. doi:10.1155/1965/849804

Chickering, A. M. (1967). The genus Nops (Araneae, Caponiidae) in Panama and the West Indies. Breviora 274: 1-19.

Chickering, A. M. (1968). The genus Miagrammopes (Araneae, Uloboridae) in Panama and the West Indies. Breviora 289: 1-28.

Chickering, A. M. (1971). The genus Oonops (Araneae, Oonopidae) in Panama and the West Indies. Part 1. Psyche, Cambridge 77(4, 1970): 487-512. doi:10.1155/1970/93092

Chickering, A. M. (1972). The genus Oonops (Araneae, Oonopidae) in Panama and the West Indies. Part 3. Psyche, Cambridge 79(1-2): 104-115. doi:10.1155/1972/70621

Chickering, A. M. (1973a). The spider genus Trachelas (Araneae, Clubionidae) in the West Indies. Psyche, Cambridge 79(3, 1972): 215-230. doi:10.1155/1972/48060

Chickering, A. M. (1973b). The genus Corinna (Araneae, Clubionidae) in Panama. Psyche, Cambridge 79(4, 1972): 365-378. doi:10.1155/1972/75439

Chickering, A. M. **(1942)**. The Palpimanidae of Panama. *Papers of the Michigan Academy of Science, Arts and Letters* 27: 235-239.

Chickering, A. M. **(1943).** Twenty-one new species of *Dipoena* (Theridiidae) from Panama. *Transactions of the American Microscopical Society* 62(4): 329-378. [doi:10.2307/3222860](http://dx.doi.org/10.2307/3222860)

Chickering, A. M. **(1951)**. The Oonopidae of Panama. *Bulletin of the Museum of Comparative Zoology* 106(5): 205-245.

Chickering, A. M. **(1954).** The spider genus *Mangora* (Argiopidae) in Panama. *Bulletin of the Museum of Comparative Zoology* 111(5): 193-215.

Chickering, A. M. **(1956).** Three new species of Mimetidae (Araneae) from Panama. *Breviora* 57: 1-14.

Chickering, A. M. **(1957).** The genus *Tetragnatha* (Araneae, Argiopidae) in Panama. *Bulletin of the Museum of Comparative Zoology* 116(5): 299-354.

Dupérré, N. (2023b). Review of the American genus Bolostromus Ausserer, 1875 with the description of fourteen new species (Araneae, Cyrtaucheniidae). Zootaxa 5317(1): 1-88. doi:10.11646/zootaxa.5317.1.1

Edwards, G. B. (2015). Freyinae, a major new subfamily of Neotropical jumping spiders (Araneae: Salticidae). Zootaxa 4036(1): 1-87. doi:10.11646/zootaxa.4036.1.1

Exline, H. & Levi, H. W. (1962). American spiders of the genus Argyrodes (Araneae, Theridiidae). Bulletin of the Museum of Comparative Zoology 127: 75-204.

Galiano, M. E. (1965b). Salticidae (Araneae) formiciformes IV. Revisión del género Sarinda Peckham, 1892. Revista del Museo Argentino de Ciencias Naturales Bernardino Rivadavia (Ent.) 1: 267-312.

Hazzi, N. A. & Hormiga, G. (2021). Morphological and molecular evidence support the taxonomic separation of the medically important Neotropical spiders Phoneutria depilata (Strand, 1909) and P. boliviensis (F.O. Pickard-Cambridge, 1897) (Araneae, Ctenidae). ZooKeys 1022: 13-50. [incl. Corrigenda. ZooKeys 1033: 203-205. doi:10.3897/zookeys.1033.65850] doi:10.3897/zookeys.1022.60571

Höfer, H. & Brescovit, A. D. (2000). A revision of the Neotropical spider genus Ancylometes Bertkau (Araneae: Pisauridae). Insect Systematics & Evolution 31(3): 323-360. doi:10.1163/187631200X00075

Huber, B. A. (2000). New World pholcid spiders (Araneae: Pholcidae): A revision at generic level. Bulletin of the American Museum of Natural History 254: 1-348. doi:10.1206/0003-0090(2000)254<0001:NWPSAP>2.0.CO;2

Jäger, P. (2020c). The spider genus Olios Walckenaer, 1837 (Araneae: Sparassidae) – Part 1: species groups, diagnoses, identification keys, distribution maps and revision of the argelasius-, coenobitus- and auricomis-groups. Zootaxa 4866(1): 1-119. doi:10.11646/zootaxa.4866.1.1

Jocqué R and Dippenaar-Schoeman AS (2006). Spider families of the world. Tervuren, Belgium: Musée Royal de l'Afrique Centrale.

Levi, H. W. & Levi, L. R. (1962). The genera of the spider family Theridiidae. Bulletin of the Museum of Comparative Zoology 127: 1-71.

Levi, H. W. (1953). New and rare Dipoena from Mexico and Central America (Araneae, Theridiidae). American Museum Novitates 1639: 1-11.

Levi, H. W. (1954). Spiders of the genus Euryopis from North and Central America (Araneae, Theridiidae). American Museum Novitates 1666: 1-48.

Levi, H. W. (1955). The spider genera Episinus and Spintharus from North America, Central America and the West Indies (Araneae: Theridiidae). Journal of the New York Entomological Society 62(2, 1954): 65-90.

Levi, H. W. (1957). The spider genera Chrysso and Tidarren in America. Journal of the New York Entomological Society 63: 59-81.

Levi, H. W. (1959). The spider genera Achaearanea, Theridion and Sphyrotinus from Mexico, Central America and the West Indies (Araneae, Theridiidae). Bulletin of the Museum of Comparative Zoology 121: 57-163.

Levi, H. W. (1963a). American spiders of the genera Audifia, Euryopis and Dipoena (Araneae: Theridiidae). Bulletin of the Museum of Comparative Zoology 129(2): 121-185, pl. 1-12.

Levi, H. W. (1963b). American spiders of the genus Theridion (Araneae, Theridiidae). Bulletin of the Museum of Comparative Zoology 129: 481-589.

Levi, H. W. (1976). The orb-weaver genera Verrucosa, Acanthepeira, Wagneriana, Acacesia, Wixia, Scoloderus and Alpaida north of Mexico. Bulletin of the Museum of Comparative Zoology 147: 351-391.

Levi, H. W. (1977). The American orb-weaver genera Cyclosa, Metazygia and Eustala north of Mexico (Araneae, Araneidae). Bulletin of the Museum of Comparative Zoology 148: 61-127.

Levi, H. W. (1986a). The Neotropical orb-weaver genera Chrysometa and Homalometa (Araneae: Tetragnathidae). Bulletin of the Museum of Comparative Zoology 151(3): 91-215.

Levi, H. W. (1986b). The orb-weaver genus Witica (Araneae: Araneidae). Psyche, Cambridge 93(1-2): 35-46. doi:10.1155/1986/93154

Levi, H. W. (1992). Spiders of the orb-weaver genus Parawixia in America (Araneae: Araneidae). Bulletin of the Museum of Comparative Zoology 153: 1-46.

Levi, H. W. (1993). The Neotropical orb-weaving spiders of the genera Wixia, Pozonia, and Ocrepeira (Araneae: Araneidae). Bulletin of the Museum of Comparative Zoology 153: 47-141.

Levi, H. W. (1995). The Neotropical orb-weaver genus Metazygia (Araneae: Araneidae). Bulletin of the Museum of Comparative Zoology 154: 63-151.

Levi, H. W. (1997). The American orb weavers of the genera Mecynogea, Manogea, Kapogea and Cyrtophora (Araneae: Araneidae). Bulletin of the Museum of Comparative Zoology 155: 215-255.

Levi, H. W. (1999). The Neotropical and Mexican Orb Weavers of the genera Cyclosa and Allocyclosa (Araneae: Araneidae). Bulletin of the Museum of Comparative Zoology 155: 299-379.

Levi, H. W. (2002). Keys to the genera of araneid orbweavers (Araneae, Araneidae) of the Americas. Journal of Arachnology 30(3): 527-562. doi:10.1636/0161-8202(2002)030[0527:KTTGOA]2.0.CO;2

Metzner, H. (2023): Jumping spiders (Arachnida: Araneae: Salticidae) of the world. Online at <https://www.jumping-spiders.com>

Opell, B. D. (1979). Revision of the genera and tropical American species of the spider family Uloboridae. Bulletin of the Museum of Comparative Zoology 148: 443-549.

Polotow, D. & Brescovit, A. D. (2018). Kiekie, a new Neotropical spider genus of Ctenidae (Cteninae, Araneae). Zootaxa 4531(3): 353-373. doi:10.11646/zootaxa.4531.3.2

Prószyński, J. (2016). Monograph of Salticidae (Araneae) of the World 1995-2015. Part II. Global Species Database of Salticidae (Araneae). Version October 30th, 2016, online at <http://www.salticidae.pl>.

Rheims, C. A. & Brescovit, A. D. (2004a). Revision and cladistic analysis of the spider family Hersiliidae (Arachnida, Araneae) with emphasis on Neotropical and Nearctic species. Insect Systematics & Evolution 35(2): 189-239. doi:10.1163/187631204788912355

Rheims, C. A. & Jäger, P. (2022). Revalidation of the genus Sadala Simon, 1880 with the description of a new genus of Neotropical huntsman spiders (Araneae, Sparassidae). Zootaxa 5135(1): 1-80. doi:10.11646/zootaxa.5135.1.1

Rheims, C. A. (2020). Revision of the spider genus Sparianthis Simon, 1880 (Araneae, Sparassidae, Sparianthinae). Zootaxa 4890(2): 151-191. doi:10.11646/zootaxa.4890.2.1

Saturnino, R. & Bonaldo, A. B. (2015). Taxonomic review of the New World spider genus Elaver O. Pickard-Cambridge, 1898 (Araneae, Clubionidae). Zootaxa 4045(1): 1-119. doi:10.11646/zootaxa.4045.1.1

Silva-Dávila, D. (2003). Higher-level relationships of the spider family Ctenidae (Araneae: Ctenoidea). Bulletin of the American Museum of Natural History 274: 1-86. doi:10.1206/0003-0090(2003)274<0001:HLROTS>2.0.CO;2

Ubick, D., Paquin, P., Cushing, P. E., & Roth, V. D. (Eds.). (2017). Spiders of North America: an identification manual. American Arachnological Society.

Vanuytven, H. (2021). The Theridiidae of the World. A key to the genera with their diagnosis and a study of the body length of all known species. Newsletter of the Belgian arachnological Society 35(Supplement): 1-363

Zhang, J. X. & Maddison, W. P. (2012c). New euophryine jumping spiders from Central and South America (Araneae: Salticidae: Euophryinae). Zootaxa 3578: 1-35. doi:10.11646/zootaxa.3578.1.1

Figure S1. *Ctenus nigrolineatus*, female, dorsal view (A). Male, dorsal view (B). Female epigynum, dorsal view (C). Male left palp, ventral and retrolateral view (D). Scale bars: A, C, 1 mm; B, D, 2.6 mm.

Figure S2. *Chapoda gitae* Male, dorsal view (A). Male right palp, ventral view (B). Male right palp, retrolateral view (C). *Sarinda nigra* male, dorsal view (D). Male left palp ventral view (E). Scale bars: A, D and E correspond to 1 mm; B, C correspond to 2.6 mm.
