# Supplementary material for: Preliminary checklist of spiders (Araneae) from Coiba National Park, Panama
Source: Biodivers Data J. 2024 Jul 31;12:e117642. doi: 10.3897/BDJ.12.e117642 (PMC11306922; doi:10.3897/BDJ.12.e117642)
Supplement: Supplementary material 2 — Table S1. [file bdj-12-e117642-s002.docx]

Table S1. Species richness and abundance per sampling site in Coiba National Park. See main text for details of each sites and methods used for sampling.

| **Location** | **visits** | **richness** | **abundance** |
| --- | --- | --- | --- |
| 1. Old main prison | 1 | 6 | 7 |
| 2. Coiba Scientific Station | 2 | 6 | 10 |
| 3. MiAmbiente Station | 3 | 18 | 26 |
| 4. Mirador Alto | 2 | 13 | 16 |
| 5. Mirador Gambute | 1 | 6 | 6 |
| 6. Playa Hermosa | 6 | 52 | 121 |
| 7. San Juan | 1 | 8 | 15 |
| 8. Sendero de Coiba AIP | 5 | 43 | 94 |
| 9. Sendero Los Monos | 10 | 79 | 196 |
| 10. Sendero Santa Cruz | 2 | 15 | 19 |
| 11. Canales Afuera Island | 2 | 25 | 44 |
| 12. Jicaron Island | 7 | 34 | 56 |
| 13. Coibita Island | 1 | 29 | 32 |
